# Incremental effects of SGLT2 inhibitors, GLP-1 receptor agonists, and pioglitazone in type 2 diabetes and MASLD across Asian study settings: a systematic review and meta-analysis

**DOI:** 10.3389/fendo.2026.1942151

**Published:** 2026-09-01

**Authors:** Hanwen Zheng, Jieru Han

**Affiliations:** 1 First Clinical Medical College, Heilongjiang University of Chinese Medicine, Harbin, China; 2 School of Basic Medical Sciences, Heilongjiang University of Chinese Medicine, Harbin, China

**Keywords:** add-on therapy, Asian study settings, GLP-1 receptor agonists, meta-analysis, metabolic dysfunction-associated steatotic liver disease, pioglitazone, SGLT2 inhibitors, type 2 diabetes mellitus

## Abstract

**Objective:**

We evaluated the incremental hepatic and metabolic effects of adding sodium–glucose cotransporter 2 (SGLT2) inhibitors, glucagon-like peptide-1 receptor agonists (GLP-1RAs), or pioglitazone to comparable background glucose-lowering therapy in Asian adults with type 2 diabetes mellitus (T2DM) and metabolic dysfunction-associated steatotic liver disease (MASLD).

**Methods:**

We systematically searched PubMed, Embase, the Cochrane Library, and Web of Science through 30 June 2026. Two reviewers independently screened studies, extracted data, and assessed risk of bias. Eligible trials evaluated the addition of a study drug to background glucose-lowering management that was comparable between randomized groups and either remained stable or followed a prespecified adjustment protocol. Changes from baseline in alanine aminotransferase (ALT) and glycated hemoglobin (HbA1c) were coprimary outcomes. Secondary outcomes included aspartate aminotransferase, gamma-glutamyl transferase, magnetic resonance imaging–proton density fat fraction, liver stiffness, fibrosis-4 index, fasting plasma glucose, lipid parameters, body mass index, serum adiponectin, and systolic blood pressure. Random-effects pairwise meta-analyses were performed, and GRADE certainty was assessed for the two coprimary outcomes. Safety outcomes were synthesized narratively because reporting was heterogeneous and frequently incomplete.

**Results:**

We included eleven randomized trials: 692 participants were randomized into eligible comparisons, of which 642 participated in the coprimary efficacy analyses. Moderate certainty was found for a reduction in ALT with the addition of an SGLT2 inhibitor compared with background therapy alone (mean difference [MD], − 7.56 U/L; 95% confidence interval [CI], − 11.24 to − 3.88); however, there was little or no clinically important further benefit observed for HbA1c (MD, − 0.09%; 95% CI, − 0.27 to 0.08; moderate certainty). Modest reductions in ALT were suggested by moderate certainty for GLP-1RAs (MD, − 6.71 U/L; 95% CI, − 11.53 to − 1.89) while low certainty indicated an HbA1c reduction (MD, − 0.48%; 95% CI, − 0.83 to − 0.14). Secondary exploratory findings favoring add-on therapy existed, although they are unadjusted for multiplicity. The comparative safety could not be assessed reliably.

**Conclusion:**

In short-term randomized trials conducted in Asian study settings, adding SGLT2 inhibitors or GLP-1RAs may improve selected hepatic and metabolic surrogate outcomes. The available evidence does not support a treatment-class ranking, and the durability and clinical significance of these changes remain uncertain because follow-up was short and several comparisons were based on sparse evidence. Comparative safety also remains uncertain because adverse-event reporting was heterogeneous and incomplete.

**Systematic review registration:**

https://www.crd.york.ac.uk/PROSPERO/, identifier CRD420251231137.

## 1 Introduction

Metabolic dysfunction-associated steatotic liver disease (MASLD; previously known as nonalcoholic fatty liver disease) shares pathophysiologic similarities such as insulin resistance, ectopic lipid accumulation, oxidative stress, and chronic low-grade inflammation with type 2 diabetes mellitus (T2DM) (1). There were an estimated 537 million people worldwide who lived with diabetes in 2021, a number that is projected to increase to 783 million by 2045 (2). Approximately two-thirds of those with T2DM are estimated to have MASLD (3); therefore, there is great interest in therapies targeting both glycemic control and liver-related outcomes (4).

Clinical phenotypes of MASLD vary across populations. Compared with Western populations, some Asian populations may develop hepatic steatosis and metabolic abnormalities at lower body mass index (BMI), a phenotype often described as “lean MASLD” (5–7). Differences in visceral adiposity, pancreatic insulin secretory capacity, and susceptibility to ectopic lipid deposition may contribute to differences in disease presentation and progression. Sodium–glucose cotransporter 2 (SGLT2) inhibitors and glucagon-like peptide-1 receptor agonists (GLP-1RAs) have shown favorable hepatic effects in patients with coexisting MASLD and T2DM (8); however, much of the available evidence comes from non-Asian or ethnically heterogeneous cohorts, limiting its generalizability to Asian study settings.

Today’s glucose-lowering strategies are often escalated/reformatted to meet goals beyond glycemia, including weight, cardiovascular, renal, and liver concerns. As such, SGLT2 inhibitors, GLP-1RAs, and sometimes pioglitazone are used atop existing therapy (rather than only as rescue when all baseline management fails) (9–12). In this context, an add-on approach applies particularly well to patients with T2DM and MASLD where we ask what additional benefit there is from adding one particular agent while continuing other appropriate background therapy. Background glucose-lowering management must be similar across randomized groups and either unchanged or subject to a prespecified adjustment protocol during trials—otherwise, changing concomitant therapies could confound results associated with our randomized strategy. Our clinically meaningful estimand is thus the incremental effect of the added drug/treatment strategy compared to otherwise comparable background management rather than its efficacy against a heterogeneous “usual-care” regimen.

However, incremental add-on effects with similar background therapy are poorly defined. Eligibility criteria, disease definition, baseline metabolic characteristics, length of treatment, outcome measures, and design of comparison differ substantially among trials and meta-analyses (13–15); many evidence syntheses pool placebo, lifestyle intervention, usual care, and active glucose-lowering comparators while not balancing background therapy across randomized groups (14–16), which makes attributing specific hepatic and metabolic effects to an added agent challenging. The generalizability is unclear, as most reviews involve a geographically and ethnically heterogeneous population without separate evaluation for Asian study settings. There are few direct comparisons (especially those involving SGLT2 inhibitors vs. GLP-1RAs); thus, any class difference appears primarily from indirect evidence rather than establishing comparative superiority (17–21).

Therefore, we conducted a systematic review and pairwise meta-analyses of randomized controlled trials conducted in Asian countries that evaluated the incremental hepatic and metabolic effects of adding an SGLT2 inhibitor, a GLP-1RA, or pioglitazone to comparable background glucose-lowering management in adults with T2DM and MASLD. We examined liver enzymes, markers of hepatic steatosis and fibrosis, glycemic control, lipid parameters, and anthropometric outcomes.

## 2 Methods

The original protocol was registered in International Prospective Register of Systematic Reviews (PROSPERO) (CRD420251231137) for a network meta-analysis of three pharmacological treatments in patients with T2DM and nonalcoholic fatty liver disease. Global inconsistency was assessed using the design-by-treatment interaction model, while local inconsistency was evaluated using the node-splitting approach. A *p*-value < 0.05 was considered to indicate statistically significant inconsistency. The planned network meta-analysis was not pursued after statistically significant inconsistency was identified between direct and indirect evidence (*p* < 0.05). The analysis was therefore revised to conventional pairwise meta-analysis based on direct comparisons. The protocol for the present analysis was retrospectively registered in PROSPERO (CRD420261457952).

### 2.1 Search strategy

We searched PubMed, Embase, the Cochrane Library, and Web of Science through 30 June 2026 for randomized controlled trials of SGLT2 inhibitors, GLP-1RAs, or pioglitazone in patients with T2DM and MASLD. The search combined Medical Subject Headings (MeSH) and free-text terms for T2DM, MASLD or nonalcoholic fatty liver disease, the three therapeutic classes, and randomized trials using Boolean operators. The full search strategies are provided in **Supplementary Table 1**. Two reviewers independently screened titles, abstracts, and full texts. Disagreements were resolved by discussion and consensus.

### 2.2 Criteria for selection and eligibility

Studies were eligible if they met all of the following criteria: (1) participants were adults in Asian study settings with T2DM and MASLD or nonalcoholic fatty liver disease; (2) the study was a prospective randomized controlled trial with at least 12 weeks of follow-up; (3) background glucose-lowering management was comparable between randomized groups at baseline and was either maintained during follow-up or managed according to a prespecified protocol; (4) the randomized contrast evaluated the addition of an SGLT2 inhibitor, a GLP-1RA, pioglitazone, or an active comparator on otherwise comparable background management, with any protocol-specified changes in concomitant therapy documented rather than treated as unplanned cointerventions; and (5) at least one prespecified primary or secondary outcome was reported.

We excluded studies that were nonrandomized, had an enrolled non-Asian study population, used noncomparable background treatment among randomization groups, allowed for unplanned changes of background medication, used concomitant glucose-lowering and/or hepatoprotective therapies potentially affecting the outcomes, reported duplicate/overlapping datasets, and provided inadequate outcome data.

### 2.3 Quality assessment

Two reviewers independently assessed risk of bias using the Cochrane Risk of Bias 2 (RoB 2) tool. As RoB 2 judgments are outcome-specific, separate assessments were performed for each randomized comparison contributing to the coprimary outcomes of change from baseline in alanine aminotransferase (ALT) and glycated hemoglobin (HbA1c). The target estimand was the effect of assignment to intervention. Assessments covered the five standard RoB 2 domains and were informed by published reports and, when available, **Supplementary Materials**, protocols, statistical analysis plans, and trial registries. Domain-level and overall judgments were classified as low risk of bias, some concerns, or high risk of bias according to the RoB 2 signaling questions and decision algorithms.

We did not consider an open-label design alone to indicate increased risk of bias. Instead, we evaluated whether knowledge of treatment allocation could plausibly influence deviations from intended interventions or outcome measurement. ALT and HbA1c were assessed separately with respect to missing outcome data and selective reporting. Disagreements were resolved by discussion and consensus. Full signaling-question responses, domain-level judgments, and study-specific justifications are provided in **Supplementary Tables 2**, **3**.

### 2.4 Data extraction

Two independent reviewers used a standard Microsoft Excel form to extract study design, participant demographics, diagnostic criteria, baseline clinical characteristics, intervention and comparator regimens, arm-level background glucose-lowering therapy (drug or class, dose if explicitly reported, prerandomization stability requirements, and protocol-specified adjustment rules), follow-up duration, and prespecified outcomes. Disagreements were discussed and settled through consensus. Safety data comprised adverse events, serious/severe adverse events, discontinuation because of adverse events, and other reason(s) for withdrawal. When unreported arm-level background agents/doses were noted, we recorded them as not reported (NR) instead of inferring from the information that is available. Detailed arm-level study-drug regimens and background-therapy management can be found in **Supplementary Table 8A**; **Supplementary Table 8** summarizes study design, follow-up, eligibility, participant populations, and outcomes contributing to our meta-analysis. We extracted population counts separately for randomized participants, treated/safety populations where explicitly reported, those who completed follow-up when available, and those analyzed per outcome. Where no treated denominator could be inferred because it was not separately reported in the original study, this was reflected accordingly.

Continuous outcome data were extracted preferentially as means and standard deviations (SDs). When studies reported medians with ranges and/or interquartile ranges rather than means and SDs, corresponding means and SDs were estimated using validated conversion methods (22–24). For change-from-baseline outcomes, when the SD of the change score was not reported, but baseline and posttreatment SDs were available, the change-score SD was calculated according to the Cochrane Handbook as SD_change = √(SD_baseline^2^ + SD_post^2^ − 2r × SD_baseline × SD_post), where *r* denotes the within-participant correlation between baseline and posttreatment measurements (25). A within-participant correlation coefficient of *r* = 0.8 was assumed for these calculations. When outcome data were reported as standard errors (SEs), they were converted to SDs using SD = SE × √*n* (25).

The coprimary endpoints were changes from baseline in ALT (the principal hepatic biochemical endpoint) and HbA1c (the principal glycemic endpoint). Secondary hepatic endpoints were aspartate aminotransferase (AST), gamma-glutamyl transferase, magnetic resonance imaging–proton density fat fraction (MRI-PDFF), liver stiffness measurement, and fibrosis-4 index. Secondary metabolic endpoints were fasting plasma glucose, triglycerides, high-density lipoprotein cholesterol, low-density lipoprotein cholesterol, BMI, serum adiponectin, and systolic blood pressure.

### 2.5 Data synthesis and analysis

Statistical analyses were performed using the meta package in R version 4.0.3 and Stata/MP version 17. Continuous outcomes were summarized as mean differences (MDs) with 95% confidence intervals (CIs). Pairwise meta-analyses used inverse-variance random-effects models, with between-study variance (τ^2^) estimated using the DerSimonian–Laird method. We report τ^2^ for pooled analyses when it was estimable; τ^2^ was not reported for single-study comparisons.

For multiarm trials, only pairwise comparisons relevant to the prespecified analyses were included, and no participant group was included more than once in the same meta-analysis, thereby avoiding double-counting of shared groups. The comparison categories displayed in each forest plot represented distinct prespecified pairwise treatment contrasts rather than clinical or methodological subgroups. No clinical or methodological subgroup analyses or formal interaction tests for subgroup effects were performed, and software-generated tests for differences among the displayed comparison categories were not used for inferential purposes.

Statistical heterogeneity was assessed using Cochran’s *Q* test and *I*^2^ statistic (*p* < 0.10 and *I*^2^ ≥ 50%, respectively). The forest plot indicates the direction of benefit: for outcomes for which lower values are favorable, such as ALT, AST, MRI-PDFF, liver stiffness, HbA1c, fasting plasma glucose, triglycerides, low-density lipoprotein cholesterol, systolic blood pressure, and body weight, values to the left side of the null line favor the experimental or add-on therapy. For outcomes for which higher values are favorable, such as high-density lipoprotein cholesterol and adiponectin, values to the right side of the null line favor the experimental or add-on therapy.

Leave-one-out analyses were conducted to assess the influence of individual studies on the pooled estimates. Sensitivity analyses were performed for all outcomes to evaluate the robustness of the pooled estimates. Assessment of publication bias was planned using visual inspection of funnel plots only when sufficient studies were available. As most pooled analyses included fewer than 10 studies, funnel plots were considered exploratory and were not interpreted as evidence to exclude publication bias.

Analyses of the two coprimary outcomes were prioritized. Secondary outcomes were considered exploratory; nominal *p*-values were reported without formal adjustment for multiplicity and were interpreted cautiously.

As adverse-event definitions and reporting formats were heterogeneous and arm-level data were frequently unavailable, safety outcomes were synthesized narratively rather than pooled quantitatively.

### 2.6 Certainty of evidence

Certainty of evidence for the two coprimary outcomes (ALT and HbA1c) within each prespecified pairwise comparison was assessed using the Grading of Recommendations Assessment, Development and Evaluation (GRADE) approach (26). Randomized evidence started at high certainty and was downgraded, when appropriate, for risk of bias, inconsistency, indirectness, imprecision, and publication bias. For the risk-of-bias domain, GRADE judgments were based on the final outcome-specific RoB 2 assessments for the corresponding coprimary outcome. Secondary outcomes were exploratory and were not formally graded. Imprecision was assessed primarily by comparing the 95% CI with a prespecified clinically important threshold and by evaluating the optimal information size (OIS), rather than by sample size or whether the CI crossed the null alone. A pragmatic between-group threshold of 0.5 percentage points was used for HbA1c. For ALT, a reduction of 17 U/L was used as a contextual histology-associated response threshold based on AASLD guidance (27). As this is not a validated between-group minimally important difference and may be mechanism-specific, it was used only to define the GRADE imprecision boundary and not to infer histological benefit. OIS was calculated for a two-group comparison using a two-sided *α* = 0.05, 80% power, the corresponding threshold, and the pooled within-group SD of change scores from contributing trial arms. Certainty was rated as high, moderate, low, or very low. Detailed GRADE evidence profiles and reasons for downgrading ALT and HbA1c are provided in **Supplementary Tables 4**-**7** for SGLT2 inhibitors versus background therapy, GLP-1RAs versus background therapy, SGLT2 inhibitors versus thiazolidinediones, and GLP-1RAs versus thiazolidinediones, respectively.

## 3 Results

### 3.1 Description of included studies

**Figure 1:**
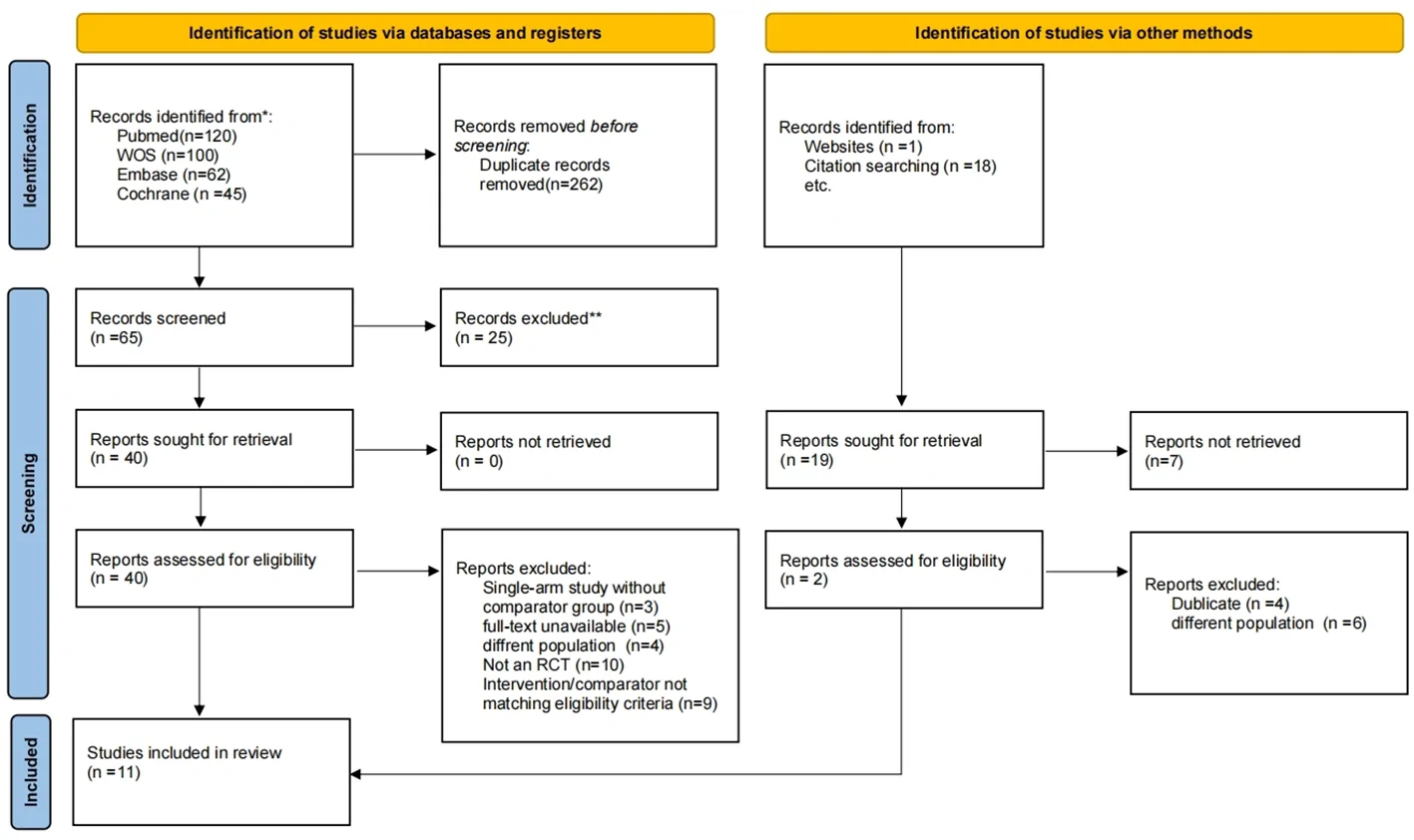
PRISMA flow diagram of study identification and selection.

We identified 327 records. After deduplication and sequential title, abstract, and full-text screening, 11 randomized controlled trials (RCTs) were included in the qualitative and quantitative syntheses (**Figure 1**). ALT and HbA1c were the coprimary outcomes; all other endpoints were analyzed as exploratory secondary outcomes.

In each comparison arm, a total of 692 participants were randomized, with 642 contributing to the coprimary efficacy analyses. Participant ages ranged from 46.6 to 62.3 years among all included trials. Differences in totals reflect trial-specific attrition and analysis populations reported in the original trials; treated/safety populations were captured if explicitly reported, and otherwise coded as NR. SGLT2 inhibitors, GLP-1RAs, and pioglitazone interventions were included. Comparison arms comprised continued matched background therapy, placebo on matched background therapy, and active comparators including pioglitazone. Background glucose-lowering regimens differed across trials. Doses per arm of study drugs along with background-therapy details and protocol-stated stability/adjustment rules are provided in **Supplementary Table 8A**; background drug doses are reported where explicitly available and otherwise shown as NR. Eligible trials either preserved background therapy or applied prespecified management rules that allowed us to interpret these contrasts as incremental randomized treatment strategies versus effects of unplanned comedication. Median follow-up duration was 24 weeks (range: 12–28 weeks); trials took place in China (*n* = 3), Japan (*n* = 4), India (*n* = 2), Iran (*n* = 1), and Thailand (*n* = 1). Details regarding studies and participants are presented in **Table 1** and **Supplementary Table 8**.

**Table 1:** Study characteristics, participant populations, treatment regimens, and follow-up periods of included randomized controlled trials.

Study (year)	Location	Participant populations (*n*; I/C)			Age (years; I/C)	Female (*n*)	Intervention (dose/frequency)	Comparator (dose/frequency)	Duration (weeks)
		R	T	A					
Ito (2017) (28)	Japan	32/34	NR	32/34 (ITT)	57.3 ± 12.1/59.1 ± 9.8	34	Ipragliflozin 50 mg/day + background therapy	Pioglitazone 15–30 mg/day + background therapy	24
Kinoshita (2020) (14)	Japan	40/36	NR	32/33	58.7 ± 1.6/58.0 ± 2.3	53	Dapagliflozin 5 mg/day + background therapy	Pioglitazone 7.5–15 mg/day + background therapy	28
Shi (2023) (15)	China	42/42	NR	40/38	49 ± 9.15/47.4 ± 10	24	Dapagliflozin 10 mg/day + metformin-based background therapy	Matched metformin-based background therapy without dapagliflozin	24
Kuchay (2020) (19)	India	32/32	NR	27/25	46.6 ± 9.1/48.1 ± 8.9	45	Dulaglutide 0.75 mg weekly for 4 weeks, then 1.5 mg weekly for 20 weeks + standard diabetes care	Standard diabetes care without a GLP-1RA	24
Attaran (2023) (29)	Iran	37/36	NR	37/36 (ITT)	52 ± 7/52 ± 7		Empagliflozin 10 mg/day + background therapy	Pioglitazone 30 mg/day + background therapy	24
Kuchay (2018) (30)	India	25/25	NR	22/20	50.7 ± 12.8/49.1 ± 10.3	17	Empagliflozin 10 mg/day + background therapy	Background diabetes therapy	20
Guo (2020) (31)	China	32/32^*^	NR	31/30	53.1 ± 6.3/52.6 ± 3.9	25	Liraglutide 1.8 mg/day + metformin-based background therapy	Placebo + matched metformin-based background therapy	26
Horibe (2022) (32)	Japan	52 total^†^	NR	26/24	59.7 ± 12.0/62.3 ± 6.5	18	Dapagliflozin 5 mg/day + standard treatment	Standard treatment without an SGLT2 inhibitor	24
Zhang (2020) (33)	China	30/30	NR	30/30	57 ± 16/60 ± 22	32	Liraglutide 0.6 mg/day (week 1), then 1.2 mg/day (weeks 2–24) + metformin + diet/exercise advice	Pioglitazone 15 mg/day (week 1), then 30 mg/day (weeks 2–24) + metformin + diet/exercise advice	24
Shimizu (2019) (34)	Japan	35/28	NR	33/24	56.2 ± 11.5/57.1 ± 13.8	23	Dapagliflozin 5 mg/day + standard diabetes care	Standard diabetes care without an SGLT2 inhibitor	24
Phrueksotsai (2021) (35)	Thailand	20/20	NR	18/20	57 ± 6.9/61.2 ± 7.2		Dapagliflozin 10 mg/day + stable oral antidiabetic drugs	Placebo + stable oral antidiabetic drugs	12

*R*, randomized population; *T*, treated/safety population; *A*, analyzed population; *ITT*, intention-to-treat; *NR*, not reported. The count of eligible comparison arms is given. "*" indicates, in Guo et al., there were three arms: the randomized count presented here corresponds to the liraglutide and placebo arms that entered into the prespecified pairwise comparison. †" indicates that, in Horibe et al., there were 52 randomized participants overall; because an arm-level randomized denominator was not available, the analyzed population was used as the denominator for the efficacy analysis, which may differ from the randomized population. Background therapy details at each arm level, reported background drug dose(s), and protocol-stated stability/adjustment rules are summarized in **Supplementary Table 8A**; unreported doses were not inferred.

### 3.2 Risk of bias

**Figure 2:**
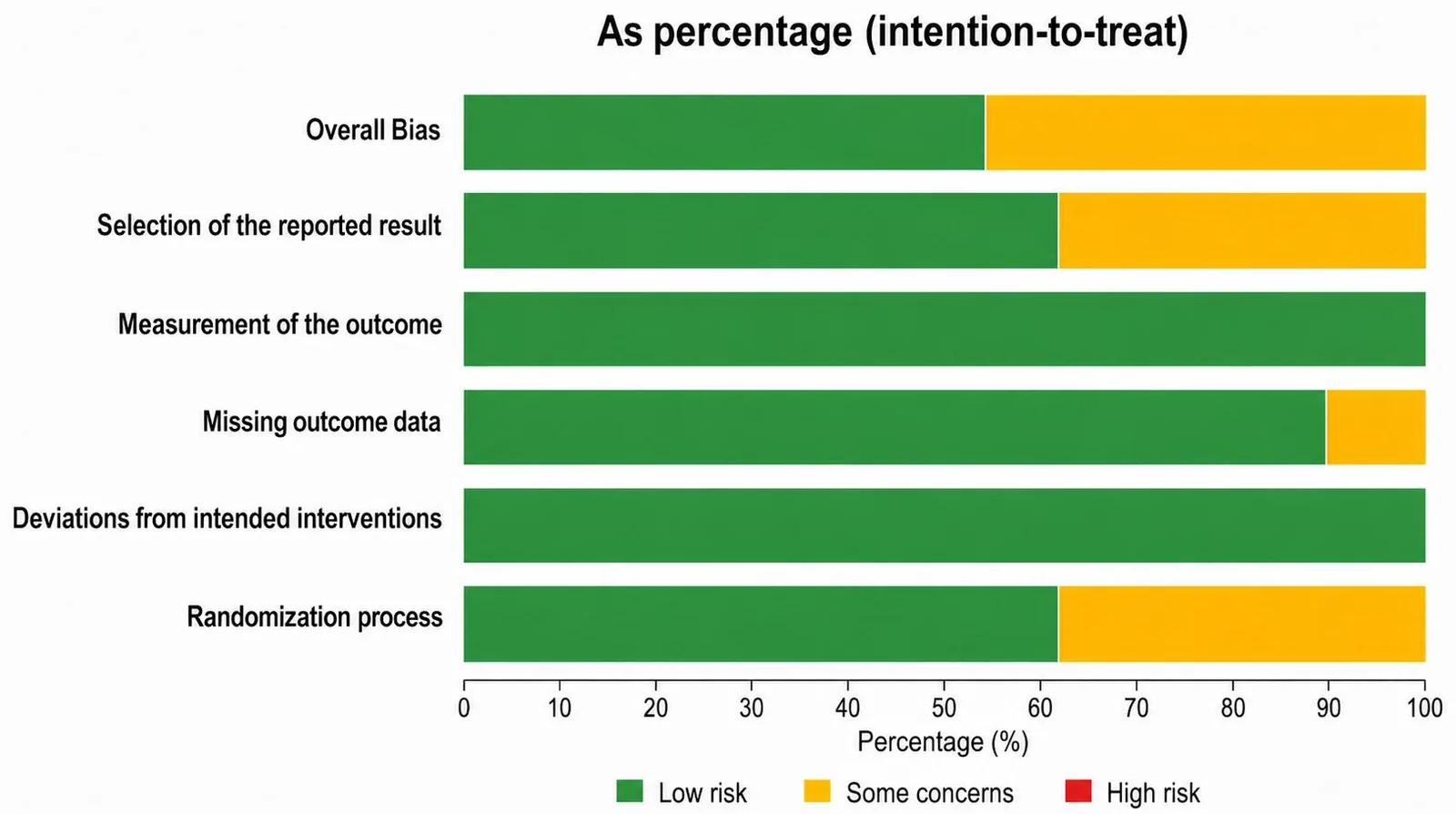
Risk-of-bias graph showing the proportions of judgments for each RoB 2 domain across included studies.

**Figure 3:**
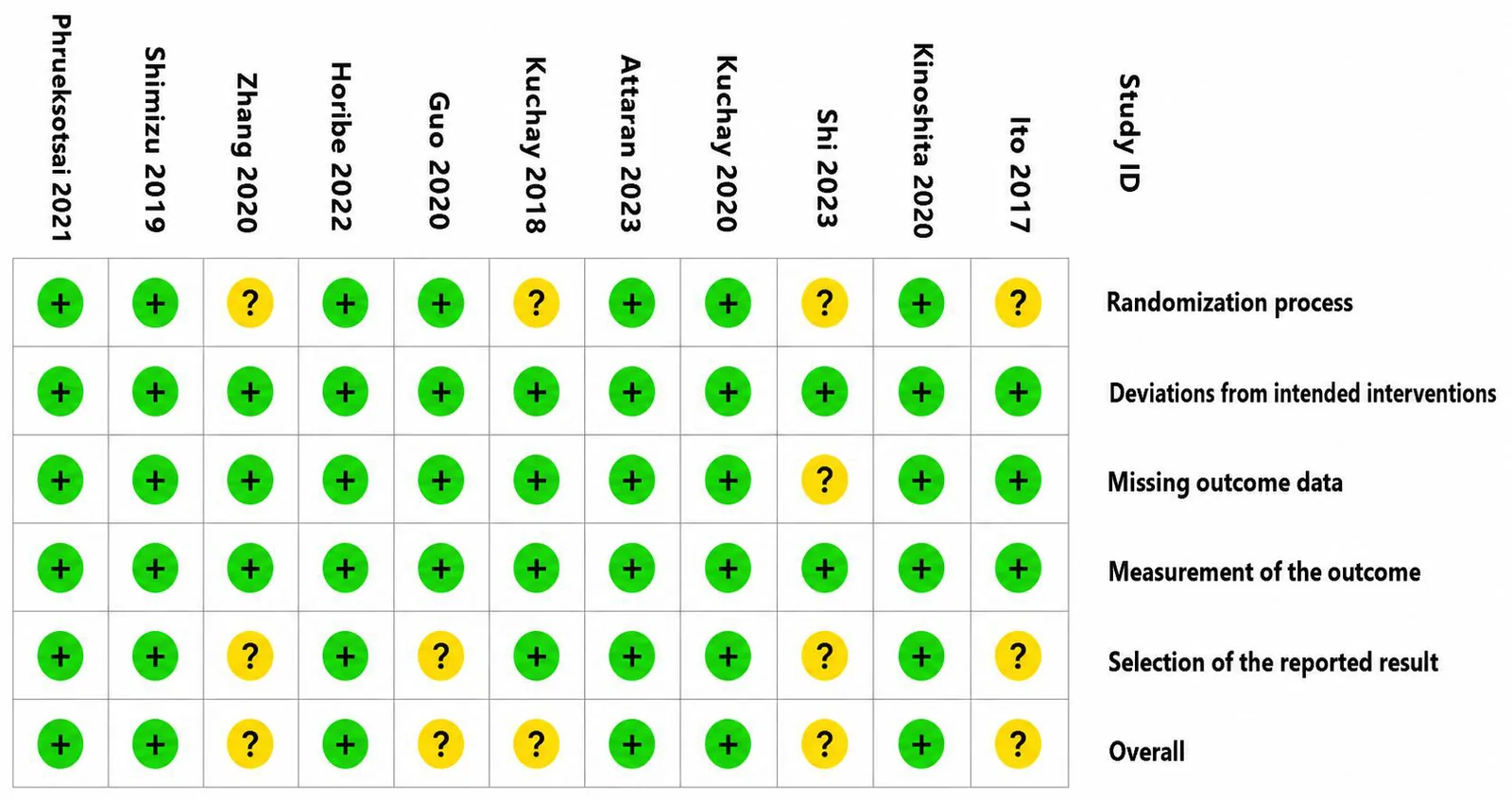
Risk-of-bias summary showing the review authors’ judgments for each RoB 2 domain across the included studies.

Outcome-specific RoB 2 assessments for the two coprimary outcomes are presented in **Figures 2**, **3** and **Supplementary Tables 2**, **3**. For ALT, six of 11 results were judged at low risk of bias and five as having some concerns; for HbA1c, six of 11 results were judged at low risk and five as having some concerns. No result was judged at high risk of bias for either outcome. All results were judged at low risk for deviations from intended interventions and measurement of the outcome. Some concerns arose primarily from the randomization process (four of 11 for both outcomes) and selection of the reported result (five of 11 for ALT and three of 11 for HbA1c), while missing outcome data contributed to some concerns in one result for each outcome. Open-label design alone did not warrant an elevated risk-of-bias judgment because no relevant deviations arising from the trial context were identified, and both ALT and HbA1c were objective laboratory outcomes unlikely to be materially influenced by knowledge of intervention assignment.

### 3.3 Liver enzymes

**Figure 4:**
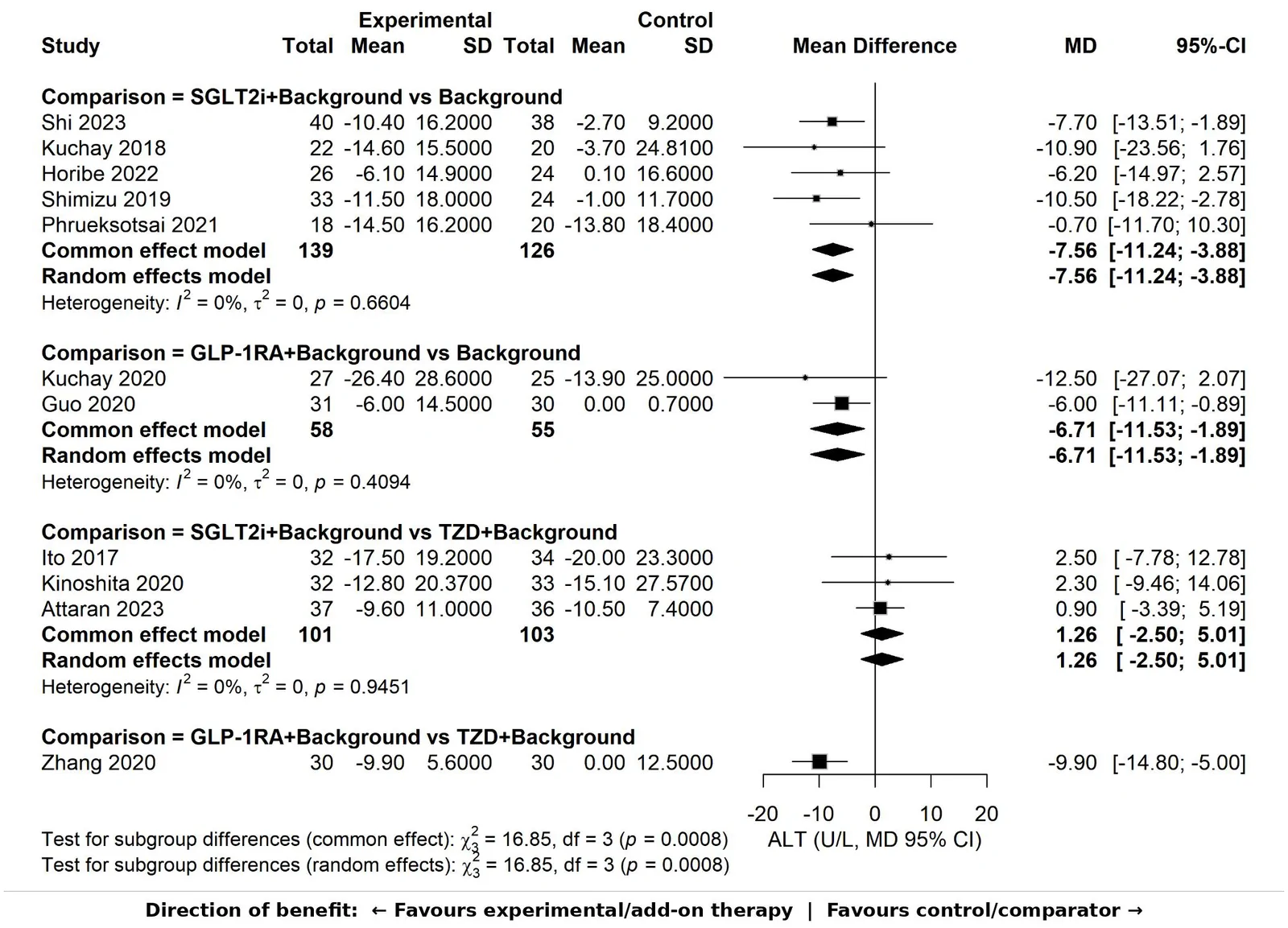
Forest plot of the effects of glucose-lowering therapies on alanine aminotransferase (ALT). Results are presented as mean differences (MDs) with 95% confidence intervals. Heterogeneity is reported using *I*^2^ and τ^2^. Values to the left of the null line favor the experimental/add-on therapy, whereas values to the right favor the control/comparator. The comparison categories represent distinct pairwise treatment contrasts rather than subgroups; the software-generated test labeled “test for subgroup differences” was not used for between-treatment inference. The “total” columns show the numbers of participants included in the outcome analysis for each study arm; these denominators may differ from the numbers randomized or treated.

**Figure 5:**
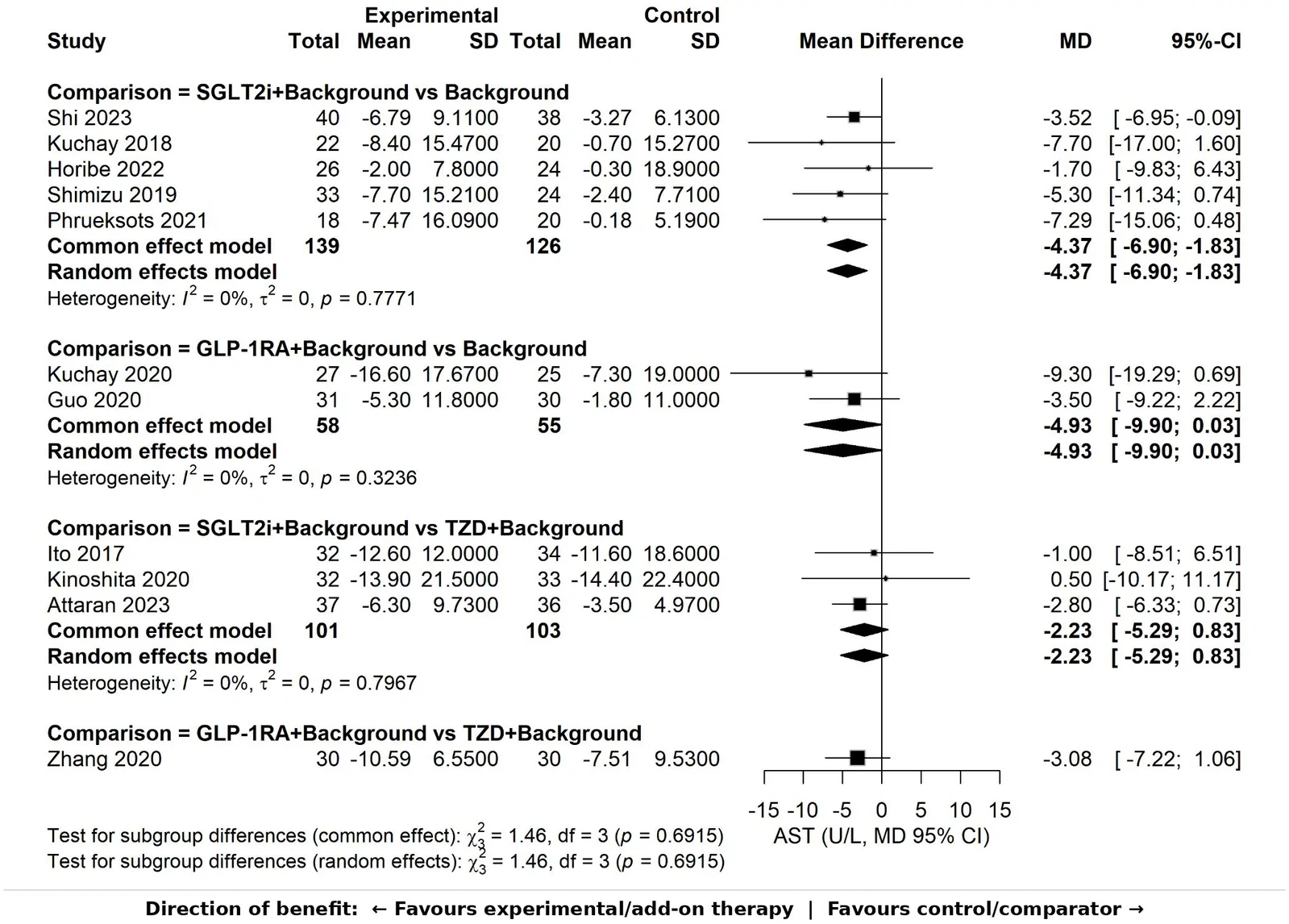
Forest plot of the effects of glucose-lowering therapies on aspartate aminotransferase (AST). Results are presented as mean differences (MDs) with 95% confidence intervals. Heterogeneity is reported using I^2^ and τ^2^. Values to the left of the null line favor the experimental/add-on therapy, whereas values to the right favor the control/comparator. The comparison categories represent distinct pairwise treatment contrasts rather than subgroups; the software-generated test labeled “test for subgroup differences” was not used for between-treatment inference. The “total” columns show the numbers of participants included in the outcome analysis for each study arm; these denominators may differ from the numbers randomized or treated.

Eleven studies reported changes in ALT and AST. In five trials with 265 analyzed participants, an SGLT2 inhibitor was added to stable background therapy and compared with continuation of the same background treatment. For the coprimary ALT outcome, the pooled estimate favored SGLT2 inhibitors (MD, − 7.56 U/L; 95% CI, − 11.24 to − 3.88; *p* < 0.001; *I*^2^ = 0%; **Figure 4**). The exploratory AST estimate also favored SGLT2 inhibitors (MD, − 4.37 U/L; 95% CI, − 6.90 to − 1.83; *p* = 0.001; *I*^2^ = 0%; **Figure 5**).

Two trials with 113 analyzed participants compared GLP-1RAs with matched background therapy. The pooled estimate favored GLP-1RAs for ALT (MD, − 6.71 U/L; 95% CI, − 11.53 to − 1.89; *p* = 0.006), whereas the AST estimate was imprecise and included the null (MD, − 4.93 U/L; 95% CI, − 9.90 to 0.03; *p* = 0.052). Direct comparisons between SGLT2 inhibitors and pioglitazone did not show statistically clear differences in ALT or AST; these findings should not be interpreted as evidence of equivalent treatment effects. One trial directly compared a GLP-1RA with pioglitazone and favored the GLP-1RA for ALT but not AST. The comparison categories were distinct pairwise treatment contrasts rather than subgroups, and the software-generated “test for subgroup differences” was not used for between-treatment inference. These estimates represent incremental effects relative to matched background management rather than standalone drug efficacy. Leave-one-out analyses yielded similar pooled estimates. As few studies contributed to most comparisons, publication bias could not be reliably assessed or excluded.

### 3.4 Hepatic fat and liver stiffness

**Figure 6:**
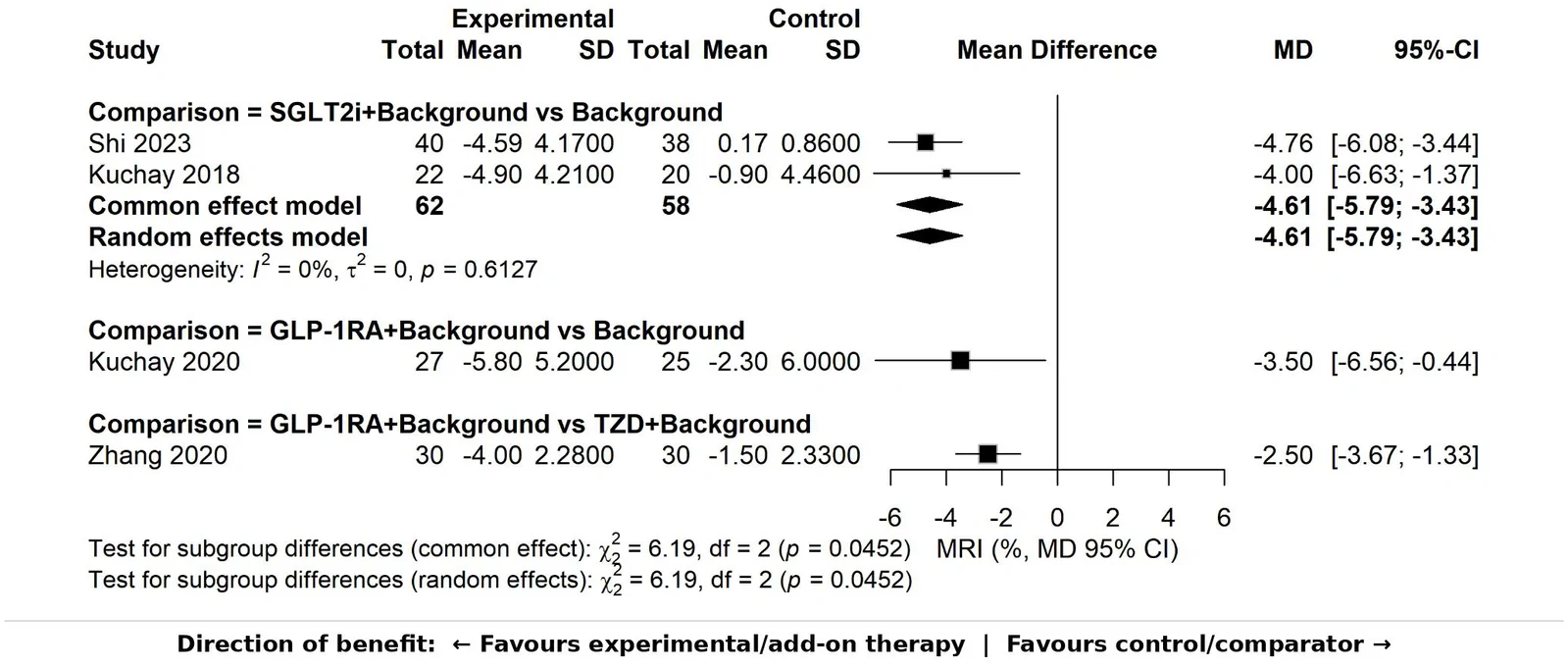
Forest plot of the effects of glucose-lowering therapies on magnetic resonance imaging–proton density fat fraction (MRI-PDFF). Results are presented as mean differences (MDs) with 95% confidence intervals. Heterogeneity is reported using I^2^ and τ^2^. Values to the left of the null line favor the experimental/add-on therapy, whereas values to the right favor the control/comparator. The comparison categories represent distinct pairwise treatment contrasts rather than subgroups; the software-generated test labeled “test for subgroup differences” was not used for between-treatment inference. The “total” columns show the numbers of participants included in the outcome analysis for each study arm; these denominators may differ from the numbers randomized or treated.

Four RCTs with 232 analyzed participants showed change in MRI-PDFF (**Figure 6**). Two trials involving 120 analyzed participants had a pooled effect favoring addition of an SGLT2 inhibitor on top of background therapy (MD, − 4.61 percentage points; 95% CI, − 5.79 to − 3.43; *p* < 0.001; *I*^2^ = 0%). Single-trial estimates favored a GLP-1RA versus background therapy (MD, − 3.50 percentage points; 95% CI, − 6.56 to − 0.44; *p* = 0.025), as well as a GLP-1RA versus thiazolidinedione (MD, − 2.50 percentage points; 95% CI, − 3.67 to − 1.33; *p* < 0.001); no eligible trial directly compared SGLT2 inhibitors with thiazolidinediones regarding MRI-PDFF.

**Figure 7:**
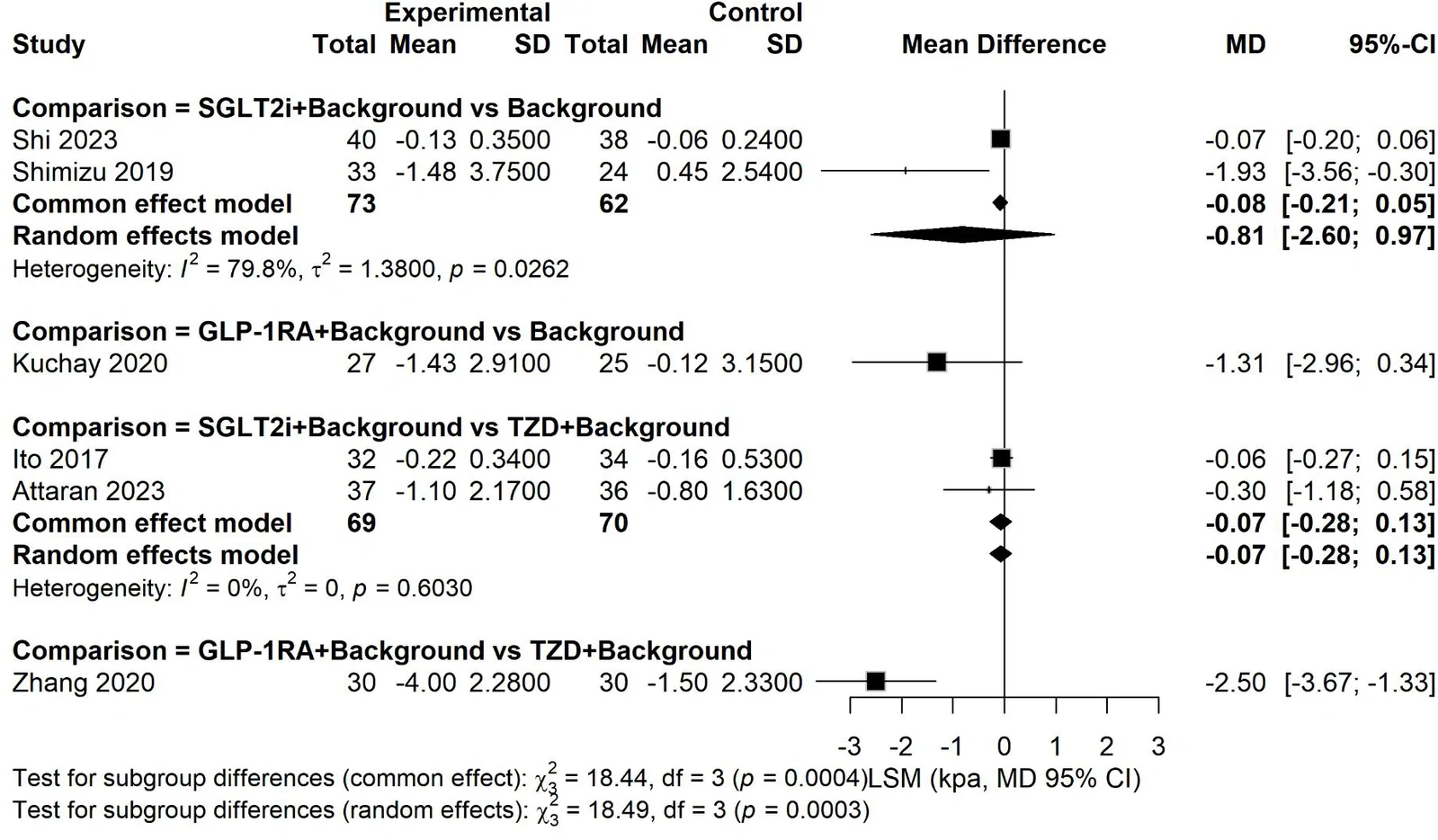
Forest plot of the effects of glucose-lowering therapies on liver stiffness measurement (LSM). Results are presented as mean differences (MDs) with 95% confidence intervals. Heterogeneity is reported using I^2^ and τ^2^. Values to the left of the null line favor the experimental/add-on therapy, whereas values to the right favor the control/comparator. The comparison categories represent distinct pairwise treatment contrasts rather than subgroups; the software-generated test labeled “test for subgroup differences” was not used for between-treatment inference. The “total” columns show the numbers of participants included in the outcome analysis for each study arm; these denominators may differ from the numbers randomized or treated.

Six RCTs with 386 analyzed participants evaluated liver stiffness measurement (LSM) (**Figure 7**). Neither SGLT2 inhibitors nor GLP-1RAs showed a consistent reduction in LSM versus background therapy; the SGLT2 inhibitor estimate was heterogeneous (*I*^2^ = 79.8%) and not robust to sensitivity analysis. Direct comparisons between SGLT2 inhibitors and thiazolidinediones did not show a statistically clear difference, whereas one trial favored a GLP-1RA over a thiazolidinedione. Given the small number of studies, these findings do not establish equivalence between treatments.

### 3.5 Glycemic outcomes

**Figure 8:**
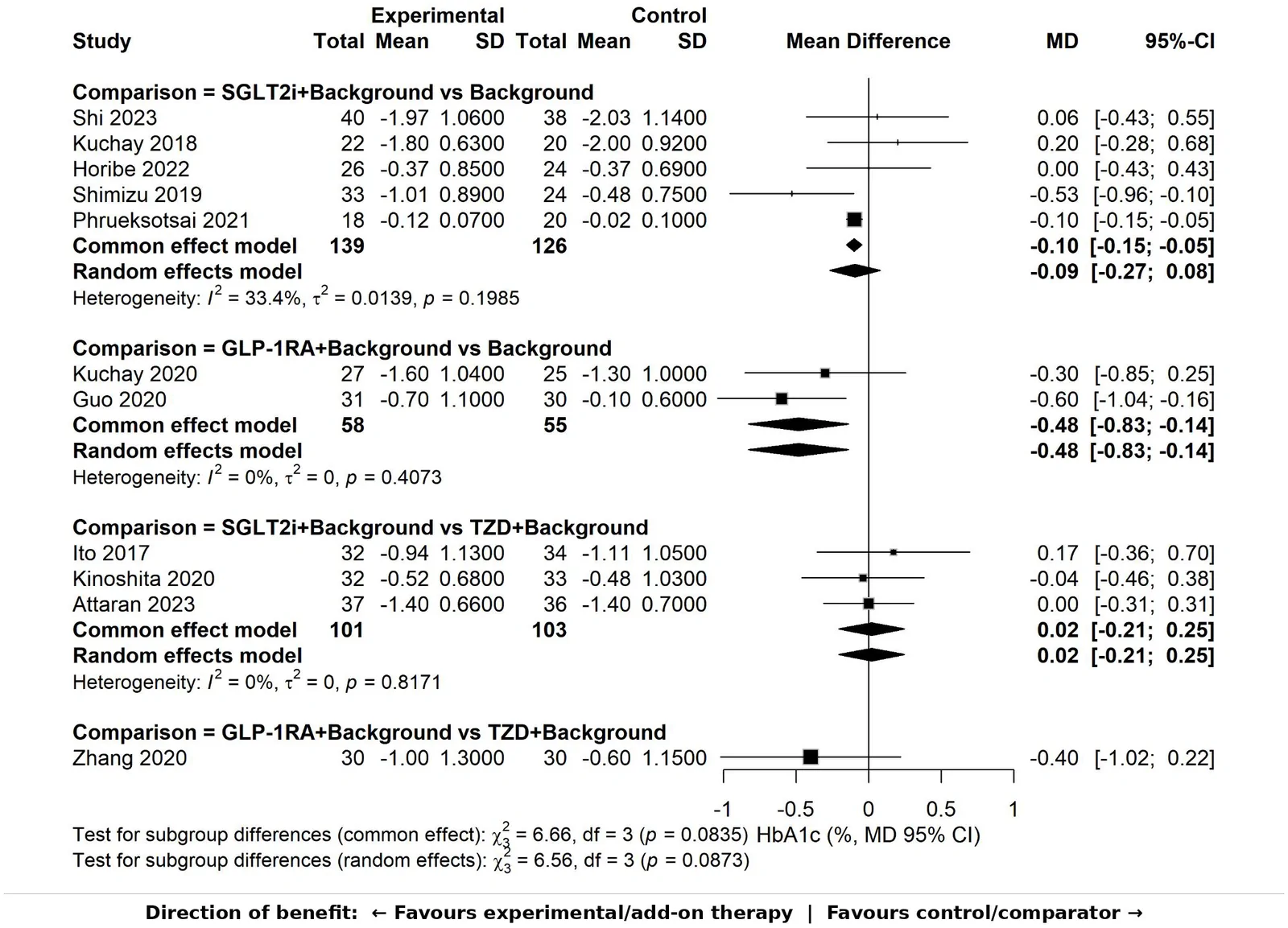
Forest plot of the effects of glucose-lowering therapies on glycated hemoglobin (HbA1c). Results are presented as mean differences (MDs) with 95% confidence intervals. Heterogeneity is reported using *I*^2^ and τ^2^. Values to the left of the null line favor the experimental/add-on therapy, whereas values to the right favor the control/comparator. The comparison categories represent distinct pairwise treatment contrasts rather than subgroups; the software-generated test labeled “test for subgroup differences” was not used for between-treatment inference. The “total” columns show the numbers of participants included in the outcome analysis for each study arm; these denominators may differ from the numbers randomized or treated.

**Figure 9:**
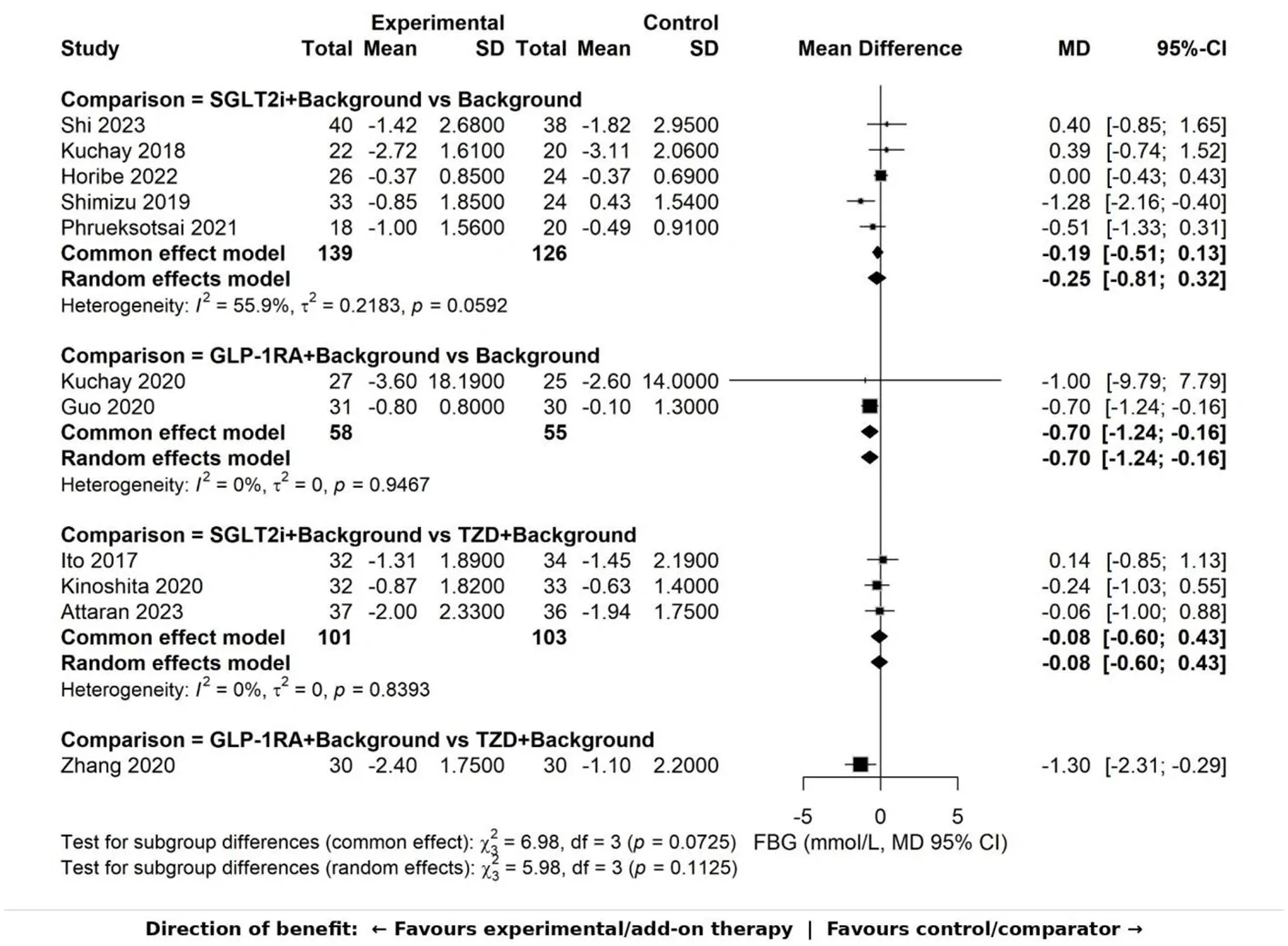
Forest plot of the effects of glucose-lowering therapies on fasting plasma glucose (FPG). Results are presented as mean differences (MDs) with 95% confidence intervals. Heterogeneity is reported using I^2^ and τ^2^. Values to the left of the null line favor the experimental/add-on therapy, whereas values to the right favor the control/comparator. The comparison categories represent distinct pairwise treatment contrasts rather than subgroups; the software-generated test labeled “test for subgroup differences” was not used for between-treatment inference. The “total” columns show the numbers of participants included in the outcome analysis for each study arm; these denominators may differ from the numbers randomized or treated.

Across the 11 randomized trials, 642 analyzed participants contributed data on glycated hemoglobin (HbA1c; **Figure 8**) and fasting plasma glucose (**Figure 9**). Compared with matched background therapy, SGLT2 inhibitors showed little or no additional effect on HbA1c (MD, − 0.09%; 95% CI, − 0.27 to 0.08; *p* = 0.198) and an imprecise effect on fasting plasma glucose (MD, − 0.25 mmol/L; 95% CI, − 0.81 to 0.32; *p* = 0.386). Leave-one-out analyses yielded similar estimates.

Compared with matched background therapy, GLP-1RAs reduced HbA1c (MD, − 0.48%; 95% CI, − 0.83 to − 0.14; *p* = 0.006) and fasting plasma glucose (MD, − 0.70 mmol/L; 95% CI, − 1.24 to − 0.16; *p* = 0.011), with no substantial heterogeneity. Direct comparisons between SGLT2 inhibitors and thiazolidinediones did not show statistically clear differences in either outcome. In one trial, a GLP-1RA favored lower fasting plasma glucose versus a thiazolidinedione, whereas the HbA1c estimate included the null. No formal interaction tests for clinical or methodological subgroups were performed; therefore, no subgroup inferences were made.

### 3.6 Lipid outcomes

**Figure 10:**
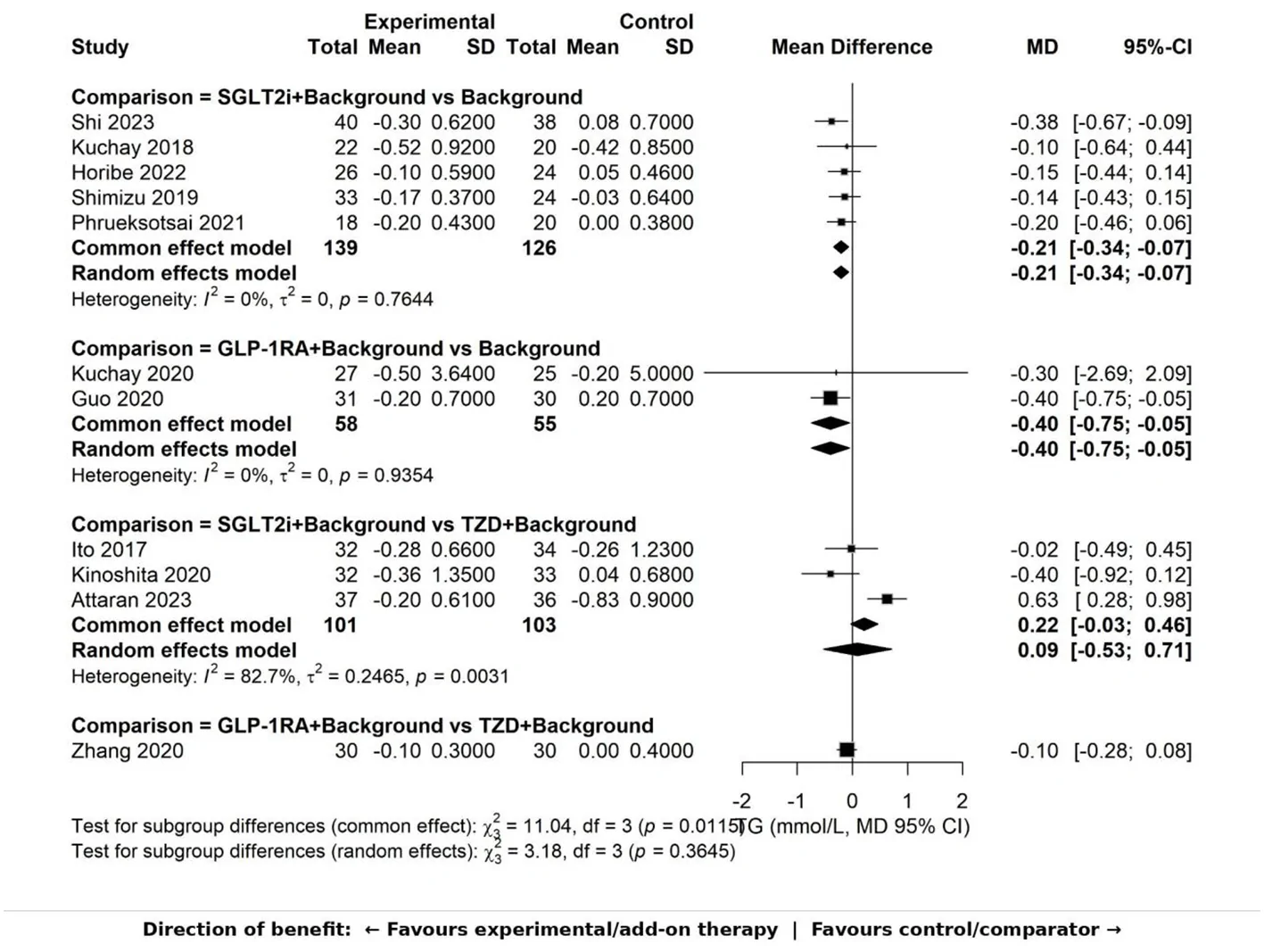
Forest plot of the effects of glucose-lowering therapies on triglycerides. Results are presented as mean differences (MDs) with 95% confidence intervals. Heterogeneity is reported using I^2^ and τ^2^. Values to the left of the null line favor the experimental/add-on therapy, whereas values to the right favor the control/comparator. The comparison categories represent distinct pairwise treatment contrasts rather than subgroups; the software-generated test labeled “test for subgroup differences” was not used for between-treatment inference. The “total” columns show the numbers of participants included in the outcome analysis for each study arm; these denominators may differ from the numbers randomized or treated.

For triglycerides, exploratory pooled estimates favored SGLT2 inhibitors and GLP-1RAs over background therapy by − 0.21 and − 0.40 mmol/L, respectively, with little observed heterogeneity (**Figure 10**). Direct active-treatment comparisons did not show statistically clear differences. The SGLT2 inhibitor-versus-thiazolidinedione comparison was heterogeneous and sensitive to individual studies; therefore, this secondary finding should be interpreted cautiously.

**Figure 11:**
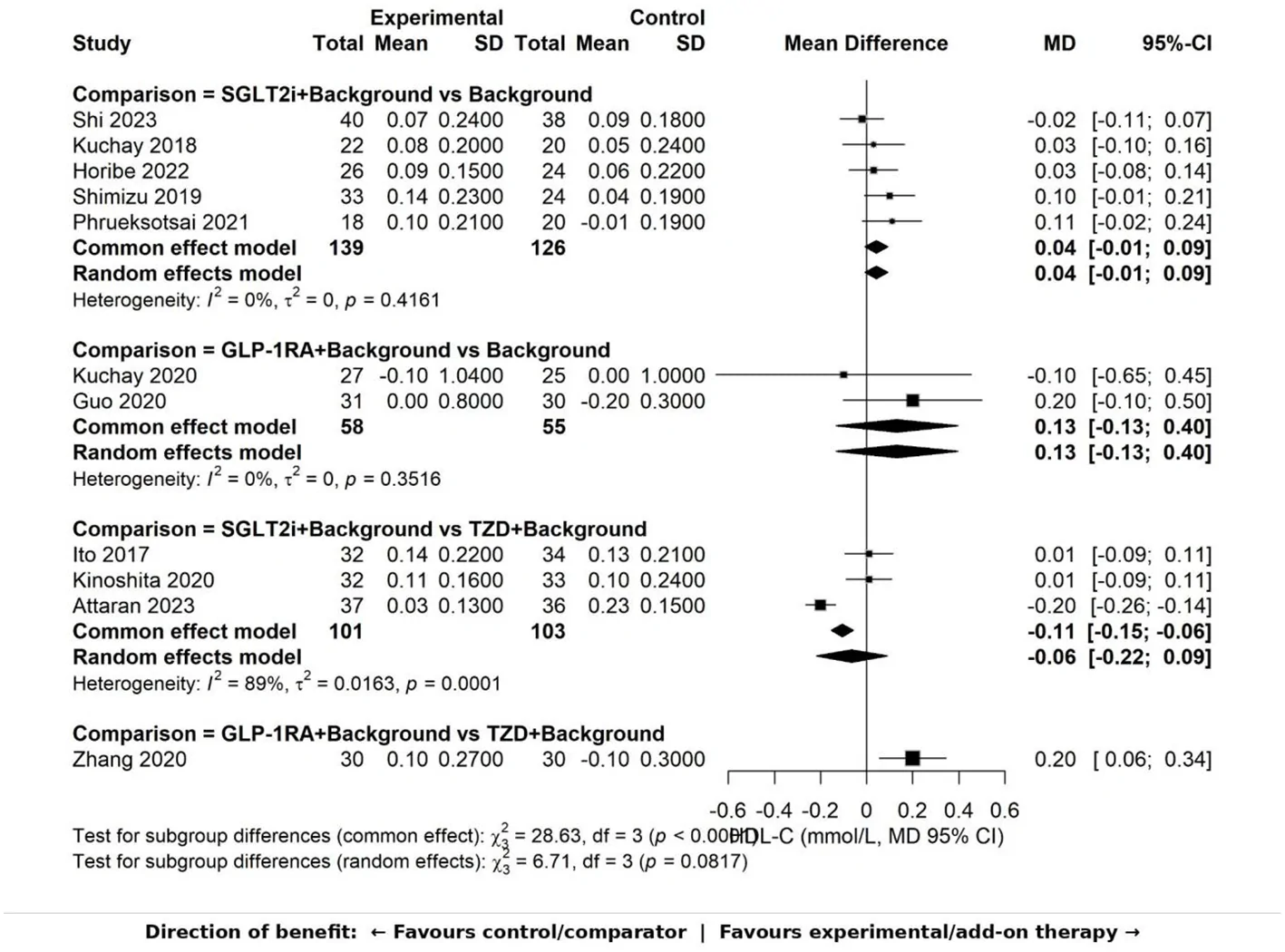
Forest plot of the effects of glucose-lowering therapies on high-density lipoprotein cholesterol (HDL-C). Results are presented as mean differences (MDs) with 95% confidence intervals. Heterogeneity is reported using I^2^ and τ^2^. Values to the right of the null line favor the experimental/add-on therapy, whereas values to the left favor the control/comparator. The comparison categories represent distinct pairwise treatment contrasts rather than subgroups; the software-generated test labeled “test for subgroup differences” was not used for between-treatment inference. The “total” columns show the numbers of participants included in the outcome analysis for each study arm; these denominators may differ from the numbers randomized or treated.

For high-density lipoprotein cholesterol, exploratory estimates did not show statistically clear effects of SGLT2 inhibitors or GLP-1RAs versus background therapy (**Figure 11**), nor of SGLT2 inhibitors versus thiazolidinediones. Several pooled estimates were heterogeneous or unstable. A single trial favored a GLP-1RA over a thiazolidinedione; this isolated finding should be interpreted cautiously.

**Figure 12:**
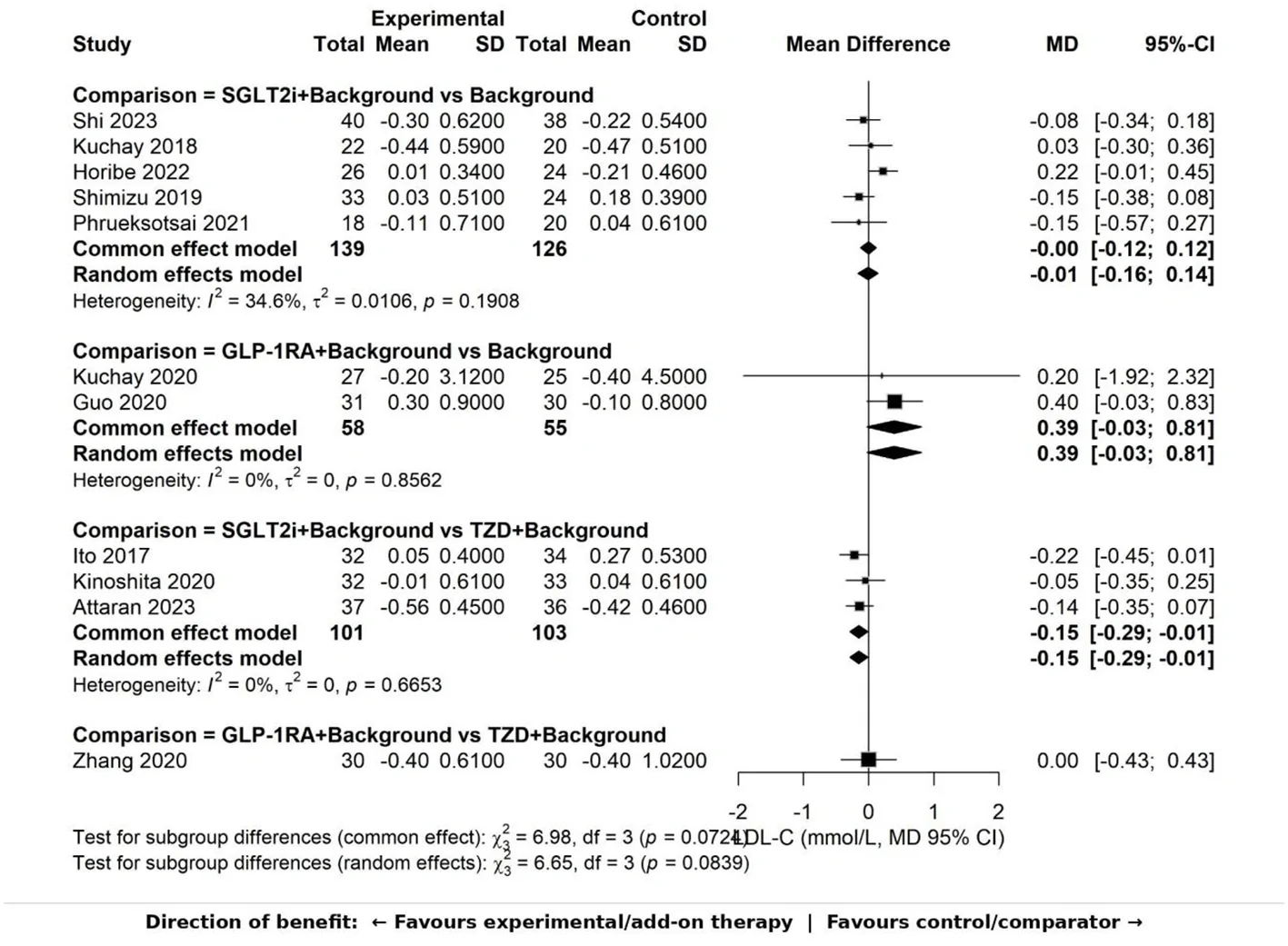
Forest plot of the effects of glucose-lowering therapies on low-density lipoprotein cholesterol (LDL-C). Results are presented as mean differences (MDs) with 95% confidence intervals. Heterogeneity is reported using I^2^ and τ^2^. Values to the left of the null line favor the experimental/add-on therapy, whereas values to the right favor the control/comparator. The comparison categories represent distinct pairwise treatment contrasts rather than subgroups; the software-generated test labeled “test for subgroup differences” was not used for between-treatment inference. The “total” columns show the numbers of participants included in the outcome analysis for each study arm; these denominators may differ from the numbers randomized or treated.

For low-density lipoprotein cholesterol, exploratory estimates did not show statistically clear differences for either drug class versus background therapy (**Figure 12**). The SGLT2 inhibitor-versus-thiazolidinedione comparison nominally favored SGLT2 inhibitors (MD, − 0.15 mmol/L; 95% CI, − 0.29 to − 0.01; *p* = 0.035), whereas the GLP-1RA-versus-thiazolidinedione comparison did not show a statistically clear difference. As these were secondary analyses without multiplicity adjustment, the findings should be considered hypothesis-generating.

### 3.7 Other metabolic outcomes

**Figure 13:**
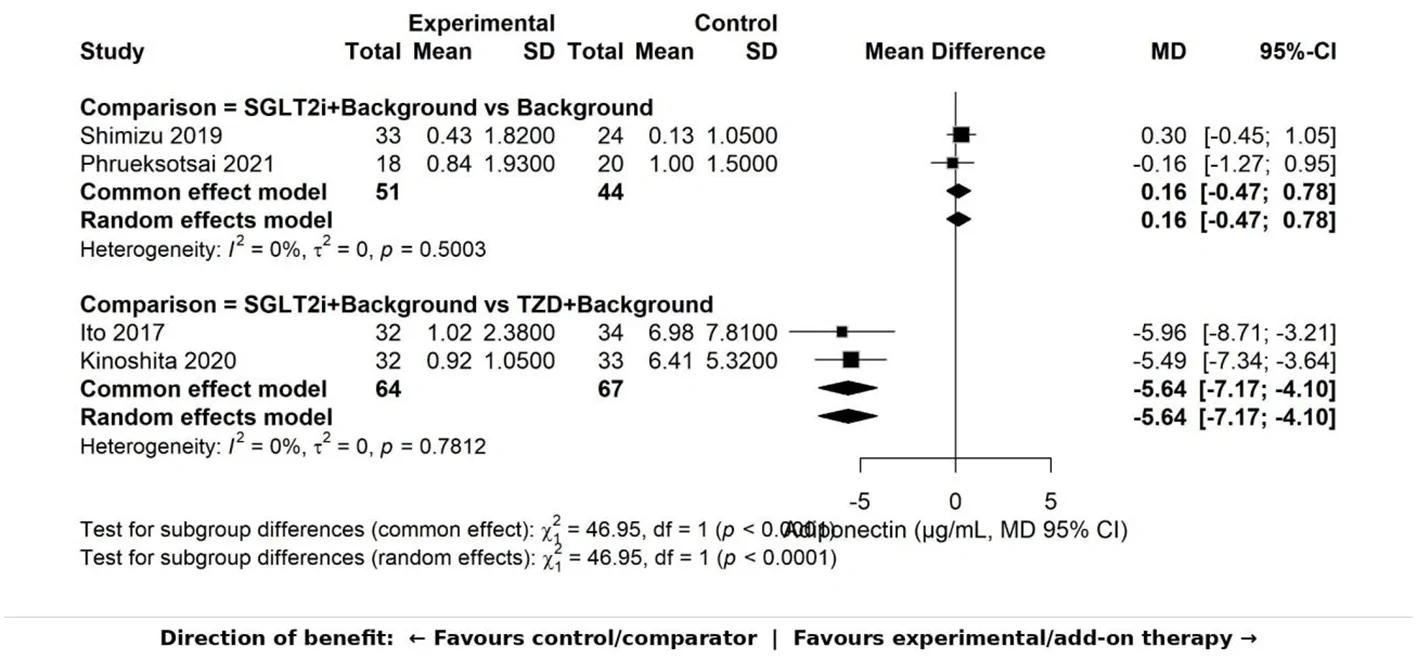
Forest plot of the effects of glucose-lowering therapies on serum adiponectin. Results are presented as mean differences (MDs) with 95% confidence intervals. Heterogeneity is reported using I^2^ and τ^2^. Values to the right of the null line favor the experimental/add-on therapy, whereas values to the left favor the control/comparator. The comparison categories represent distinct pairwise treatment contrasts rather than subgroups; the software-generated test labeled “test for subgroup differences” was not used for between-treatment inference. The “total” columns show the numbers of participants included in the outcome analysis for each study arm; these denominators may differ from the numbers randomized or treated.

Four trials with 226 analyzed participants reported adiponectin (**Figure 13**). The exploratory estimate did not show a statistically clear difference between SGLT2 inhibitors and background therapy. Thiazolidinediones were associated with higher adiponectin than SGLT2 inhibitors (SGLT2 inhibitor minus thiazolidinedione MD, − 5.64 μg/mL; 95% CI, − 7.17 to − 4.10; *p* < 0.001). No eligible trial specifically reported a GLP-1RA-versus-background comparison for this outcome.

**Figure 14:**
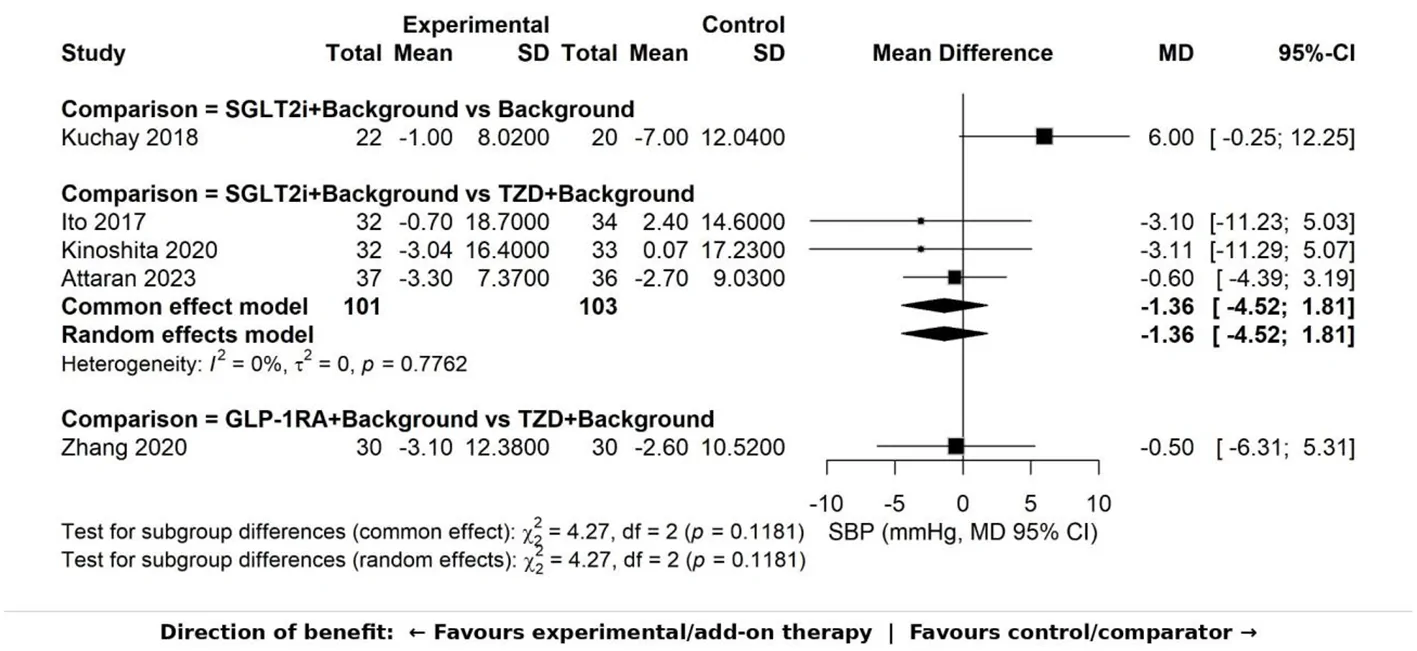
Forest plot of the effects of glucose-lowering therapies on systolic blood pressure (SBP). Results are presented as mean differences (MDs) with 95% confidence intervals. Heterogeneity is reported using I^2^ and τ^2^. Values to the left of the null line favor the experimental/add-on therapy, whereas values to the right favor the control/comparator. The comparison categories represent distinct pairwise treatment contrasts rather than subgroups; the software-generated test labeled “test for subgroup differences” was not used for between-treatment inference. The “total” columns show the numbers of participants included in the outcome analysis for each study arm; these denominators may differ from the numbers randomized or treated.

Five trials with 306 analyzed participants reported systolic blood pressure (**Figure 14**). Exploratory estimates did not show statistically clear differences for SGLT2 inhibitors versus background therapy, SGLT2 inhibitors versus thiazolidinediones, or GLP-1RAs versus thiazolidinediones. No included trial directly compared a GLP-1RA with background therapy for this outcome.

### 3.8 Sensitivity analysis and publication bias

Sensitivity analyses and explorations of small-study effects are reported in the **Supplementary Appendix**. Estimates for the coprimary outcomes, ALT and HbA1c, were generally similar in leave-one-out analyses, as were several secondary estimates, including AST, fasting plasma glucose, triglycerides, and low-density lipoprotein cholesterol. As secondary analyses were exploratory and were not adjusted for multiple comparisons, nominally statistically significant secondary findings should be considered hypothesis-generating. Results based on few studies, substantial heterogeneity, or unstable sensitivity analyses should be interpreted cautiously. Most meta-analyses included fewer than 10 studies; therefore, funnel plots were exploratory and could not reliably exclude publication bias.

### 3.9 Safety outcomes

Safety outcomes were inconsistently reported across all 11 trials (**Supplementary Table 9**). Reported adverse events included gastrointestinal reactions, hypoglycemia, urinary or genital infections, edema, skin reactions, and isolated serious or severe events, including heart failure. Discontinuations due to adverse events were reported in participants receiving empagliflozin, dapagliflozin, dulaglutide, and pioglitazone. Some studies stated only that no significant or severe adverse events occurred, whereas others did not provide arm-level event counts. As definitions and reporting formats differed and safety data were incomplete, quantitative pooling was not considered appropriate. Comparative safety therefore remains uncertain.

## 4 Discussion

This review included 11 randomized controlled trials conducted in five Asian countries, with 692 participants randomized to eligible comparison arms and 642 contributing to the coprimary efficacy analyses (14, 15, 19, 28–35). Compared with continuation of matched background therapy, add-on SGLT2 inhibitors reduced ALT but showed little or no additional effect on HbA1c, whereas add-on GLP-1RAs reduced both ALT and HbA1c (15, 19, 30–32, 34, 35). Exploratory secondary outcomes suggested changes in several hepatic and metabolic markers, including AST, MRI-PDFF, triglycerides, fasting plasma glucose, and BMI, but the amount and robustness of evidence varied by outcome and treatment class. Direct comparisons between SGLT2 inhibitors and pioglitazone did not show clear differences in liver enzymes or glycemic outcomes, and most GLP-1RA-versus-pioglitazone evidence came from a single trial. No eligible trial directly compared an SGLT2 inhibitor with a GLP-1RA (14, 28, 29, 33). Accordingly, the available evidence does not support a reliable treatment ranking or an overall superior drug class. Outcome-specific GRADE certainty was moderate for both ALT and HbA1c in the SGLT2 inhibitor-versus-background comparison; moderate for ALT and low for HbA1c in the GLP-1RA-versus-background comparison; high for both coprimary outcomes in the SGLT2 inhibitor-versus-pioglitazone comparison, supporting little or no clinically important between-treatment difference rather than formal equivalence; and moderate for ALT and low for HbA1c in the single-trial GLP-1RA-versus-pioglitazone comparison. These ratings reflect confidence in estimated magnitudes relative to the stated thresholds and do not establish histological benefit or a treatment-class hierarchy (26, 27).

The hepatic findings primarily reflect short-term changes in biochemical markers and hepatic steatosis rather than demonstrated modification of liver disease (27, 36). Compared with continued matched background therapy, SGLT2 inhibitors reduced ALT, AST, and MRI-PDFF, broadly consistent with prior evidence for aminotransferase and hepatic fat outcomes (37). Neither SGLT2 inhibitors nor GLP-1RAs showed consistent improvement in liver stiffness in this analysis, and the SGLT2 inhibitor estimate was highly heterogeneous. Differences between aminotransferase or steatosis outcomes and liver stiffness may partly reflect their different biological time courses; follow-up of 12–28 weeks may be too short to detect meaningful fibrosis-related tissue remodeling (27). Importantly, aminotransferases mainly reflect hepatocellular injury and MRI-PDFF quantifies hepatic fat content; improvements in these surrogates do not by themselves establish metabolic dysfunction-associated steatohepatitis (MASH) resolution, fibrosis regression, or reductions in liver-related clinical events (27, 36).

Direct active-comparator evidence requires similarly cautious interpretation. Head-to-head trials of SGLT2 inhibitors and pioglitazone did not show statistically clear differences in ALT or AST (14, 28, 29), but the small number of trials, modest sample sizes, and short follow-up limit the precision and scope of these comparisons. The GRADE assessment provided high certainty that the observed ALT and HbA1c differences were below the prespecified clinically important thresholds; however, these superiority trials were not designed to establish equivalence or noninferiority. A nonsignificant between-treatment difference should therefore not be interpreted as proof of therapeutic equivalence. Conversely, the available studies also do not establish superiority of SGLT2 inhibitors over pioglitazone for liver-specific efficacy. Demonstrating equivalence or noninferiority requires an appropriate design and a prespecified margin (38).

The magnitude of the observed effects also warrants cautious clinical interpretation. The pooled ALT reductions of 7.56 U/L with SGLT2 inhibitors and 6.71 U/L with GLP-1RAs, together with the 4.37 U/L AST reduction with SGLT2 inhibitors, represent modest biochemical changes and should not be interpreted as evidence of histological benefit (27). The 4.61-percentage-point absolute reduction in MRI-PDFF with SGLT2 inhibitors supports reduced hepatic fat content. A ≥ 30% within-participant relative decline in MRI-PDFF has been used as a treatment-response threshold in MASH/NASH studies and is associated with a greater likelihood of histological response (36); however, the absolute mean difference estimated here cannot determine how many participants achieved that threshold. The 0.48-percentage-point HbA1c reduction with GLP-1RAs may be relevant to glycemic management depending on baseline HbA1c and individualized treatment goals, but the estimate was derived from only two small trials (19, 31, 39). Its 95% CI (− 0.83 to − 0.14) crossed the 0.5-percentage-point threshold used in the GRADE imprecision assessment, and the accrued information (*n* = 113) was marginally below the calculated OIS (114 participants). By contrast, the estimated 0.09-percentage-point HbA1c reduction with SGLT2 inhibitors was small; its 95% CI (− 0.27 to 0.08) remained within the ± 0.5-percentage-point threshold, and the calculated OIS was met (265 accrued vs. 91 required). Changes in BMI were also modest, and the SGLT2 inhibitor estimate was heterogeneous and sensitive to individual studies. Overall, statistical evidence of change in a surrogate outcome should not be equated with clinically important long-term benefit (27, 36).

The glycemic and broader metabolic findings suggest different short-term response patterns rather than a treatment hierarchy. Compared with continuation of matched background therapy, SGLT2 inhibitors did not demonstrate a clear additional reduction in HbA1c or fasting plasma glucose, whereas GLP-1RAs reduced both outcomes (15, 19, 30–32, 34, 35). The small HbA1c estimate for SGLT2 inhibitors may partly reflect baseline glycemic status because larger absolute reductions are generally observed at higher baseline HbA1c levels (40); however, this explanation remains speculative because the available study-level data did not permit reliable meta-regression by baseline HbA1c. The background-controlled evidence for GLP-1RAs was derived from only two small trials, and no direct SGLT2 inhibitor-versus-GLP-1RA comparison was available (14, 15, 19, 28–35). Differences between separate background-controlled analyses therefore should not be interpreted as evidence of between-class superiority (17, 18, 41). Likewise, observed changes in triglycerides, low-density lipoprotein cholesterol, and BMI should remain exploratory because secondary outcomes were not adjusted for multiplicity and several estimates were based on sparse or heterogeneous evidence.

The available data were insufficient for reliable comparison of safety across treatment classes. Reported adverse events included gastrointestinal reactions, hypoglycemia, urinary or genital infections, skin reactions, edema, and isolated serious or severe events; discontinuations due to adverse events were also reported in several treatment groups (14, 15, 19, 28–35). Adverse-event definitions and reporting varied substantially across trials, and some studies did not provide arm-level event counts or reported only that no significant or severe events occurred. Quantitative pooling was therefore not appropriate. Comparative safety could not be determined from this dataset, and the absence of a reported event should not be interpreted as evidence that the event did not occur (42).

Potential mechanisms provide biological context but were not tested in this meta-analysis. For GLP-1RAs, reductions in body weight and visceral adiposity, together with improved glycemic control and insulin sensitivity, could reduce hepatic lipid accumulation and lipotoxic stress (43, 44). One randomized weight-matched study suggested that some metabolic effects of liraglutide may be partly independent of weight loss (44), whereas experimental work in primary human hepatocytes and hepatic stellate cells did not show clear direct cellular effects of liraglutide, arguing against assuming a direct hepatocyte-mediated mechanism (45). Glycosuria and negative energy balance induced by SGLT2 inhibitors may contribute to modest weight loss and reductions in ectopic fat (46). PPAR-γ-mediated changes in adipose tissue, improved insulin sensitivity, and increased adiponectin provide a plausible explanation for the higher adiponectin observed with pioglitazone than with SGLT2 inhibitors in the current analysis (47). These mechanistic considerations support biological plausibility but do not establish comparative hepatic superiority among agents.

The restriction to Asian study settings also requires careful interpretation. Although T2DM and MASLD may occur at relatively low BMI in some Asian populations, BMI does not fully capture visceral or metabolically active adiposity (48–50), and the available data do not establish ethnicity-specific pharmacological responses. Study-level analyses have reported inconsistent findings regarding ethnic differences in the glycemic efficacy of SGLT2 inhibitors (51, 52), and such aggregate comparisons are susceptible to ecological bias (53). Eligibility in this review was based on study location rather than participant ethnicity; included trials were conducted in China, Japan, India, Iran, and Thailand, settings that differ in body composition, diet, diabetes phenotype, background treatment, and clinical practice (14, 15, 19, 28–35). Country-specific analyses were not feasible because few trials were available from each setting. The pooled estimates therefore describe the Asian study settings represented in this review and should not be interpreted as demonstrating a uniform response across all Asian populations or a differential response relative to non-Asian populations.

Clinically, these findings are more informative for individualized treatment considerations than for class ranking. Treatment selection should consider liver disease severity, glycemic and weight-management goals, cardiovascular and renal comorbidities, route of administration, tolerability, safety, access, and patient preference (9–12, 39). The established cardiovascular and kidney benefits of SGLT2 inhibitors derive largely from studies not represented in the liver-specific trials summarized here and should be considered independently of the hepatic estimates in this review (54). Pioglitazone may be considered for glycemic management in selected patients, but potential benefits should be balanced against risks including weight gain, edema, and heart failure (55, 56). More recent evidence for GLP-1RAs with MASH benefit has been incorporated into current guidance, while pioglitazone remains an option for selected patients with T2DM and MASH; these recommendations draw on a broader evidence base than the trials included in the present meta-analysis (39, 57).

More recent evidence for semaglutide should be considered separately from the class-level estimates presented here. In the phase 3 ESSENCE trial, semaglutide 2.4 mg improved both MASH resolution without worsening of fibrosis and fibrosis improvement without worsening of MASH after 72 weeks in adults with biopsy-confirmed MASH and F2–F3 fibrosis (58). Semaglutide subsequently received regulatory authorization for selected patients with MASH and moderate-to-advanced fibrosis in major jurisdictions (57, 59). As semaglutide was not represented in the eligible trials in this review, these agent-specific histological data should not be extrapolated to the GLP-1RA class or used to reinterpret the short-term class-level estimates from the present meta-analysis (57, 58).

Adequately powered, long-term head-to-head randomized trials—particularly comparisons of SGLT2 inhibitors with GLP-1RAs—are needed with standardized or protocol-defined background glucose-lowering management (17, 18, 41). Future trials should include histological outcomes or validated fibrosis-related endpoints, prespecified outcome hierarchies and multiplicity strategies, standardized adverse-event reporting, and follow-up sufficient to assess durability and patient-important liver outcomes (27, 42). Studies across diverse Asian settings should also collect participant-level information on ethnicity, body composition and visceral adiposity, baseline glycemic control, and fibrosis stage to evaluate clinically meaningful treatment-effect heterogeneity rather than relying on country-level ecological comparisons (5, 48–50, 53).

### 4.1 Strengths and limitations

A key strength of this meta-analysis was the use of eligibility criteria designed to preserve comparability of background glucose-lowering management between randomized groups. As the review focused on incremental effects, the critical consideration was whether concomitant therapy remained stable or changed only according to a prespecified treatment protocol, rather than whether every individual background-drug dose was reported. Specific background-drug doses were extracted when explicitly available and otherwise recorded as NR. Most trials maintained background therapy, while several permitted protocol-specified adjustments detailed in **Supplementary Table 8A**. Accordingly, the estimates are best interpreted as incremental effects of randomized treatment strategies on otherwise comparable background management, not as pharmacologically isolated effects under identical fixed doses in every participant. Several limitations should be considered. First, only 11 RCTs were included, with 692 participants randomized to eligible comparison arms and 642 contributing to the coprimary efficacy analyses. Many comparisons, particularly those involving GLP-1RAs, were informed by only one or two trials, limiting precision and precluding robust subgroup or meta-regression analyses; estimates of between-study heterogeneity are also uncertain when few studies contribute to a synthesis. Second, follow-up ranged from 12 to 28 weeks, limiting inference about durability and long-term clinical outcomes. Third, most liver-related outcomes were biochemical or imaging surrogates, with insufficient data on histological MASH resolution, fibrosis progression or regression, cirrhosis, or liver-related clinical events; improvements in surrogate markers may not translate into long-term hepatic benefit (27, 36). Fourth, several secondary outcomes were heterogeneous or sensitive to individual studies, and no formal multiplicity adjustment was applied. The small number of studies also limited assessment of publication bias. Fifth, safety reporting was inconsistent and incomplete, preventing reliable quantitative comparison among treatment classes (42). Sixth, the planned network meta-analysis could not be interpreted reliably because the available evidence did not meet consistency assumptions; conclusions therefore rely on separate direct pairwise estimates rather than formal comparative rankings. Finally, eligibility was based on Asian study location rather than participant ethnicity, limiting generalizability across heterogeneous Asian populations and beyond the represented settings (5, 48–50, 53). The final pairwise analysis was also retrospectively registered after the analytical strategy changed from the initially planned network meta-analysis; this protocol change should be considered when interpreting the findings (60).

## 5 Conclusion

Among adults with T2DM and MASLD enrolled in randomized trials conducted in Asian study settings, adding an SGLT2 inhibitor or a GLP-1RA to otherwise comparable background glucose-lowering management may provide modest short-term improvements in selected hepatic and metabolic surrogate outcomes. Compared with continuing matched background therapy, SGLT2 inhibitors reduced ALT but showed little or no clinically important additional effect on HbA1c, whereas GLP-1RAs reduced both ALT and HbA1c. Findings for secondary hepatic and metabolic outcomes remain exploratory because of multiplicity, sparse evidence for several endpoints, and heterogeneity or limited robustness of some estimates. Evidence for pioglitazone was derived predominantly from active-comparator trials and was therefore insufficient to reliably estimate its incremental effect compared with continuation of background therapy alone.

The available evidence is insufficient to rank these drug classes or to establish that either SGLT2 inhibitors or GLP-1RAs are superior to pioglitazone for liver-related outcomes. Direct head-to-head evidence was sparse; no eligible trial compared SGLT2 inhibitors with GLP-1RAs, and several comparisons relied on only one or two small trials. Short follow-up, reliance on biochemical and imaging surrogates, and inconsistent and incomplete safety reporting further limit conclusions about durable liver-related clinical benefit and comparative safety. Treatment decisions should therefore remain individualized rather than be based on an inferred class hierarchy from this meta-analysis. Adequately powered, longer-term head-to-head randomized trials with standardized or protocol-defined background therapy, prespecified outcome hierarchies, standardized adverse-event reporting, and clinically meaningful hepatic endpoints—including histological or validated fibrosis-related outcomes—are needed to clarify comparative benefits and risks.

## Data Availability

The original contributions presented in the study are included in the article/**Supplementary Material**. Further inquiries can be directed to the corresponding author.
