# Supplementary material for: Incremental effects of SGLT2 inhibitors, GLP-1 receptor agonists, and pioglitazone in type 2 diabetes and MASLD across Asian study settings: a systematic review and meta-analysis
Source: Front Endocrinol (Lausanne). 2026 Sep 1;17:1942151. doi: 10.3389/fendo.2026.1942151 (PMC13574646; doi:10.3389/fendo.2026.1942151)
Supplement: Supplementary file 1 [file SupplementaryFile1.docx]

Supplementary Appendix

**Incremental Effects of SGLT2 Inhibitors, GLP-1 Receptor Agonists, and Pioglitazone in Type 2 Diabetes and MASLD Across Asian Study Settings: A Systematic Review and Meta-analysis**

# Contents

| **Item** | **Content** |
| --- | --- |
| **Supplementary Table S1** | Full electronic search strategies |
| **Supplementary Table S2** | Detailed RoB 2 assessments for alanine aminotransferase (ALT) |
| **Supplementary Table S3** | Detailed RoB 2 assessments for glycated hemoglobin (HbA1c) |
| **Supplementary Table S4** | GRADE evidence profile: SGLT2 inhibitors plus background therapy vs background therapy |
| **Supplementary Table S5** | GRADE evidence profile: GLP-1 receptor agonists plus background therapy vs background therapy |
| **Supplementary Table S6** | GRADE evidence profile: SGLT2 inhibitors plus background therapy vs TZD plus background therapy |
| **Supplementary Table S7** | GRADE evidence profile: GLP-1 receptor agonists plus background therapy vs TZD plus background therapy |
| **Supplementary Table S8** | Additional characteristics of included randomized controlled trials |
| Supplementary Table S8A | Arm-level treatment, background therapy, and protocol-specified stability/adjustment |
| **Supplementary Table S9** | Adverse events and treatment discontinuation |
| **Supplementary Figures S1-S7** | Exploratory funnel plots and leave-one-out sensitivity analyses |
| **Supplementary Figure S8** | HbA1c-specific risk-of-bias graph |
| **Supplementary Figure S9** | HbA1c-specific study-level risk-of-bias summary |

# Supplementary Table S1. Full electronic search strategies

Databases were searched from inception to June 30, 2026. Search strings are reproduced from the supplied supplementary material.

| **Database** | **Search strategy** |
| --- | --- |
| **PubMed** | ("Glucagon-like peptide-1 receptor agonist*" OR "GLP-1 receptor agonist*" OR "incretin mimetic*" OR albiglutide OR dulaglutide OR exenatide OR liraglutide OR lixisenatide OR semaglutide OR taspoglutide OR "Sodium-Glucose Transporter 2 Inhibitors"[Mesh] OR "sodium-glucose cotransporter-2 inhibitor*" OR "SGLT-2 inhibitor*" OR gliflozin* OR canagliflozin OR dapagliflozin OR empagliflozin OR ertugliflozin OR TZD OR pioglitazone) AND ("Diabetes Mellitus, Type 2"[Mesh] OR "type 2 diabetes" OR T2DM OR "non-insulin dependent diabetes" OR "adult-onset diabetes") AND ("liver enzyme" OR "Non-alcoholic Fatty Liver Disease"[Mesh] OR NAFLD OR "fatty liver" OR "MAFLD" OR "MASLD") AND (Asia OR Asian OR China OR Chinese OR Japan OR Japanese OR Korea OR Korean OR India OR Indian) AND (randomized controlled trial[pt] OR controlled clinical trial[pt] OR randomized[tiab] OR randomised[tiab] OR placebo[tiab]) |
| **Web of Science** | ("Glucagon-like peptide-1 receptor agonist*" OR "GLP-1 receptor agonist*" OR "incretin mimetic*" OR albiglutide OR dulaglutide OR exenatide OR liraglutide OR lixisenatide OR semaglutide OR taspoglutide OR "SGLT-2 inhibitor*" OR "sodium-glucose cotransporter-2 inhibitor*" OR gliflozin* OR canagliflozin OR dapagliflozin OR empagliflozin OR ertugliflozin OR thiazolidinedione* OR TZD OR glitazone* OR pioglitazone OR rosiglitazone) (Topic) and ("type 2 diabetes" OR T2DM OR "non-insulin dependent diabetes") (Topic) and ("liver enzyme" OR NAFLD OR "fatty liver" OR MAFLD OR MASLD) (Topic) and (Asia OR Asian OR China OR Japan OR Korea OR India) (Topic) and (randomized OR randomised OR "controlled trial" OR RCT) (Topic) |
| **Embase** | (("Glucagon-like peptide-1 receptor agonist*" OR "GLP-1 receptor agonist*" OR "incretin mimetic*" OR albiglutide OR dulaglutide OR exenatide OR liraglutide OR lixisenatide OR semaglutide OR "SGLT-2 inhibitor*"OR" sodium-glucose cotransporter-2 inhibitor*" OR gliflozin* OR canagliflozin OR dapagliflozin OR empagliflozin OR thiazolidinedione* OR TZD OR pioglitazone):ti,ab,kw) AND ("type 2 diabetes" OR T2DM):ti,ab,kw AND (NAFLD OR "fatty liver" OR MAFLD OR MASLD OR "liver enzyme"):ti,ab,kw AND (Asia OR Asian OR China OR Japan OR Korea OR India):ti,ab,kw |
| **Cochrane Library** | (("GLP-1 receptor agonist*" OR liraglutide OR dulaglutide OR semaglutide OR "SGLT2 inhibitor*" OR gliflozin* OR pioglitazone OR thiazolidinedione*):ti,ab,kw) AND (T2DM OR "type 2 diabet*"):ti,ab,kw AND (MASLD OR NAFLD OR "fatty liver"):ti,ab,kw AND (Asia* OR China OR Japan* OR Korea* OR India* OR Iran* OR Thai*):ti,ab,kw. |

# Supplementary Table S2. Detailed RoB 2 assessments for alanine aminotransferase (ALT)

**Target estimand:** effect of assignment to intervention (intention-to-treat). **Response codes:** Y, yes; PY, probably yes; PN, probably no; N, no; NI, no information; NA, not applicable.

## S2.1 Ito 2017

| **Unique ID** | Ito 2017 | | **Study ID** | UMIN000022651 | **Assessor** | | Hanwen Zheng; Jieru Han (consensus) |
| --- | --- | --- | --- | --- | --- | --- | --- |
| **Ref/label** | Diabetes Care. 2017;40(10):1364–1372. | | **Aim** | Intention-to-treat intervention effect |  | |  |
| **Experimental** | Ipragliflozin | | **Comparator** | Pioglitazone | **Source** | | Journal article + trial registry |
| **Outcome** | Alanine aminotransferase (ALT) | | **Result** | Change from baseline in serum ALT at week 24 (ipragliflozin vs pioglitazone). | **Weight** | | 1 |
| **Domain** | | **Signalling question / judgement** | | **Response** | | **Comments / justification** | |
| **Bias arising from the randomization process** | | 1.1 Was the allocation sequence random? | | Y | | Eligible participants were assigned 1:1 using a computer-generated randomization sequence (p. 2). | |
|  | | 1.2 Was the allocation sequence concealed until participants were enrolled and assigned to interventions? | | NI | | The report describes a computer-generated sequence but does not state how allocation was concealed or who implemented the sequence. Allocation concealment therefore cannot be verified from the supplied article. | |
|  | | 1.3 Did baseline differences between intervention groups suggest a problem with the randomization process? | | PN | | Most baseline characteristics were similar. Two statistically significant imbalances were reported: ARB/ACE-inhibitor use and serum type IV collagen 7S (both P = 0.013). Given the small sample and absence of a systematic imbalance across prognostic factors, these differences probably do not indicate failure of randomization, but some uncertainty remains (Table 1; pp. 3–4). | |
|  | | **Risk of bias judgement** | | **Some concerns** | | The sequence was random, but allocation concealment was not reported. The observed baseline imbalances do not clearly establish a randomization problem. | |
| **Bias due to deviations from intended interventions** | | 2.1 Were participants aware of their assigned intervention during the trial? | | Y | | The trial was explicitly described as open-label; participants therefore knew which active drug they received (pp. 1–2). | |
|  | | 2.2 Were carers and people delivering the interventions aware of participants' assigned intervention during the trial? | | Y | | Because this was an open-label active-controlled trial with different drugs and dosing procedures, treating clinicians and study personnel were aware of treatment assignment (p. 2). | |
|  | | 2.3 If Y/PY/NI to 2.1 or 2.2: Were there deviations from the intended intervention that arose because of the experimental context? | | PN | | No additions or adjustments to background antidiabetes, antihypertensive, or lipid-lowering therapy were permitted, apart from protocol-allowed reduction/discontinuation of insulin or sulfonylurea to avoid hypoglycemia. No sulfonylurea reductions occurred, and insulin-dose changes did not differ significantly between groups. Diet and exercise counselling was applied to both groups (pp. 2 and 4). | |
|  | | 2.4 If Y/PY to 2.3: Were these deviations likely to have affected the outcome? | | NA | | Not applicable because no important trial-context-related deviations from the intended interventions were identified. | |
|  | | 2.5 If Y/PY/NI to 2.4: Were these deviations from intended intervention balanced between groups? | | NA | | Not applicable because no important deviations requiring assessment of balance were identified. | |
|  | | 2.6 Was an appropriate analysis used to estimate the effect of assignment to intervention? | | Y | | The authors explicitly stated that statistical analyses were performed in an intention-to-treat population. ALT was a prespecified key secondary outcome, and Table 2 reports ALT results using the randomized group denominators (ipragliflozin n=32; pioglitazone n=34) (pp. 3-4). | |
|  | | 2.7 If N/PN/NI to 2.6: Was there potential for a substantial impact of failure to analyse participants in the group to which they were randomized? | | NA | | Not applicable because an intention-to-treat analysis by randomized group was reported. | |
|  | | **Risk of bias judgement** | | **Low** | | Although the trial was open-label, no important deviations arising from the trial context were identified, co-interventions were largely controlled, and an intention-to-treat analysis was reported. | |
| **Bias due to missing outcome data** | | 3.1 Were data for this outcome available for all, or nearly all, participants randomized? | | PN | | Sixty-one of 66 randomized participants completed the 24-week trial (30/32 ipragliflozin; 31/34 pioglitazone). The report does not state whether observed week-24 ALT measurements were available for every randomized participant, although Table 2 retains the original randomized denominators. Complete ALT data therefore cannot be confirmed (p. 4). | |
|  | | 3.2 If N/PN/NI to 3.1: Is there evidence that the result was not biased by missing outcome data? | | NI | | Although the analyses were described as intention-to-treat and Table 2 retained the randomized denominators, the report does not explain how missing week-24 ALT measurements for participants who discontinued were handled and does not provide an ALT-specific missing-data sensitivity analysis. | |
|  | | 3.3 If N/PN to 3.2: Could missingness in the outcome have depended on its true value? | | PN | | Discontinuations were relatively balanced (2/32 vs 3/34). Reasons were voluntary withdrawal and loss to follow-up in the ipragliflozin group, and two edema-related adverse events plus one loss to follow-up in the pioglitazone group. These reasons were not clearly related to the unobserved true ALT value, although a relationship cannot be completely excluded (p. 4). | |
|  | | 3.4 If Y/PY/NI to 3.3: Is it likely that missingness in the outcome depended on its true value? | | NA | | Not applicable because missingness was judged probably not to depend on the true ALT value. | |
|  | | **Risk of bias judgement** | | **Low** | | The amount of missing follow-up was modest and relatively balanced, and the reported reasons were unlikely to depend on the unobserved ALT value. The method used to handle missing ALT measurements was not fully described. | |
| **Bias in measurement of the outcome** | | 4.1 Was the method of measuring the outcome inappropriate? | | N | | Serum ALT was measured as part of routine clinical laboratory testing and is an objective biochemical outcome. The method of measuring ALT was therefore considered appropriate for the outcome assessed (p. 2). | |
|  | | 4.2 Could measurement or ascertainment of the outcome have differed between intervention groups? | | N | | Clinical laboratory tests, including ALT, were performed during follow-up for both randomized groups, and no group-specific laboratory method or assessment schedule was reported (p. 2). | |
|  | | 4.3 Were outcome assessors aware of the intervention received by study participants? | | NI | | The trial was open-label, but the article does not state whether laboratory personnel measuring serum ALT were blinded to treatment assignment. | |
|  | | 4.4 If Y/PY/NI to 4.3: Could assessment of the outcome have been influenced by knowledge of intervention received? | | PN | | Even if laboratory personnel were aware of treatment assignment, serum ALT is an objective biochemical measurement and is unlikely to be materially influenced by such knowledge. | |
|  | | 4.5 If Y/PY/NI to 4.4: Is it likely that assessment of the outcome was influenced by knowledge of intervention received? | | NA | | Not applicable because assessment of ALT was judged probably not to be influenced by knowledge of the intervention. | |
|  | | **Risk of bias judgement** | | **Low** | | ALT was measured as an objective laboratory biomarker using the same clinical assessment approach across groups. Unreported blinding of laboratory personnel was unlikely to materially influence the ALT result. | |
| **Bias in selection of the reported result** | | 5.1 Were the data that produced this result analysed in accordance with a pre-specified analysis plan finalized before unblinded outcome data were available for analysis? | | NI | | The trial was registered (UMIN000022651), and ALT was explicitly listed as a key secondary outcome with change from baseline at week 24. However, the supplied report does not provide a dated protocol or statistical analysis plan allowing confirmation that the final ALT analysis plan was fixed before unblinded outcome data were available. | |
|  | | 5.2 Was the numerical result being assessed likely to have been selected, on the basis of the results, from multiple eligible outcome measurements within the outcome domain? | | PN | | ALT was explicitly defined as a key secondary outcome assessed as change from baseline at week 24. Although laboratory tests were performed during follow-up, the target week-24 ALT result was specified in the report, making selection from multiple eligible outcome measurements unlikely (p. 3). | |
|  | | 5.3 Was the numerical result being assessed likely to have been selected, on the basis of the results, from multiple eligible analyses of the data? | | PN | | The statistical methods for continuous secondary outcomes and between-group changes were described in advance in the article, and the week-24 ALT change was reported directly in Table 2. There is no clear indication that the ALT result was selected from multiple competing analyses. | |
|  | | **Risk of bias judgement** | | **Some concerns** | | ALT was clearly identified and reported as a key secondary outcome, but the absence of an accessible dated protocol/SAP prevents full verification that the ALT analysis was prespecified before unblinded data were available. | |
| **Overall bias** | | **Risk of bias judgement** | | **Some concerns** | | Overall risk of bias for the ALT result was judged as Some concerns because at least one domain raised some concerns and no domain was judged high risk. The main concerns were unreported allocation concealment and inability to fully verify prespecification of the ALT analysis; the open-label design was unlikely to bias the objective laboratory measurement of ALT. | |

## S2.2 Kinoshita 2020

| **Unique ID** | Kinoshita 2020 | | **Study ID** | UMIN000021291 | **Assessor** | | Hanwen Zheng; Jieru Han (consensus) |
| --- | --- | --- | --- | --- | --- | --- | --- |
| **Ref/label** | J Diabetes Investig. 2020;11(6):1612–1622. | | **Aim** | Effect of assignment to intervention |  | |  |
| **Experimental** | Dapagliflozin | | **Comparator** | Pioglitazone | **Source** | | Journal article + trial registry |
| **Outcome** | Alanine aminotransferase (ALT) | | **Result** | Change from baseline in serum ALT at week 28 (dapagliflozin vs pioglitazone). | **Weight** | | 1 |
| **Domain** | | **Signalling question / judgement** | | **Response** | | **Comments / justification** | |
| **Bias arising from the randomization process** | | 1.1 Was the allocation sequence random? | | Y | | A predefined computer-generated block randomization table with a 1:1:1 allocation ratio was used; the random sequence was generated independently using computer software. | |
|  | | 1.2 Was the allocation sequence concealed until participants were enrolled and assigned to interventions? | | PY | | The allocation sequence was generated independently, and enrollment/assignment procedures were separated from treatment and data collection. Although the specific concealment mechanism was not fully described, allocation was probably adequately concealed. | |
|  | | 1.3 Did baseline differences between intervention groups suggest a problem with the randomization process? | | N | | Baseline characteristics were generally comparable across groups. No pattern of systematic baseline imbalance suggested a problem with the randomization process. | |
|  | | **Risk of bias judgement** | | **Low** | | The random sequence was appropriately generated, allocation was probably adequately concealed, and baseline characteristics provided no evidence of a randomization problem. | |
| **Bias due to deviations from intended interventions** | | 2.1 Were participants aware of their assigned intervention during the trial? | | Y | | The trial was explicitly described as open-label. | |
|  | | 2.2 Were carers and people delivering the interventions aware of participants’ assigned intervention during the trial? | | Y | | Because the trial was open-label, treating clinicians were aware of the assigned intervention. | |
|  | | 2.3 If Y/PY/NI to 2.1 or 2.2: Were there deviations from the intended intervention that arose because of the experimental context? | | PN | | Concomitant antidiabetic medications were intended to remain unchanged, and treatment guidance and dose adjustment followed the study protocol. No important differential deviations attributable to the open-label trial context were reported. | |
|  | | 2.4 If Y/PY to 2.3: Were these deviations likely to have affected the outcome? | | NA | | Not applicable because no important deviations arising from the experimental context were identified. | |
|  | | 2.5 If Y/PY/NI to 2.4: Were these deviations from intended intervention balanced between groups? | | NA | | Not applicable. | |
|  | | 2.6 Was an appropriate analysis used to estimate the effect of assignment to intervention? | | PY | | Participants with available outcome data were analysed according to their randomized treatment groups, with no indication of treatment-group reclassification. Missing outcome data were considered separately in Domain 3. | |
|  | | 2.7 If N/PN/NI to 2.6: Was there potential for a substantial impact on the result of the failure to analyse participants in the group to which they were randomized? | | NA | | Not applicable because the analysis was considered appropriate for estimating the effect of assignment to intervention. | |
|  | | **Risk of bias judgement** | | **Low** | | Although the trial was open-label, no important trial-context-related deviations were identified. Participants were analysed according to randomized assignment, while missing outcome data were addressed separately in Domain 3. | |
| **Bias due to missing outcome data** | | 3.1 Were data for this outcome available for all, or nearly all, participants randomized? | | N | | For the dapagliflozin-versus-pioglitazone comparison, ALT data were not available for all randomized participants; 32 of 40 participants randomized to dapagliflozin and 33 of 36 randomized to pioglitazone were included in the final analysis. | |
|  | | 3.2 If N/PN/NI to 3.1: Is there evidence that the result was not biased by missing outcome data? | | N | | No formal sensitivity analysis specifically addressing missing ALT data was reported. | |
|  | | 3.3 If N/PN to 3.2: Could missingness in the outcome depend on its true value? | | PN | | Reported reasons for unavailable data were related to follow-up, withdrawal, or availability of follow-up measurements rather than to participants' ALT values. There was no indication that missingness depended on the unobserved ALT outcome. | |
|  | | 3.4 If Y/PY/NI to 3.3: Is it likely that missingness in the outcome depended on its true value? | | NA | | Not applicable because dependence of missingness on the true ALT value was judged probably absent. | |
|  | | **Risk of bias judgement** | | **Low** | | Although ALT data were not available for all randomized participants, the reported reasons for missingness did not indicate dependence on the true ALT value; substantial bias from missing outcome data was therefore considered unlikely. | |
| **Bias in measurement of the outcome** | | 4.1 Was the method of measuring the outcome inappropriate? | | N | | Serum ALT was assessed as a routine biochemical laboratory parameter, which was an appropriate and objective measure of the outcome. | |
|  | | 4.2 Could measurement or ascertainment of the outcome have differed between intervention groups? | | N | | ALT was assessed using the same outcome definition and follow-up schedule across randomized treatment groups, with no indication of differential measurement procedures. | |
|  | | 4.3 Were outcome assessors aware of the intervention received by study participants? | | NI | | The study was open-label, but blinding of laboratory personnel responsible for ALT measurement was not specifically reported. | |
|  | | 4.4 If Y/PY/NI to 4.3: Could assessment of the outcome have been influenced by knowledge of intervention received? | | PN | | ALT is an objectively measured laboratory biomarker, and knowledge of treatment assignment was unlikely to materially influence the measured value. | |
|  | | 4.5 If Y/PY/NI to 4.4: Is it likely that assessment of the outcome was influenced by knowledge of intervention received? | | NA | | Not applicable because influence of treatment knowledge on ALT measurement was judged probably absent. | |
|  | | **Risk of bias judgement** | | **Low** | | ALT was an objective laboratory outcome measured similarly across groups; the absence of reported assessor blinding was unlikely to introduce meaningful measurement bias. | |
| **Bias in selection of the reported result** | | 5.1 Were the data that produced this result analysed in accordance with a pre-specified analysis plan that was finalized before unblinded outcome data were available for analysis? | | PY | | The trial was prospectively registered (UMIN000021291), and the registered outcomes included change in liver function at approximately six months after intervention, consistent with the reported 28-week ALT assessment. | |
|  | | 5.2 Is the numerical result being assessed likely to have been selected, on the basis of the results, from multiple eligible outcome measurements within the outcome domain? | | PN | | ALT was reported at the approximately six-month follow-up, consistent with the registered liver-function outcome. There was no indication that the reported ALT result was selected from multiple eligible measurements on the basis of the findings. | |
|  | | 5.3 Is the numerical result being assessed likely to have been selected, on the basis of the results, from multiple eligible analyses of the data? | | PN | | The article described the statistical methods used for treatment-group comparisons, with no indication that the ALT result was selected from multiple competing analyses on the basis of statistical significance or effect magnitude. | |
|  | | **Risk of bias judgement** | | **Low** | | The timing and domain of the reported ALT outcome were consistent with the prospectively documented trial outcomes, with no evidence of selective choice among multiple eligible measurements or analyses. | |
| **Overall bias** | | **Risk of bias judgement** | | **Low** | | Overall risk of bias for the ALT result was judged Low. All five domains were judged Low: randomization was adequate, the open-label design did not produce identified trial-context-related deviations, missing ALT data were unlikely to depend on the true outcome value, ALT measurement was objective, and the reported ALT assessment was consistent with the prospectively documented liver-function outcome. | |

## S2.3 Shi 2023

| **Unique ID** | Shi 2023 | | **Study ID** | ChiCTR2100054612 | **Assessor** | | Hanwen Zheng; Jieru Han (consensus) |
| --- | --- | --- | --- | --- | --- | --- | --- |
| **Ref/label** | J Diabetes Complications. 2023;37(10):108610. | | **Aim** | Effect of assignment to intervention (intention-to-treat effect) |  | |  |
| **Experimental** | Dapagliflozin 10 mg/day + Background | | **Comparator** | Background | **Source** | | Journal article + trial registry |
| **Outcome** | Alanine aminotransferase (ALT) | | **Result** | Change from baseline in serum ALT at week 24: dapagliflozin −12.09 U/L vs control −4.07 U/L; between-group P < 0.001. | **Weight** | | 1 |
| **Domain** | | **Signalling question / judgement** | | **Response** | | **Comments / justification** | |
| **Bias arising from the randomization process** | | 1.1 Was the allocation sequence random? | | Y | | Eligible participants were assigned 1:1 using computer-generated random numbers (article p. 2). | |
|  | | 1.2 Was the allocation sequence concealed until participants were enrolled and assigned to interventions? | | NI | | Allocation concealment was not described. The report states that a researcher assigned participants according to computer-generated random numbers and the order of registration, but no central allocation or sealed-envelope procedure was reported (article p. 2). | |
|  | | 1.3 Did baseline differences between intervention groups suggest a problem with the randomization process? | | N | | Baseline characteristics were comparable between groups; baseline ALT was 39.6 (23.5, 61.7) U/L in the dapagliflozin group and 44.4 (23.5, 69.0) U/L in controls (P = 0.715) (Table 1). | |
|  | | **Risk of bias judgement** | | **Some concerns** | | The random sequence was generated appropriately, but allocation concealment was not reported. | |
| **Bias due to deviations from intended interventions** | | 2.1 Were participants aware of their assigned intervention during the trial? | | Y | | The trial was explicitly described as open-label, so participants knew their assigned add-on treatment. | |
|  | | 2.2 Were carers and people delivering the interventions aware of participants' assigned intervention during the trial? | | Y | | Because treatment allocation was open-label, clinicians/researchers delivering the assigned glucose-lowering treatment were aware of group assignment. | |
|  | | 2.3 If Y/PY/NI to 2.1 or 2.2: Were there deviations from the intended intervention that arose because of the experimental context? | | PN | | Both groups received the assigned add-on therapy on top of metformin, identical baseline dietary and exercise advice, and the same scheduled follow-up visits. No unplanned differential co-intervention caused by awareness of allocation was reported. | |
|  | | 2.4 If Y/PY to 2.3: Were these deviations likely to have affected the outcome? | | NA | | Not applicable because deviations arising from the experimental context were judged probably absent. | |
|  | | 2.5 If Y/PY/NI to 2.4: Were these deviations from intended intervention balanced between groups? | | NA | | Not applicable because question 2.4 was not required. | |
|  | | 2.6 Was an appropriate analysis used to estimate the effect of assignment to intervention? | | Y | | Participants with available ALT data were analysed according to their randomized groups. No treatment switching or reclassification was reported; missing outcome data are considered separately in Domain 3. | |
|  | | 2.7 If N/PN/NI to 2.6: Was there potential for a substantial impact (on the result) of the failure to analyse participants in the group to which they were randomized? | | NA | | Not applicable because question 2.6 was answered Yes. | |
|  | | **Risk of bias judgement** | | **Low** | | Although the study was open-label, no important deviations from intended intervention arising from the trial context were identified, and available ALT data were compared according to randomized assignment. | |
| **Bias due to missing outcome data** | | 3.1 Were data for this outcome available for all, or nearly all, participants randomized? | | PN | | Week-24 analyses included 78 of 84 randomized participants (92.9%): 40/42 in the dapagliflozin group and 38/42 in the control group. ALT results in Table 2 were based on these analysed participants. | |
|  | | 3.2 If N/PN/NI to 3.1: Is there evidence that the result was not biased by missing outcome data? | | NI | | Reasons for missing follow-up were reported, but no imputation, missing-data sensitivity analysis, or other analysis specifically demonstrating robustness of the ALT result to missing data was reported. | |
|  | | 3.3 If N/PN to 3.2: Could missingness in the outcome depend on its true value? | | PY | | Missingness could plausibly be related to subsequent ALT values because reasons included treatment discontinuation, bariatric surgery, loss to follow-up, and excessive missing values. | |
|  | | 3.4 If Y/PY/NI to 3.3: Is it likely that missingness in the outcome depended on its true value? | | PN | | The number of missing participants was small and the reasons were heterogeneous; the report does not show that missingness was likely to depend on the unobserved ALT values. | |
|  | | **Risk of bias judgement** | | **Some concerns** | | ALT data were unavailable for 6 of 84 randomized participants, with more missing participants in the control group, and no sensitivity analysis for missing ALT data was reported. | |
| **Bias in measurement of the outcome** | | 4.1 Was the method of measuring the outcome inappropriate? | | N | | ALT was measured as a routine biochemical laboratory outcome using the hospital laboratory system; the report states that blood biochemical indices were assessed using the Sysmex XN-9000 flow-cell line. | |
|  | | 4.2 Could measurement or ascertainment of the outcome have differed between intervention groups? | | N | | The same hospital laboratory procedures and scheduled assessments were used for both randomized groups. | |
|  | | 4.3 Were outcome assessors aware of the intervention received by study participants? | | NI | | Blinding of laboratory personnel to treatment assignment was not reported. | |
|  | | 4.4 If Y/PY/NI to 4.3: Could assessment of the outcome have been influenced by knowledge of intervention received? | | N | | ALT is an objective automated biochemical measurement; knowledge of treatment assignment would not be expected to materially influence the measured enzyme concentration. | |
|  | | 4.5 If Y/PY/NI to 4.4: Is it likely that assessment of the outcome was influenced by knowledge of intervention received? | | NA | | Not applicable because question 4.4 was answered No. | |
|  | | **Risk of bias judgement** | | **Low** | | ALT was measured objectively using the same laboratory method in both groups, and any awareness of treatment assignment was unlikely to influence the assay result. | |
| **Bias in selection of the reported result** | | 5.1 Were the data that produced this result analysed in accordance with a pre-specified analysis plan that was finalized before unblinded outcome data were available for analysis? | | NI | | The trial registration number (ChiCTR2100054612) was reported and ALT was identified as a secondary endpoint, but the article does not provide a dated protocol or statistical analysis plan confirming that the specific ALT analysis was finalized before unblinded outcome data were available. | |
|  | | 5.2 Is the numerical result being assessed likely to have been selected, on the basis of the results, from multiple eligible outcome measurements (e.g. scales, definitions, time points) within the outcome domain? | | N | | ALT change from baseline to week 24 was explicitly defined as a secondary endpoint. Although week-12 laboratory values were also reported, week 24 was the prespecified endpoint for the treatment comparison. | |
|  | | 5.3 Is the numerical result being assessed likely to have been selected, on the basis of the results, from multiple eligible analyses of the data? | | N | | The article prespecified standard paired and between-group tests for continuous outcomes and gives no indication that the reported week-24 ALT result was selected from multiple competing analyses. | |
|  | | **Risk of bias judgement** | | **Some concerns** | | ALT and the week-24 time point were prespecified, but the absence of a verifiable dated analysis plan prevents confirmation that the exact analysis was fixed before outcome data were available. | |
| **Overall bias** | | **Risk of bias judgement** | | **Some concerns** | | Overall risk of bias for the week-24 ALT result was judged Some concerns, mainly because allocation concealment was not reported, some randomized participants lacked follow-up ALT data, and prespecification of the exact statistical analysis could not be verified. | |

## S2.4 Kuchay 2020

| **Unique ID** | Kuchay 2020 | | **Study ID** | NCT03590626 | **Assessor** | | Hanwen Zheng; Jieru Han (consensus) |
| --- | --- | --- | --- | --- | --- | --- | --- |
| **Ref/label** | Diabetologia. 2020;63(11):2434–2445. | | **Aim** | Effect of assignment to intervention (intention-to-treat estimand) |  | |  |
| **Experimental** | Dulaglutide 0.75 mg weekly for 4 weeks, then 1.5 mg weekly for 20 weeks + standard diabetes care | | **Comparator** | Standard diabetes care without GLP-1 receptor agonist | **Source** | | Journal article; ClinicalTrials.gov NCT03590626 |
| **Outcome** | Alanine aminotransferase (ALT) | | **Result** | Change in serum ALT from baseline to week 24 (per-protocol analysis): dulaglutide -26.4 U/L (SE 5.5; n=27) vs control -13.9 U/L (SE 5.0; n=25); mean between-group difference -13.1 U/L (95% CI -24.4 to 2.5), P=0.10. | **Weight** | | 1 |
| **Domain** | | **Signalling question / judgement** | | **Response** | | **Comments / justification** | |
| **Bias arising from the randomization process** | | 1.1 Was the allocation sequence random? | | Y | | A predefined computer-generated random-number sequence was used for 1:1 allocation. | |
|  | | 1.2 Was the allocation sequence concealed until participants were enrolled and assigned to interventions? | | Y | | Allocation was concealed using serially numbered, opaque, sealed envelopes prepared before assignment. | |
|  | | 1.3 Did baseline differences between intervention groups suggest a problem with the randomization process? | | N | | Baseline demographic, metabolic and liver-related characteristics were comparable between randomized groups, with no pattern suggesting failed randomization. | |
|  | | **Risk of bias judgement** | | **Low** | | The random sequence and allocation concealment were adequately described, and no important baseline imbalance relevant to ALT was identified. | |
| **Bias due to deviations from intended interventions** | | 2.1 Were participants aware of their assigned intervention during the trial? | | Y | | The trial was open-label, so participants knew whether dulaglutide had been added to usual care. | |
|  | | 2.2 Were carers and people delivering the interventions aware of participants' assigned intervention during the trial? | | Y | | Treating clinicians were aware of allocation because treatment was open-label and background glucose-lowering therapy was adjusted clinically. | |
|  | | 2.3 If Y/PY/NI to 2.1 or 2.2: Were there deviations from the intended intervention that arose because of the experimental context? | | PN | | No crossover or unplanned differential co-intervention was reported. Lifestyle advice and diabetes management were standardized; stopping DPP-4 inhibitors in the dulaglutide group was protocol-specified. | |
|  | | 2.4 If Y/PY to 2.3: Were these deviations likely to have affected the outcome? | | NA | | Not applicable because important deviations arising from the experimental context were judged probably absent. | |
|  | | 2.5 If Y/PY/NI to 2.4: Were these deviations from intended intervention balanced between groups? | | NA | | Not applicable because question 2.4 was not required. | |
|  | | 2.6 Was an appropriate analysis used to estimate the effect of assignment to intervention? | | Y | | The ALT comparison retained participants in their randomized treatment groups. Exclusion of participants without week-24 data is considered in Domain 3 rather than as reclassification by treatment received. | |
|  | | 2.7 If N/PN/NI to 2.6: Was there potential for a substantial impact (on the result) of the failure to analyse participants in the group to which they were randomized? | | NA | | Not applicable because the randomized-group comparison was preserved. | |
|  | | **Risk of bias judgement** | | **Low** | | Open-label status did not itself create a bias: no important trial-context deviations were identified, co-interventions were protocol-guided, and analyzed participants remained in their randomized groups. | |
| **Bias due to missing outcome data** | | 3.1 Were data for this outcome available for all, or nearly all, participants randomized? | | N | | Week-24 ALT data in the reported per-protocol table were available for 52 of 64 randomized participants: 27/32 in the dulaglutide group and 25/32 in the control group. | |
|  | | 3.2 If N/PN/NI to 3.1: Is there evidence that the result was not biased by missing outcome data? | | PY | | Attrition was similar between groups (5 vs 7). Reported reasons included gastrointestinal adverse effects, loss to follow-up, and protocol violations; there was no clear indication that missingness was driven by the unobserved ALT value, so material bias in the ALT contrast was judged unlikely. | |
|  | | 3.3 If N/PN to 3.2: Could missingness in the outcome depend on its true value? | | NA | | Not applicable because question 3.2 was answered Probably yes. | |
|  | | 3.4 If Y/PY/NI to 3.3: Is it likely that missingness in the outcome depended on its true value? | | NA | | Not applicable because question 3.3 was not required. | |
|  | | **Risk of bias judgement** | | **Low** | | Although ALT was reported for 52/64 randomized participants, attrition was reasonably balanced and documented, with no clear evidence that missingness depended on the unobserved ALT value. | |
| **Bias in measurement of the outcome** | | 4.1 Was the method of measuring the outcome inappropriate? | | N | | Serum ALT is a standard objective biochemical laboratory measurement and was an appropriate method for this outcome. | |
|  | | 4.2 Could measurement or ascertainment of the outcome have differed between intervention groups? | | N | | The same clinical laboratory outcome and follow-up schedule were used for both randomized groups; no group-specific ALT measurement procedure was reported. | |
|  | | 4.3 Were outcome assessors aware of the intervention received by study participants? | | NI | | The report did not specifically state whether laboratory personnel measuring ALT were blinded to treatment allocation. | |
|  | | 4.4 If Y/PY/NI to 4.3: Could assessment of the outcome have been influenced by knowledge of intervention received? | | PN | | Even if laboratory personnel were aware of treatment assignment, the numerical ALT concentration generated by routine laboratory testing is unlikely to be materially influenced by such knowledge. | |
|  | | 4.5 If Y/PY/NI to 4.4: Is it likely that assessment of the outcome was influenced by knowledge of intervention received? | | NA | | Not applicable because question 4.4 was answered Probably no. | |
|  | | **Risk of bias judgement** | | **Low** | | ALT was an objective laboratory outcome measured comparably across groups; lack of explicit laboratory blinding was unlikely to influence the measured value. | |
| **Bias in selection of the reported result** | | 5.1 Were the data that produced this result analysed in accordance with a pre-specified analysis plan that was finalized before unblinded outcome data were available for analysis? | | PY | | The trial was prospectively registered (NCT03590626). The registry specified change in serum ALT from baseline to 24 weeks as a secondary outcome and described the planned statistical approach before study completion. | |
|  | | 5.2 ... multiple eligible outcome measurements (e.g. scales, definitions, time points) within the outcome domain? | | N | | ALT was defined as change from baseline to the fixed 24-week endpoint; no alternative eligible ALT scale or final time point was reported. | |
|  | | 5.3 ... multiple eligible analyses of the data? | | N | | The article reported the planned between-group change analysis for ALT with effect estimate, confidence interval and P value, with no indication that the published ALT result was selected from competing analyses. | |
|  | | **Risk of bias judgement** | | **Low** | | Prospective registration of ALT as a 24-week secondary outcome and transparent reporting of the prespecified comparison supported Low risk for selective reporting. | |
| **Overall bias** | | **Risk of bias judgement** | | **Low** | | All five RoB 2 domains were judged Low for the ALT result. The trial was open-label, but no important deviations from intended intervention were identified; ALT was objectively measured, attrition was balanced with documented reasons, and ALT was prospectively specified as a 24-week secondary outcome. | |

## S2.5 Attaran 2023

| **Unique ID** | Attaran 2023 | | **Study ID** | IRCT20190122042450N5 | **Assessor** | | Hanwen Zheng; Jieru Han (consensus) |
| --- | --- | --- | --- | --- | --- | --- | --- |
| **Ref/label** | BMC Gastroenterol. 2023;23(1):327. | | **Aim** | Effect of assignment to intervention (intention-to-treat estimand) |  | |  |
| **Experimental** | Empagliflozin 10 mg/day for 24 weeks + background diabetes care | | **Comparator** | Pioglitazone 30 mg/day for 24 weeks + background diabetes care | **Source** | | Journal article; Iranian Registry of Clinical Trials IRCT20190122042450N5 |
| **Outcome** | Alanine aminotransferase (ALT) | | **Result** | Serum ALT from baseline to week 24: empagliflozin 31.8 ± 17.6 to 22.2 ± 11.0 IU/L vs pioglitazone 31.5 ± 12.2 to 21.0 ± 8.6 IU/L; both within-group P<0.01; between-group difference in change P=0.76. | **Weight** | | 1 |
| **Domain** | | **Signalling question / judgement** | | **Response** | | **Comments / justification** | |
| **Bias arising from the randomization process** | | 1.1 Was the allocation sequence random? | | Y | | A computerized block-randomization method was used to allocate participants 1:1. | |
|  | | 1.2 Was the allocation sequence concealed until participants were enrolled and assigned to interventions? | | Y | | Study medications were sealed, sequentially numbered according to the allocation sequence, and an independent staff member generated the sequence and assigned participants. | |
|  | | 1.3 Did baseline differences between intervention groups suggest a problem with the randomization process? | | N | | Baseline age, sex, BMI, diabetes duration, HbA1c and ALT were comparable; the isolated triglyceride difference did not suggest a systematic problem with randomization. | |
|  | | **Risk of bias judgement** | | **Low** | | Computerized block randomization, sequentially numbered sealed allocation, independent assignment and broadly balanced baseline characteristics supported Low risk. | |
| **Bias due to deviations from intended interventions** | | 2.1 Were participants aware of their assigned intervention during the trial? | | NI | | The study was described as single-blind, but the report does not clearly state that participants themselves were blinded. | |
|  | | 2.2 Were carers and people delivering the interventions aware of participants' assigned intervention during the trial? | | N | | The article explicitly states that the care provider was blinded to the intervention. | |
|  | | 2.3 If Y/PY/NI to 2.1 or 2.2: Were there deviations from the intended intervention that arose because of the experimental context? | | N | | No important trial-context deviation was identified. Both groups received standard lifestyle advice; background anti-hyperglycemic therapy could be adjusted according to the same ADA guidance, while study-drug doses remained constant. | |
|  | | 2.4 If Y/PY to 2.3: Were these deviations likely to have affected the outcome? | | NA | | Not applicable because no important trial-context deviation was identified. | |
|  | | 2.5 If Y/PY/NI to 2.4: Were these deviations from intended intervention balanced between groups? | | NA | | Not applicable because question 2.4 was not required. | |
|  | | 2.6 Was an appropriate analysis used to estimate the effect of assignment to intervention? | | Y | | The authors explicitly state that data were analyzed by the intention-to-treat method and all 73 randomized participants were included according to assigned group. | |
|  | | 2.7 If N/PN/NI to 2.6: Was there potential for a substantial impact (on the result) of the failure to analyse participants in the group to which they were randomized? | | NA | | Not applicable because an intention-to-treat analysis was used. | |
|  | | **Risk of bias judgement** | | **Low** | | Care-provider blinding, absence of important differential deviations, and explicit intention-to-treat analysis supported Low risk for deviations from intended intervention. | |
| **Bias due to missing outcome data** | | 3.1 Were data for this outcome available for all, or nearly all, participants randomized? | | PY | | Seventy-three participants were randomized; three did not complete treatment because of non-adherence, but the article states that data from all 73 randomized participants were used in the intention-to-treat analysis. | |
|  | | 3.2 If N/PN/NI to 3.1: Is there evidence that the result was not biased by missing outcome data? | | NA | | Not applicable because outcome data were judged available for all or nearly all participants under the reported intention-to-treat analysis. | |
|  | | 3.3 If N/PN to 3.2: Could missingness in the outcome depend on its true value? | | NA | | Not applicable. | |
|  | | 3.4 If Y/PY/NI to 3.3: Is it likely that missingness in the outcome depended on its true value? | | NA | | Not applicable. | |
|  | | **Risk of bias judgement** | | **Low** | | Only three participants discontinued treatment, and all randomized participants were retained in the reported intention-to-treat analysis. | |
| **Bias in measurement of the outcome** | | 4.1 Was the method of measuring the outcome inappropriate? | | N | | ALT was measured from fasting blood samples using Pars biochemical kits with a photometric method, an appropriate standardized laboratory approach. | |
|  | | 4.2 Could measurement or ascertainment of the outcome have differed between intervention groups? | | N | | The same biochemical method and follow-up schedule were applied to both treatment groups, with no indication of differential ascertainment. | |
|  | | 4.3 Were outcome assessors aware of the intervention received by study participants? | | NI | | Blinding of laboratory personnel measuring ALT was not specifically reported. | |
|  | | 4.4 If Y/PY/NI to 4.3: Could assessment of the outcome have been influenced by knowledge of intervention received? | | PN | | ALT is an objective numerical biochemical measurement, so knowledge of treatment allocation would be unlikely to materially influence the measured concentration. | |
|  | | 4.5 If Y/PY/NI to 4.4: Is it likely that assessment of the outcome was influenced by knowledge of intervention received? | | NA | | Not applicable because question 4.4 was answered Probably no. | |
|  | | **Risk of bias judgement** | | **Low** | | ALT was measured objectively using the same standardized biochemical method in both groups; explicit laboratory blinding was not necessary for a Low judgement because measurement was unlikely to be influenced by allocation knowledge. | |
| **Bias in selection of the reported result** | | 5.1 Were the data that produced this result analysed in accordance with a pre-specified analysis plan that was finalized before unblinded outcome data were available for analysis? | | PY | | The trial was registered and the protocol/statistical methods were established before reporting. Liver enzymes were collected using a standardized prespecified procedure at the fixed 24-week follow-up, and the published ALT result used the stated within- and between-group analyses. | |
|  | | 5.2 ... multiple eligible outcome measurements (e.g. scales, definitions, time points) within the outcome domain? | | N | | ALT was reported using a single laboratory definition at baseline and week 24; no alternative eligible ALT scale or competing final time point was reported. | |
|  | | 5.3 ... multiple eligible analyses of the data? | | N | | The article reported the standard within-group and ANOVA between-group comparison described in the Methods, with no evidence that the ALT result was selected from multiple alternative analyses. | |
|  | | **Risk of bias judgement** | | **Low** | | A registered trial, fixed follow-up, standardized ALT measurement, and use of the stated statistical approach provided no indication of selective choice among multiple ALT measurements or analyses. | |
| **Overall bias** | | **Risk of bias judgement** | | **Low** | | All five RoB 2 domains were judged Low for the ALT result: randomization and concealment were adequate, analysis was intention-to-treat, missingness was minimal, ALT was objectively measured, and no evidence of selective reporting of the 24-week ALT result was identified. | |

## S2.6 Kuchay 2018

| **Unique ID** | Kuchay 2018 | | **Study ID** | NCT02686476 | **Assessor** | | Hanwen Zheng; Jieru Han (consensus) |
| --- | --- | --- | --- | --- | --- | --- | --- |
| **Ref/label** | Diabetes Care. 2018;41(8):1801–1808. | | **Aim** | assignment to intervention (the 'intention-to-treat' effect) |  | |  |
| **Experimental** | Empagliflozin | | **Comparator** | Standard diabetes care | **Source** | | Journal article + ClinicalTrials.gov registration (NCT02686476) |
| **Outcome** | Alanine aminotransferase (ALT) | | **Result** | Change in serum ALT from baseline to week 20: empagliflozin 64.3 to 49.7 U/L vs control 65.3 to 61.6 U/L; between-group difference in change = -10.9 U/L, P = 0.005. | **Weight** | | 1 |
| **Domain** | | **Signalling question / judgement** | | **Response** | | **Comments / justification** | |
| **Bias arising from the randomization process** | | 1.1 Was the allocation sequence random? | | Y | | A research assistant randomized participants 1:1 using computer-generated numbers. | |
|  | | 1.2 Was the allocation sequence concealed until participants were enrolled and assigned to interventions? | | NI | | The paper has a section titled randomization and allocation concealment, but it does not describe a specific concealment mechanism such as central randomization or sealed opaque envelopes. | |
|  | | 1.3 Did baseline differences between intervention groups suggest a problem with the randomization process? | | N | | The groups were reported to have similar baseline characteristics, with no pattern suggesting failure of randomization. | |
|  | | **Risk of bias judgement** | | **Some concerns** | | The sequence was random, but allocation concealment before assignment was not described clearly. | |
| **Bias due to deviations from intended interventions** | | 2.1 Were participants aware of their assigned intervention during the trial? | | Y | | The E-LIFT trial was explicitly open-label, so participants knew whether empagliflozin was added. | |
|  | | 2.2 Were carers and people delivering the interventions aware of participants' assigned intervention during the trial? | | Y | | Treating endocrinologists adjusted diabetes treatment according to randomized assignment and therefore were aware of allocation. | |
|  | | 2.3 If Y/PY/NI to 2.1 or 2.2: Were there deviations from the intended intervention that arose because of the experimental context? | | PN | | Background glucose-lowering treatment could be adjusted to maintain glycaemic equipoise, but this was part of the planned treatment strategy. No treatment crossover or unplanned differential co-intervention was reported. | |
|  | | 2.4 If Y/PY to 2.3: Were these deviations likely to have affected the outcome? | | NA | | Not applicable because deviations arising from the experimental context were judged probably absent. | |
|  | | 2.5 If Y/PY/NI to 2.4: Were these deviations from intended intervention balanced between groups? | | NA | | Not applicable. | |
|  | | 2.6 Was an appropriate analysis used to estimate the effect of assignment to intervention? | | PY | | Participants with available ALT data were analysed according to their randomized groups. Exclusion of participants without week-20 measurements is addressed under missing outcome data rather than as a per-protocol reclassification. | |
|  | | 2.7 If N/PN/NI to 2.6: Was there potential for a substantial impact (on the result) of the failure to analyse participants in the group to which they were randomized? | | NA | | Not applicable because question 2.6 was answered probably yes. | |
|  | | **Risk of bias judgement** | | **Low** | | Although the trial was open-label, no important trial-context deviations from the intended interventions were identified, and analysed participants remained in their randomized groups. | |
| **Bias due to missing outcome data** | | 3.1 Were data for this outcome available for all, or nearly all, participants randomized? | | N | | Week-20 outcome data were available for 42 of 50 randomized participants (22/25 empagliflozin and 20/25 control), so ALT data were not available for all or nearly all participants. | |
|  | | 3.2 If N/PN/NI to 3.1: Is there evidence that the result was not biased by missing outcome data? | | PY | | Reasons for missing follow-up were reported and were not suggestive of the unobserved ALT value: drug-related complications in the empagliflozin arm and loss to follow-up or work-schedule conflict in the control arm. Missingness was also reasonably balanced. | |
|  | | 3.3 If N/PN to 3.2: Could missingness in the outcome depend on its true value? | | NA | | Not applicable because question 3.2 was answered probably yes. | |
|  | | 3.4 If Y/PY/NI to 3.3: Is it likely that missingness in the outcome depended on its true value? | | NA | | Not applicable. | |
|  | | **Risk of bias judgement** | | **Low** | | Despite moderate attrition, the documented reasons for missing follow-up were unlikely to depend on the true ALT value, so important bias from missing ALT data was considered unlikely. | |
| **Bias in measurement of the outcome** | | 4.1 Was the method of measuring the outcome inappropriate? | | N | | Serum ALT is an objective biochemical laboratory measurement and is an appropriate method for this outcome. | |
|  | | 4.2 Could measurement or ascertainment of the outcome have differed between intervention groups? | | N | | There was no indication that ALT was measured differently between the randomized groups. | |
|  | | 4.3 Were outcome assessors aware of the intervention received by study participants? | | NI | | Blinding of laboratory personnel to treatment allocation was not reported. | |
|  | | 4.4 If Y/PY/NI to 4.3: Could assessment of the outcome have been influenced by knowledge of intervention received? | | PN | | Even if laboratory personnel were aware of allocation, an automated serum ALT measurement is unlikely to be materially influenced by such knowledge. | |
|  | | 4.5 If Y/PY/NI to 4.4: Is it likely that assessment of the outcome was influenced by knowledge of intervention received? | | NA | | Not applicable because question 4.4 was answered probably no. | |
|  | | **Risk of bias judgement** | | **Low** | | ALT was an objective laboratory outcome measured in the same way across groups; lack of reported laboratory blinding was unlikely to affect the numerical ALT result. | |
| **Bias in selection of the reported result** | | 5.1 Were the data that produced this result analysed in accordance with a pre-specified analysis plan that was finalized before unblinded outcome data were available for analysis? | | NI | | ALT was reported as a secondary outcome in the article, but prospective prespecification of ALT in the trial registry could not be verified. The paper transparently reports protocol changes to sample size and follow-up duration and states that no interim analysis was performed, making data-driven selection of the ALT result unlikely. | |
|  | | 5.2 ... multiple eligible outcome measurements (e.g. scales, definitions, time points) within the outcome domain? | | N | | The reported ALT result used the defined serum ALT measure at the final 20-week assessment; no competing ALT definition or eligible final time point was reported. | |
|  | | 5.3 ... multiple eligible analyses of the data? | | N | | The article described the planned between-group and within-group statistical comparisons and did not indicate selection from multiple alternative ALT analyses. | |
|  | | **Risk of bias judgement** | | **Some concerns** | | ALT was transparently reported at the final 20-week assessment, but prospective prespecification of the ALT analysis could not be fully verified; selective reporting therefore cannot be ruled out completely. | |
| **Overall bias** | | **Risk of bias judgement** | | **Some concerns** | | Overall risk of bias was judged Some concerns because allocation concealment was not described sufficiently (Domain 1) and prospective prespecification of the ALT analysis could not be fully verified (Domain 5); the remaining domains were judged Low. | |

## S2.7 Guo 2020

| **Unique ID** | Guo 2020 | | **Study ID** | ChiCTR2000035091 | **Assessor** | | Hanwen Zheng; Jieru Han (consensus) |
| --- | --- | --- | --- | --- | --- | --- | --- |
| **Ref/label** | Diabetes Res Clin Pract. 2020;170:108487. | | **Aim** | assignment to intervention (the 'intention-to-treat' effect) |  | |  |
| **Experimental** | Liraglutide | | **Comparator** | Placebo | **Source** | | Journal article + Chinese Clinical Trial Registry record (ChiCTR2000035091) |
| **Outcome** | Alanine aminotransferase (ALT) | | **Result** | Change in serum ALT from baseline to week 26: liraglutide 33.2 to 27.2 U/L (change -6.0 +/- 14.5) vs placebo 30.5 to 30.6 U/L (change approximately 0); ALT reduction was greater with liraglutide than placebo, P < 0.05. | **Weight** | | 1 |
| **Domain** | | **Signalling question / judgement** | | **Response** | | **Comments / justification** | |
| **Bias arising from the randomization process** | | 1.1 Was the allocation sequence random? | | Y | | Patients were randomized by the trial pharmacist using computer-generated numbers with a 1:1:1 allocation ratio and block size of six. | |
|  | | 1.2 Was the allocation sequence concealed until participants were enrolled and assigned to interventions? | | PY | | Allocation was implemented by the trial pharmacist rather than the treating clinicians. Although the concealment procedure was not described in full detail, assignment was probably concealed until allocation. | |
|  | | 1.3 Did baseline differences between intervention groups suggest a problem with the randomization process? | | N | | Baseline demographic, clinical, laboratory, and imaging characteristics were reported as similar across groups, with no important pattern suggesting a randomization problem. | |
|  | | **Risk of bias judgement** | | **Low** | | Computer-generated blocked randomization was implemented by a trial pharmacist, and there was no evidence of a baseline imbalance indicating failure of randomization. | |
| **Bias due to deviations from intended interventions** | | 2.1 Were participants aware of their assigned intervention during the trial? | | Y | | The trial was explicitly described as not blinded, so participants were aware of the assigned treatment. | |
|  | | 2.2 Were carers and people delivering the interventions aware of participants' assigned intervention during the trial? | | Y | | The active treatments required drug-specific administration and dose adjustment, so personnel delivering treatment were aware of allocation. | |
|  | | 2.3 If Y/PY/NI to 2.1 or 2.2: Were there deviations from the intended intervention that arose because of the experimental context? | | PN | | Metformin was maintained at a constant dose and all groups received the same lifestyle advice. Dose titration of study drugs was protocol-driven, and no unplanned differential co-intervention or crossover was reported. | |
|  | | 2.4 If Y/PY to 2.3: Were these deviations likely to have affected the outcome? | | NA | | Not applicable because question 2.3 was answered probably no. | |
|  | | 2.5 If Y/PY/NI to 2.4: Were these deviations from intended intervention balanced between groups? | | NA | | Not applicable. | |
|  | | 2.6 Was an appropriate analysis used to estimate the effect of assignment to intervention? | | PY | | Participants with available week-26 ALT data were analysed in their originally assigned groups; there was no indication of treatment switching or reclassification by treatment received. | |
|  | | 2.7 If N/PN/NI to 2.6: Was there potential for a substantial impact (on the result) of the failure to analyse participants in the group to which they were randomized? | | NA | | Not applicable because question 2.6 was answered probably yes. | |
|  | | **Risk of bias judgement** | | **Low** | | Open-label treatment did not lead to identified important deviations from the intended interventions, and the ALT comparison retained randomized group assignment. | |
| **Bias due to missing outcome data** | | 3.1 Were data for this outcome available for all, or nearly all, participants randomized? | | PY | | Ninety-one of 96 randomized participants completed the 26-week trial (30 insulin glargine, 31 liraglutide, 30 placebo), so ALT data were available for nearly all randomized participants. | |
|  | | 3.2 If N/PN/NI to 3.1: Is there evidence that result was not biased by missing outcome data? | | NA | | Not applicable because question 3.1 was answered probably yes. | |
|  | | 3.3 If N/PN to 3.2: Could missingness in the outcome depend on its true value? | | NA | | Not applicable. | |
|  | | 3.4 If Y/PY/NI to 3.3: Is it likely that missingness in the outcome depended on its true value? | | NA | | Not applicable. | |
|  | | **Risk of bias judgement** | | **Low** | | Only five of 96 randomized participants did not complete follow-up, so the small amount of missing ALT data was unlikely to materially bias the result. | |
| **Bias in measurement of the outcome** | | 4.1 Was the method of measuring the outcome inappropriate? | | N | | Serum ALT is an objective biochemical laboratory measure and was appropriate for assessing liver enzyme change. | |
|  | | 4.2 Could measurement or ascertainment of the outcome have differed between intervention groups? | | N | | ALT was assessed using the same clinical laboratory procedures and schedule in all randomized groups. | |
|  | | 4.3 Were outcome assessors aware of the intervention received by study participants? | | NI | | The study was open-label and did not report whether laboratory personnel were blinded to allocation. | |
|  | | 4.4 If Y/PY/NI to 4.3: Could assessment of the outcome have been influenced by knowledge of intervention received? | | PN | | Knowledge of allocation would be unlikely to materially influence an automated serum ALT concentration. | |
|  | | 4.5 If Y/PY/NI to 4.4: Is it likely that assessment of the outcome was influenced by knowledge of intervention received? | | NA | | Not applicable because question 4.4 was answered probably no. | |
|  | | **Risk of bias judgement** | | **Low** | | ALT was an objective laboratory outcome measured consistently between groups; lack of reported laboratory blinding was unlikely to influence the result. | |
| **Bias in selection of the reported result** | | 5.1 Were the data that produced this result analysed in accordance with a pre-specified analysis plan that was finalized before unblinded outcome data were available for analysis? | | NI | | ALT and the week-26 comparison were described in the article, but the available report does not establish that a detailed ALT analysis plan was finalized before unblinded outcome data were available; the reported registry identifier does not by itself resolve this timing issue. | |
|  | | 5.2 ... multiple eligible outcome measurements (e.g. scales, definitions, time points) within the outcome domain? | | N | | ALT was assessed as a single liver-enzyme outcome at the prespecified end-of-treatment time point, with no alternative eligible ALT measurement reported. | |
|  | | 5.3 ... multiple eligible analyses of the data? | | N | | The article specified baseline-adjusted ANCOVA for primary and secondary endpoint changes, with no evidence that the reported ALT result was selected from multiple competing analyses. | |
|  | | **Risk of bias judgement** | | **Some concerns** | | The ALT outcome and analysis were clearly reported, but prospective finalization of the ALT analysis plan before access to outcome data could not be confirmed. | |
| **Overall bias** | | **Risk of bias judgement** | | **Some concerns** | | Overall risk of bias was judged Some concerns because prospective pre-specification of the ALT analysis could not be confirmed (Domain 5); the other domains were judged Low. | |

## S2.8 Horibe 2022

| **Unique ID** | Horibe 2022 | | **Study ID** | UMIN000020239 / jRCTs051180018 | **Assessor** | | Hanwen Zheng; Jieru Han (consensus) |
| --- | --- | --- | --- | --- | --- | --- | --- |
| **Ref/label** | Diabetes Res Clin Pract. 2022;186:109781. | | **Aim** | assignment to intervention (the 'intention-to-treat' effect) |  | |  |
| **Experimental** | Dapagliflozin + standard treatment | | **Comparator** | Standard treatment; non-SGLT2 antidiabetic therapy could be adjusted according to protocol | **Source** | | Journal article + UMIN Clinical Trials Registry (UMIN000020239) + jRCTs#051180018 + approved study protocol |
| **Outcome** | Alanine aminotransferase (ALT) | | **Result** | Change in serum ALT from baseline to week 24: control +0.1 +/- 16.6 U/L vs dapagliflozin -6.1 +/- 14.9 U/L; adjusted between-group difference -6.5 U/L (95% CI -15.4 to 2.4), P = 0.147. | **Weight** | | 1 |
| **Domain** | | **Signalling question / judgement** | | **Response** | | **Comments / justification** | |
| **Bias arising from the randomization process** | | 1.1 Was the allocation sequence random? | | Y | | Eligible participants were randomized using a web-based, password-protected system based on a computer-generated random sequence, with sex as a stratification factor. | |
|  | | 1.2 Was the allocation sequence concealed until participants were enrolled and assigned to interventions? | | Y | | Randomization was performed by the clinical research development office, independent of the investigators, after eligibility was confirmed and the patient was registered. | |
|  | | 1.3 Did baseline differences between intervention groups suggest a problem with the randomization process? | | N | | Baseline characteristics showed only isolated differences and no systematic pattern suggesting a problem with randomization. | |
|  | | **Risk of bias judgement** | | **Low** | | The sequence generation and centralized independent allocation process were appropriate and adequately concealed. | |
| **Bias due to deviations from intended interventions** | | 2.1 Were participants aware of their assigned intervention during the trial? | | Y | | The trial was open-label, so participants were aware of dapagliflozin assignment. | |
|  | | 2.2 Were carers and people delivering the interventions aware of participants' assigned intervention during the trial? | | Y | | Treating investigators implemented dapagliflozin and protocol-specified background-treatment adjustments and were aware of allocation. | |
|  | | 2.3 If Y/PY/NI to 2.1 or 2.2: Were there deviations from the intended intervention that arose because of the experimental context? | | PN | | Sulfonylurea dose reduction in the dapagliflozin group and possible addition of non-SGLT2 agents in controls were prespecified components of the treatment strategy. No important unplanned deviations were reported. | |
|  | | 2.4 If Y/PY to 2.3: Were these deviations likely to have affected the outcome? | | NA | | Not applicable because question 2.3 was answered probably no. | |
|  | | 2.5 If Y/PY/NI to 2.4: Were these deviations from intended intervention balanced between groups? | | NA | | Not applicable. | |
|  | | 2.6 Was an appropriate analysis used to estimate the effect of assignment to intervention? | | Y | | The authors explicitly stated that statistical analyses were performed on an intention-to-treat basis, and the ALT analysis retained participants in their randomized groups. | |
|  | | 2.7 If N/PN/NI to 2.6: Was there potential for a substantial impact (on the result) of the failure to analyse participants in the group to which they were randomized? | | NA | | Not applicable because question 2.6 was answered yes. | |
|  | | **Risk of bias judgement** | | **Low** | | The open-label design did not lead to identified important deviations, and an intention-to-treat analysis was used for the ALT result. | |
| **Bias due to missing outcome data** | | 3.1 Were data for this outcome available for all, or nearly all, participants randomized? | | Y | | ALT was included in the intention-to-treat analysis of 50 participants (26 dapagliflozin and 24 control), representing more than 95% of the 52 randomized participants. | |
|  | | 3.2 If N/PN/NI to 3.1: Is there evidence that result was not biased by missing outcome data? | | NA | | Not applicable because question 3.1 was answered yes. | |
|  | | 3.3 If N/PN to 3.2: Could missingness in the outcome depend on its true value? | | NA | | Not applicable. | |
|  | | 3.4 If Y/PY/NI to 3.3: Is it likely that missingness in the outcome depended on its true value? | | NA | | Not applicable. | |
|  | | **Risk of bias judgement** | | **Low** | | ALT data were available for nearly all randomized participants; the two participants without follow-up withdrew before commencing the study protocol. | |
| **Bias in measurement of the outcome** | | 4.1 Was the method of measuring the outcome inappropriate? | | N | | Serum ALT is an objective laboratory measurement and is appropriate for assessing liver enzyme change. | |
|  | | 4.2 Could measurement or ascertainment of the outcome have differed between intervention groups? | | N | | The same scheduled laboratory assessments were applied to both randomized groups. | |
|  | | 4.3 Were outcome assessors aware of the intervention received by study participants? | | PN | | The paper does not specifically state that laboratory personnel were blinded; however, test results were not revealed to investigators until the database was locked except in emergencies. | |
|  | | 4.4 If Y/PY/NI to 4.3: Could assessment of the outcome have been influenced by knowledge of intervention received? | | PN | | Even if an assessor had access to allocation information, the numerical ALT concentration from standard laboratory testing would be unlikely to be influenced by that knowledge. | |
|  | | 4.5 If Y/PY/NI to 4.4: Is it likely that assessment of the outcome was influenced by knowledge of intervention received? | | NA | | Not applicable because question 4.4 was answered probably no. | |
|  | | **Risk of bias judgement** | | **Low** | | ALT was objectively measured using standard laboratory testing, with results withheld from investigators until database lock; measurement bias was unlikely. | |
| **Bias in selection of the reported result** | | 5.1 Were the data that produced this result analysed in accordance with a pre-specified analysis plan that was finalized before unblinded outcome data were available for analysis? | | Y | | The trial was prospectively registered, original and amended protocols were approved, ALT was listed among the prespecified secondary outcomes, and analyses were performed by independent statistical analysts after database lock. | |
|  | | 5.2 ... multiple eligible outcome measurements (e.g. scales, definitions, time points) within the outcome domain? | | N | | ALT was measured according to the prespecified study schedule, including baseline and week 24, with no competing eligible ALT definition reported for this result. | |
|  | | 5.3 ... multiple eligible analyses of the data? | | N | | Secondary outcomes were analysed using prespecified sex-adjusted ANCOVA or Wilcoxon rank-sum testing according to distribution, with no evidence of selecting among multiple analyses based on the result. | |
|  | | **Risk of bias judgement** | | **Low** | | Prospective registration, protocol documentation, prespecified ALT assessment, database lock, and independent analysis support low risk of selective reporting. | |
| **Overall bias** | | **Risk of bias judgement** | | **Low** | | Overall risk of bias for the ALT result was judged Low: all five RoB 2 domains were assessed as Low. | |

## S2.9 Zhang 2020

| **Unique ID** | Zhang 2020 | | **Study ID** | Not reported | **Assessor** | | Hanwen Zheng; Jieru Han (consensus) |
| --- | --- | --- | --- | --- | --- | --- | --- |
| **Ref/label** | Clin Res Hepatol Gastroenterol. 2020;44(5):674–680. | | **Aim** | assignment to intervention (the 'intention-to-treat' effect) |  | |  |
| **Experimental** | Liraglutide 0.6 mg/d (week 1) titrated to 1.2 mg/d (weeks 2–24), added to metformin + diet/exercise advice | | **Comparator** | Pioglitazone 15 mg/d (week 1) titrated to 30 mg/d (weeks 2–24), added to metformin + diet/exercise advice | **Source** | | Journal article; no trial registration or separate protocol/statistical analysis plan was reported |
| **Outcome** | Alanine aminotransferase (ALT) | | **Result** | Serum ALT (natural-log transformed) from baseline to week 24: liraglutide 3.4 +/- 0.3 to 3.0 +/- 0.4 vs pioglitazone 3.6 +/- 0.5 to 3.6 +/- 0.3; the report states no significant treatment effect on liver enzymes. | **Weight** | | 1 |
| **Domain** | | **Signalling question / judgement** | | **Response** | | **Comments / justification** | |
| **Bias arising from the randomization process** | | 1.1 Was the allocation sequence random? | | Y | | Participants were randomized 1:1 using a computer-generated sequence. | |
|  | | 1.2 Was the allocation sequence concealed until participants were enrolled and assigned to interventions? | | NI | | The report did not describe central randomization, sealed envelopes, pharmacy-controlled concealment, or another method ensuring that the allocation sequence was concealed before assignment. | |
|  | | 1.3 Did baseline differences between intervention groups suggest a problem with the randomization process? | | N | | Reported baseline clinical characteristics were broadly comparable between the liraglutide and pioglitazone groups, with no pattern suggesting failure of randomization. | |
|  | | **Risk of bias judgement** | | **Some concerns** | | The allocation sequence was random, but the method used to conceal assignment before enrolment was not reported. | |
| **Bias due to deviations from intended interventions** | | 2.1 Were participants aware of their assigned intervention during the trial? | | Y | | The abstract and Methods describe the study as open-label. The same Methods sentence also uses the term double-blind, but no double-dummy or masking procedure is described; participants were therefore considered aware of treatment assignment. | |
|  | | 2.2 Were carers and people delivering the interventions aware of participants' assigned intervention during the trial? | | PY | | Because liraglutide was administered by injection and pioglitazone orally, and no masking procedure was described, personnel delivering the interventions were probably aware of allocation. | |
|  | | 2.3 If Y/PY/NI to 2.1 or 2.2: Were there deviations from the intended intervention that arose because of the experimental context? | | PN | | Both groups continued the same metformin regimen and received the same diet and exercise advice. No crossover, unplanned co-intervention, or other trial-context deviation was reported. | |
|  | | 2.4 If Y/PY to 2.3: Were these deviations likely to have affected the outcome? | | NA | | Not applicable because question 2.3 was answered probably no. | |
|  | | 2.5 If Y/PY/NI to 2.4: Were these deviations from intended intervention balanced between groups? | | NA | | Not applicable. | |
|  | | 2.6 Was an appropriate analysis used to estimate the effect of assignment to intervention? | | PY | | The results continued to report 30 participants in each randomized group at week 24, with no reported treatment switching or analysis according to treatment received. | |
|  | | 2.7 If N/PN/NI to 2.6: Was there potential for a substantial impact (on the result) of the failure to analyse participants in the group to which they were randomized? | | NA | | Not applicable because question 2.6 was answered probably yes. | |
|  | | **Risk of bias judgement** | | **Low** | | Despite the open-label design, no important deviations from intended intervention were identified, and the analysis appeared to preserve randomized treatment groups. | |
| **Bias due to missing outcome data** | | 3.1 Were data for this outcome available for all, or nearly all, participants randomized? | | PY | | All 60 randomized participants continued to be represented as 30 participants per group in the reported week-24 tables, and no withdrawals or missing ALT measurements were described. | |
|  | | 3.2 If N/PN/NI to 3.1: Is there evidence that result was not biased by missing outcome data? | | NA | | Not applicable because question 3.1 was answered probably yes. | |
|  | | 3.3 If N/PN to 3.2: Could missingness in the outcome depend on its true value? | | NA | | Not applicable. | |
|  | | 3.4 If Y/PY/NI to 3.3: Is it likely that missingness in the outcome depended on its true value? | | NA | | Not applicable. | |
|  | | **Risk of bias judgement** | | **Low** | | The report gives no indication of missing ALT outcome data and continues to report the full randomized sample at week 24. | |
| **Bias in measurement of the outcome** | | 4.1 Was the method of measuring the outcome inappropriate? | | N | | ALT was measured using an ARCHITECT C8000 blood chemistry analyzer, an appropriate objective laboratory method. | |
|  | | 4.2 Could measurement or ascertainment of the outcome have differed between intervention groups? | | N | | The same laboratory method and the same baseline and week-24 schedule were used for both treatment groups. | |
|  | | 4.3 Were outcome assessors aware of the intervention received by study participants? | | NI | | The report did not state whether laboratory personnel measuring ALT were blinded to treatment assignment. | |
|  | | 4.4 If Y/PY/NI to 4.3: Could assessment of the outcome have been influenced by knowledge of intervention received? | | PN | | ALT was generated by an automated blood chemistry analyzer, so knowledge of treatment allocation would be unlikely to materially influence the measured value. | |
|  | | 4.5 If Y/PY/NI to 4.4: Is it likely that assessment of the outcome was influenced by knowledge of intervention received? | | NA | | Not applicable because question 4.4 was answered probably no. | |
|  | | **Risk of bias judgement** | | **Low** | | ALT was an objective automated laboratory measurement applied consistently across groups; lack of reported laboratory blinding was unlikely to bias the value. | |
| **Bias in selection of the reported result** | | 5.1 Were the data that produced this result analysed in accordance with a pre-specified analysis plan that was finalized before unblinded outcome data were available for analysis? | | NI | | The published Methods identify liver enzymes as efficacy outcomes and describe the statistical analysis, but no prospective trial registration, protocol, or dated statistical analysis plan was reported to confirm that the ALT analysis was finalized before outcome data were available. | |
|  | | 5.2 ... multiple eligible outcome measurements (e.g. scales, definitions, time points) within the outcome domain? | | N | | ALT was measured using one defined laboratory method at baseline and week 24; no alternative eligible ALT measurement or final time point was reported. | |
|  | | 5.3 ... multiple eligible analyses of the data? | | N | | The report specified log transformation for ALT and the planned group/time comparisons, with no evidence of selecting from multiple alternative ALT analyses. | |
|  | | **Risk of bias judgement** | | **Some concerns** | | The reported ALT analysis is internally coherent, but prospective pre-specification before access to outcome data could not be verified. | |
| **Overall bias** | | **Risk of bias judgement** | | **Some concerns** | | Overall risk of bias was judged Some concerns because allocation concealment and prospective pre-specification of the ALT analysis were not adequately documented (Domains 1 and 5). | |

## S2.10 Shimizu 2019

| **Unique ID** | Shimizu 2019 | | **Study ID** | UMIN000022155 | **Assessor** | | Hanwen Zheng; Jieru Han (consensus) |
| --- | --- | --- | --- | --- | --- | --- | --- |
| **Ref/label** | Diabetes Obes Metab. 2019;21(2):285–292. | | **Aim** | Effect of assignment to intervention (intention-to-treat estimand) |  | |  |
| **Experimental** | Dapagliflozin 5 mg qd + standard diabetes care, 24 weeks | | **Comparator** | Standard diabetes care (no SGLT2i), 24 weeks | **Source** | | Journal article; UMIN Clinical Trials Registry (UMIN000022155) |
| **Outcome** | Alanine aminotransferase (ALT) | | **Result** | Change in serum ALT from baseline to week 24: dapagliflozin 38.0 (21.5–61.0) to 26.5 (16.3–42.5) U/L vs standard-treatment control 33.0 (24.5–46.5) to 32.0 (25.0–49.3) U/L; between-group P = 0.0212. | **Weight** | | 1 |
| **Domain** | | **Signalling question / judgement** | | **Response** | | **Comments / justification** | |
| **Bias arising from the randomization process** | | 1.1 Was the allocation sequence random? | | PY | | Participants were allocated 1:1 using the Pocock–Simon minimization method, stratified by sex, age, and BMI. This was considered probably random for the RoB 2 judgement. | |
|  | | 1.2 Was the allocation sequence concealed until participants were enrolled and assigned to interventions? | | PY | | The report states that patients were randomly allocated after eligibility/enrolment using the prespecified minimization procedure. A separate concealment mechanism was not described, so concealment was judged probably, rather than definitely, adequate. | |
|  | | 1.3 Did baseline differences between intervention groups suggest a problem with the randomization process? | | N | | Baseline ALT and the great majority of demographic and laboratory variables were comparable between groups. The isolated difference in type IV collagen 7S was not considered a systematic pattern suggesting failure of randomization. | |
|  | | **Risk of bias judgement** | | **Low** | | Randomization used a prespecified covariate-adaptive minimization procedure and no systematic baseline imbalance relevant to ALT was identified. Allocation concealment was judged probably adequate; therefore this domain was judged Low. | |
| **Bias due to deviations from intended interventions** | | 2.1 Were participants aware of their assigned intervention during the trial? | | Y | | The study used an open-label design, so participants were aware of the assigned treatment. | |
|  | | 2.2 Were carers and people delivering the interventions aware of participants' assigned intervention during the trial? | | Y | | Treating clinicians were aware of allocation because dapagliflozin was added only in the intervention group and standard diabetes treatment was managed clinically. | |
|  | | 2.3 If Y/PY/NI to 2.1 or 2.2: Were there deviations from the intended intervention that arose because of the experimental context? | | N | | No important trial-context deviation from the intended strategies was identified. Intensification of non-SGLT2 glucose-lowering therapy in the control group was explicitly part of the intended standard-treatment strategy for achieving glycaemic targets rather than an unintended deviation caused by awareness of allocation. | |
|  | | 2.4 If Y/PY to 2.3: Were these deviations likely to have affected the outcome? | | NA | | Not applicable because no important deviation from the intended intervention arising because of trial context was identified. | |
|  | | 2.5 If Y/PY/NI to 2.4: Were these deviations from intended intervention balanced between groups? | | NA | | Not applicable because question 2.4 was not required. | |
|  | | 2.6 Was an appropriate analysis used to estimate the effect of assignment to intervention? | | Y | | Participants with available ALT data were analysed according to their randomized treatment groups. Missing outcome data are considered separately in Domain 3. | |
|  | | 2.7 If N/PN/NI to 2.6: Was there potential for a substantial impact (on the result) of the failure to analyse participants in the group to which they were randomized? | | NA | | Not applicable because the analysis retained participants in their randomized groups. | |
|  | | **Risk of bias judgement** | | **Low** | | Although the trial was open-label, knowledge of treatment assignment was not shown to cause important deviations from the intended intervention strategies. Protocol-permitted treatment intensification formed part of the intended comparator strategy, and observed participants were analysed by randomized group. | |
| **Bias due to missing outcome data** | | 3.1 Were data for this outcome available for all, or nearly all, participants randomized? | | PN | | ALT follow-up data were available for 57 of 63 randomized participants (90.5%): 33/35 in the dapagliflozin group and 24/28 in the control group. This was judged probably not 'all or nearly all' for a strict outcome-specific assessment. | |
|  | | 3.2 If N/PN/NI to 3.1: Is there evidence that result was not biased by missing outcome data? | | PY | | The six non-completions were explained (congestive heart failure, Charcot-joint surgery, no longer meeting criteria, and loss to follow-up). Most reasons were not plausibly related to the participant's unobserved ALT value, and the amount of missing data was modest; therefore the ALT result was judged probably not materially biased by missingness. | |
|  | | 3.3 If N/PN to 3.2: Could missingness in the outcome depend on its true value? | | NA | | Not applicable because question 3.2 was answered Probably yes. | |
|  | | 3.4 If Y/PY/NI to 3.3: Is it likely that missingness in the outcome depended on its true value? | | NA | | Not applicable because question 3.3 was not required. | |
|  | | **Risk of bias judgement** | | **Low** | | Six of 63 randomized participants lacked follow-up data, but reasons were reported and were predominantly unrelated to ALT. Missingness was therefore judged unlikely to materially bias the ALT comparison. | |
| **Bias in measurement of the outcome** | | 4.1 Was the method of measuring the outcome inappropriate? | | N | | Serum ALT is an objective laboratory measurement and the method of measuring this outcome was appropriate. | |
|  | | 4.2 Could measurement or ascertainment of the outcome have differed between intervention groups? | | N | | There was no indication that ALT ascertainment or laboratory procedures differed between randomized groups. | |
|  | | 4.3 Were outcome assessors aware of the intervention received by study participants? | | NI | | Blinding of laboratory personnel specifically measuring ALT was not reported. | |
|  | | 4.4 If Y/PY/NI to 4.3: Could assessment of the outcome have been influenced by knowledge of intervention received? | | PN | | Even if laboratory personnel were aware of allocation, the numerical ALT concentration obtained using routine laboratory testing is unlikely to be materially influenced by such knowledge. | |
|  | | 4.5 If Y/PY/NI to 4.4: Is it likely that assessment of the outcome was influenced by knowledge of intervention received? | | NA | | Not applicable because assessment of an objective laboratory ALT concentration was judged unlikely to be influenced by knowledge of treatment assignment. | |
|  | | **Risk of bias judgement** | | **Low** | | ALT was measured as an objective laboratory outcome using the same clinical laboratory framework in both groups. Lack of explicit laboratory blinding was unlikely to influence the measured ALT concentration. | |
| **Bias in selection of the reported result** | | 5.1 Were the data that produced this result analysed in accordance with a pre-specified analysis plan that was finalized before unblinded outcome data were available for analysis? | | PY | | The trial was prospectively registered (UMIN000022155), and the article's Methods defined change in liver enzymes, including ALT, from baseline to week 24 as a secondary endpoint before presenting the results. In the absence of evidence of post hoc alteration, the reported ALT analysis was judged probably prespecified. | |
|  | | 5.2 ... multiple eligible outcome measurements (e.g. scales, definitions, time points) within the outcome domain? | | N | | ALT was reported for the fixed 24-week endpoint using one conventional laboratory definition; no alternative eligible ALT measurement scale or time point was described for this result. | |
|  | | 5.3 ... multiple eligible analyses of the data? | | N | | The report used the stated parametric/non-parametric between-group approach appropriate to the data and did not present multiple competing ALT analyses from which the reported result appeared to have been selected. | |
|  | | **Risk of bias judgement** | | **Low** | | ALT was an explicitly defined secondary endpoint at 24 weeks, the trial was prospectively registered, and there was no evidence that the reported ALT result was selected from multiple measurements or analyses. | |
| **Overall bias** | | **Risk of bias judgement** | | **Low** | | All five RoB 2 domains were judged Low for the ALT result. The trial was open-label, but open-label status alone was not treated as a source of bias: no important trial-context deviation from the intended strategies was identified, ALT is an objective laboratory outcome, missing data were modest with reported reasons, and the 24-week ALT endpoint was explicitly described as a secondary outcome. | |

## S2.11 Phrueksotsai 2021

| **Unique ID** | Phrueksotsai 2021 | | **Study ID** | TCTR20170511001 | **Assessor** | | Hanwen Zheng; Jieru Han (consensus) |
| --- | --- | --- | --- | --- | --- | --- | --- |
| **Ref/label** | J Gastroenterol Hepatol. 2021;36(10):2952–2959. | | **Aim** | Effect of assignment to intervention (intention-to-treat estimand) |  | |  |
| **Experimental** | Dapagliflozin 10 mg qd + stable OADs, 12 weeks | | **Comparator** | Placebo + stable OADs, 12 weeks | **Source** | | Journal article; trial registration TCTR20170511001 |
| **Outcome** | Alanine aminotransferase (ALT) | | **Result** | Change in serum ALT from baseline to week 12: the dapagliflozin group showed a significantly greater ALT reduction than the placebo group (between-group P < 0.05). | **Weight** | | 1 |
| **Domain** | | **Signalling question / judgement** | | **Response** | | **Comments / justification** | |
| **Bias arising from the randomization process** | | 1.1 Was the allocation sequence random? | | Y | | Participants were randomized 1:1 using a computer-generated block randomization procedure. | |
|  | | 1.2 Was the allocation sequence concealed until participants were enrolled and assigned to interventions? | | PY | | A separate allocation-concealment procedure was not described in detail, but the double-blind placebo-controlled design and use of a computer-generated allocation schedule make prior knowledge of the next assignment unlikely; concealment was therefore judged probably adequate. | |
|  | | 1.3 Did baseline differences between intervention groups suggest a problem with the randomization process? | | N | | Baseline demographic and laboratory findings were reported as similar between groups, with no pattern suggesting a problem with randomization. | |
|  | | **Risk of bias judgement** | | **Low** | | The computer-generated randomized allocation, probably adequate concealment, and balanced baseline characteristics supported a Low risk judgement. | |
| **Bias due to deviations from intended interventions** | | 2.1 Were participants aware of their assigned intervention during the trial? | | N | | The study was double-blinded and placebo-controlled; participants were not expected to know whether they received dapagliflozin or placebo. | |
|  | | 2.2 Were carers and people delivering the interventions aware of participants' assigned intervention during the trial? | | N | | Investigators/treating personnel were blinded to treatment assignment in the placebo-controlled design. | |
|  | | 2.3 If Y/PY/NI to 2.1 or 2.2: Were there deviations from the intended intervention that arose because of the experimental context? | | NA | | Not applicable because participants and people delivering the intervention were judged not to be aware of treatment assignment. | |
|  | | 2.4 If Y/PY to 2.3: Were these deviations likely to have affected the outcome? | | NA | | Not applicable because question 2.3 was not required. | |
|  | | 2.5 If Y/PY/NI to 2.4: Were these deviations from intended intervention balanced between groups? | | NA | | Not applicable because question 2.4 was not required. | |
|  | | 2.6 Was an appropriate analysis used to estimate the effect of assignment to intervention? | | Y | | Participants with available ALT data were compared in their randomized groups; there was no indication that participants were reclassified according to treatment received. | |
|  | | 2.7 If N/PN/NI to 2.6: Was there potential for a substantial impact (on the result) of the failure to analyse participants in the group to which they were randomized? | | NA | | Not applicable because the analysis retained the randomized-group comparison. | |
|  | | **Risk of bias judgement** | | **Low** | | The double-blind placebo-controlled design minimized awareness-related deviations, and the ALT comparison preserved randomized treatment groups. | |
| **Bias due to missing outcome data** | | 3.1 Were data for this outcome available for all, or nearly all, participants randomized? | | PN | | Thirty-eight of 40 randomized participants (95%) completed the 12-week study (18/20 dapagliflozin; 20/20 placebo), so outcome data were available for nearly all but not literally all randomized participants. | |
|  | | 3.2 If N/PN/NI to 3.1: Is there evidence that result was not biased by missing outcome data? | | PY | | Only two participants lacked follow-up, both in the dapagliflozin group. The small amount of missing data was judged unlikely to materially alter the ALT comparison, with no evidence that loss to follow-up depended on the unobserved ALT value. | |
|  | | 3.3 If N/PN to 3.2: Could missingness in the outcome depend on its true value? | | NA | | Not applicable because question 3.2 was answered Probably yes. | |
|  | | 3.4 If Y/PY/NI to 3.3: Is it likely that missingness in the outcome depended on its true value? | | NA | | Not applicable because question 3.3 was not required. | |
|  | | **Risk of bias judgement** | | **Low** | | ALT outcome data were available for 95% of randomized participants and the small amount of missing data was unlikely to materially bias the result. | |
| **Bias in measurement of the outcome** | | 4.1 Was the method of measuring the outcome inappropriate? | | N | | Serum ALT is an objective laboratory outcome and is an appropriate measure of liver biochemistry. | |
|  | | 4.2 Could measurement or ascertainment of the outcome have differed between intervention groups? | | N | | The same laboratory outcome and assessment schedule were used in both randomized groups; there was no indication of differential ascertainment. | |
|  | | 4.3 Were outcome assessors aware of the intervention received by study participants? | | PN | | Because the trial was double-blinded, personnel involved in outcome ascertainment were probably unaware of treatment allocation. | |
|  | | 4.4 If Y/PY/NI to 4.3: Could assessment of the outcome have been influenced by knowledge of intervention received? | | NA | | Not applicable because outcome assessors were judged probably unaware of intervention assignment. | |
|  | | 4.5 If Y/PY/NI to 4.4: Is it likely that assessment of the outcome was influenced by knowledge of intervention received? | | NA | | Not applicable because question 4.4 was not required. | |
|  | | **Risk of bias judgement** | | **Low** | | ALT was an objective laboratory measure assessed comparably between groups in a double-blind placebo-controlled trial. | |
| **Bias in selection of the reported result** | | 5.1 Were the data that produced this result analysed in accordance with a pre-specified analysis plan that was finalized before unblinded outcome data were available for analysis? | | PY | | The study was prospectively registered as TCTR20170511001 and the study aim and Methods included liver-related biochemical markers, including ALT, assessed at the fixed 12-week endpoint. With no evidence of post hoc alteration, the reported ALT result was judged probably prespecified. | |
|  | | 5.2 ... multiple eligible outcome measurements (e.g. scales, definitions, time points) within the outcome domain? | | N | | ALT was evaluated at the fixed baseline and week-12 time points using the same laboratory outcome definition; no multiple eligible ALT measurements were reported for this result. | |
|  | | 5.3 ... multiple eligible analyses of the data? | | N | | No multiple competing statistical analyses of the ALT outcome were reported from which the presented result appeared to have been selected. | |
|  | | **Risk of bias judgement** | | **Low** | | Prospective registration, a fixed 12-week endpoint, and the absence of evidence for selective choice among multiple ALT measurements or analyses supported a Low risk judgement. | |
| **Overall bias** | | **Risk of bias judgement** | | **Low** | | All five RoB 2 domains were judged Low for the ALT result. The randomized double-blind placebo-controlled design reduced the risk of deviations and measurement bias, follow-up was 95%, and ALT was assessed as a fixed biochemical outcome. | |

# Supplementary Table S3. Detailed RoB 2 assessments for glycated hemoglobin (HbA1c)

**Target estimand:** effect of assignment to intervention (intention-to-treat). **Response codes:** Y, yes; PY, probably yes; PN, probably no; N, no; NI, no information; NA, not applicable.

## S3.1 Ito 2017

| **Unique ID** | Ito 2017 | | **Study ID** | UMIN000022651 | **Assessor** | | Hanwen Zheng; Jieru Han (consensus) |
| --- | --- | --- | --- | --- | --- | --- | --- |
| **Ref/label** | Diabetes Care. 2017;40(10):1364–1372. | | **Aim** | Effect of assignment to intervention |  | |  |
| **Experimental** | Ipragliflozin | | **Comparator** | Pioglitazone | **Source** | | https://doi.org/10.2337/dc17-0518 |
| **Outcome** | Glycated hemoglobin (HbA1c) | | **Result** | Change from baseline in HbA1c at week 24 (ipragliflozin vs pioglitazone). | **Weight** | | 1 |
| **Domain** | | **Signalling question / judgement** | | **Response** | | **Comments / justification** | |
| **Bias arising from the randomization process** | | 1.1 Was the allocation sequence random? | | Y | | Eligible participants were assigned 1:1 using a computer-generated randomization sequence (p. 2). | |
|  | | 1.2 Was the allocation sequence concealed until participants were enrolled and assigned to interventions? | | NI | | The report describes a computer-generated sequence but does not state how allocation was concealed or who implemented the sequence. Allocation concealment therefore cannot be verified from the supplied article. | |
|  | | 1.3 Did baseline differences between intervention groups suggest a problem with the randomization process? | | PN | | Baseline characteristics were generally comparable between randomized groups, including glycemic measures where reported. No systematic pattern of imbalance suggested a problem with the randomization process. | |
|  | | **Risk of bias judgement** | | **Some concerns** | | The sequence was random, but allocation concealment was not reported. The observed baseline imbalances do not clearly establish a randomization problem. | |
| **Bias due to deviations from intended interventions** | | 2.1 Were participants aware of their assigned intervention during the trial? | | Y | | The trial was explicitly described as open-label; participants therefore knew which active drug they received (pp. 1–2). | |
|  | | 2.2 Were carers and people delivering the interventions aware of participants' assigned intervention during the trial? | | Y | | Because this was an open-label active-controlled trial with different drugs and dosing procedures, treating clinicians and study personnel were aware of treatment assignment (p. 2). | |
|  | | 2.3 If Y/PY/NI to 2.1 or 2.2: Were there deviations from the intended intervention that arose because of the experimental context? | | PN | | No additions or adjustments to background antidiabetes, antihypertensive, or lipid-lowering therapy were permitted, apart from protocol-allowed reduction/discontinuation of insulin or sulfonylurea to avoid hypoglycemia. No sulfonylurea reductions occurred, and insulin-dose changes did not differ significantly between groups. Diet and exercise counselling was applied to both groups (pp. 2 and 4). | |
|  | | 2.4 If Y/PY to 2.3: Were these deviations likely to have affected the outcome? | | NA | | Not applicable because no important trial-context-related deviations from the intended interventions were identified. | |
|  | | 2.5 If Y/PY/NI to 2.4: Were these deviations from intended intervention balanced between groups? | | NA | | Not applicable because no important deviations requiring assessment of balance were identified. | |
|  | | 2.6 Was an appropriate analysis used to estimate the effect of assignment to intervention? | | Y | | The authors explicitly stated that statistical analyses were performed in an intention-to-treat population. HbA1c was a prespecified key secondary outcome, and Table 2 reports HbA1c results using the randomized group denominators (ipragliflozin n=32; pioglitazone n=34) (pp. 3-4). | |
|  | | 2.7 If N/PN/NI to 2.6: Was there potential for a substantial impact of failure to analyse participants in the group to which they were randomized? | | NA | | Not applicable because an intention-to-treat analysis by randomized group was reported. | |
|  | | **Risk of bias judgement** | | **Low** | | Although the trial was open-label, no important deviations arising from the trial context were identified, co-interventions were largely controlled, and an intention-to-treat analysis was reported. | |
| **Bias due to missing outcome data** | | 3.1 Were data for this outcome available for all, or nearly all, participants randomized? | | PN | | Sixty-one of 66 randomized participants completed the 24-week trial (30/32 ipragliflozin; 31/34 pioglitazone). The report does not state whether observed week-24 HbA1c measurements were available for every randomized participant, although Table 2 retains the original randomized denominators. Complete HbA1c data therefore cannot be confirmed (p. 4). | |
|  | | 3.2 If N/PN/NI to 3.1: Is there evidence that the result was not biased by missing outcome data? | | NI | | Although the analyses were described as intention-to-treat and Table 2 retained the randomized denominators, the report does not explain how missing week-24 HbA1c measurements for participants who discontinued were handled and does not provide an HbA1c-specific missing-data sensitivity analysis. | |
|  | | 3.3 If N/PN to 3.2: Could missingness in the outcome have depended on its true value? | | PN | | Discontinuations were relatively balanced (2/32 vs 3/34). Reasons were voluntary withdrawal and loss to follow-up in the ipragliflozin group, and two edema-related adverse events plus one loss to follow-up in the pioglitazone group. These reasons were not clearly related to the unobserved true HbA1c value, although a relationship cannot be completely excluded (p. 4). | |
|  | | 3.4 If Y/PY/NI to 3.3: Is it likely that missingness in the outcome depended on its true value? | | NA | | Not applicable because missingness was judged probably not to depend on the true HbA1c value. | |
|  | | **Risk of bias judgement** | | **Low** | | The amount of missing follow-up was modest and relatively balanced, and the reported reasons were unlikely to depend on the unobserved HbA1c value. The method used to handle missing HbA1c measurements was not fully described. | |
| **Bias in measurement of the outcome** | | 4.1 Was the method of measuring the outcome inappropriate? | | N | | HbA1c is a standard objective laboratory biomarker of glycemic control; the method of measurement was appropriate for the outcome. | |
|  | | 4.2 Could measurement or ascertainment of the outcome have differed between intervention groups? | | N | | HbA1c was measured using the same clinical laboratory approach and follow-up schedule across randomized groups; no differential ascertainment procedure was reported. | |
|  | | 4.3 Were outcome assessors aware of the intervention received by study participants? | | NI | | Blinding of the laboratory personnel responsible for HbA1c measurement was not specifically reported. | |
|  | | 4.4 If Y/PY/NI to 4.3: Could assessment of the outcome have been influenced by knowledge of intervention received? | | N | | HbA1c is an objective laboratory measurement; knowledge of treatment allocation would not be expected to materially influence the measured value. | |
|  | | 4.5 If Y/PY/NI to 4.4: Is it likely that assessment of the outcome was influenced by knowledge of intervention received? | | NA | | Not applicable because assessment of HbA1c was judged not to be influenced by knowledge of the intervention. | |
|  | | **Risk of bias judgement** | | **Low** | | HbA1c was measured objectively using comparable laboratory procedures across groups; unreported assessor blinding was unlikely to introduce meaningful measurement bias. | |
| **Bias in selection of the reported result** | | 5.1 Were the data that produced this result analysed in accordance with a pre-specified analysis plan finalized before unblinded outcome data were available for analysis? | | PY | | The trial was prospectively registered (UMIN000022651) and HbA1c was reported at the fixed 24-week end-of-treatment assessment. Although a standalone dated HbA1c statistical analysis plan was not available, the prospective trial framework, fixed assessment time point, and absence of evidence for result-driven alternative HbA1c analyses were considered sufficient to judge prespecification as probable. | |
|  | | 5.2 Was the numerical result being assessed likely to have been selected, on the basis of the results, from multiple eligible outcome measurements within the outcome domain? | | PN | | HbA1c was assessed at the fixed 24-week follow-up, making selective choice among multiple eligible time points or definitions unlikely. | |
|  | | 5.3 Was the numerical result being assessed likely to have been selected, on the basis of the results, from multiple eligible analyses of the data? | | PN | | The report used a conventional between-group analysis of change in HbA1c, with no indication that the reported result was selected from multiple competing analyses on the basis of the findings. | |
|  | | **Risk of bias judgement** | | **Low** | | HbA1c was assessed at a fixed planned follow-up, and there was no indication that the reported value was selected from multiple eligible measurements or statistical analyses on the basis of the findings. Considering the prospective trial registration and the objective, standard nature of HbA1c, the risk of selective reporting was judged Low. | |
| **Overall bias** | | **Risk of bias judgement** | | **Some concerns** | | Overall risk of bias for the HbA1c result was judged Some concerns. The main remaining uncertainty related to allocation concealment in the randomization process; the other four domains were judged Low. | |

## S3.2 Kinoshita 2020

| **Unique ID** | Kinoshita 2020 | | **Study ID** | UMIN000021291 | **Assessor** | | Hanwen Zheng; Jieru Han (consensus) |
| --- | --- | --- | --- | --- | --- | --- | --- |
| **Ref/label** | J Diabetes Investig. 2020;11(6):1612–1622. | | **Aim** | Effect of assignment to intervention |  | |  |
| **Experimental** | Dapagliflozin | | **Comparator** | Pioglitazone | **Source** | | https://doi.org/10.1111/jdi.13279 |
| **Outcome** | Glycated hemoglobin (HbA1c) | | **Result** | Change from baseline in HbA1c at week 28 (dapagliflozin vs pioglitazone). | **Weight** | | 1 |
| **Domain** | | **Signalling question / judgement** | | **Response** | | **Comments / justification** | |
| **Bias arising from the randomization process** | | 1.1 Was the allocation sequence random? | | Y | | A predefined computer-generated block randomization table with a 1:1:1 allocation ratio was used; the random sequence was generated independently using computer software. | |
|  | | 1.2 Was the allocation sequence concealed until participants were enrolled and assigned to interventions? | | PY | | The allocation sequence was generated independently, and enrollment/assignment procedures were separated from treatment and data collection. Although the specific concealment mechanism was not fully described, allocation was probably adequately concealed. | |
|  | | 1.3 Did baseline differences between intervention groups suggest a problem with the randomization process? | | N | | Baseline characteristics were generally comparable between randomized groups, including glycemic measures where reported. No systematic pattern of imbalance suggested a problem with the randomization process. | |
|  | | **Risk of bias judgement** | | **Low** | | The random sequence was appropriately generated, allocation was probably adequately concealed, and baseline characteristics provided no evidence of a randomization problem. | |
| **Bias due to deviations from intended interventions** | | 2.1 Were participants aware of their assigned intervention during the trial? | | Y | | The trial was explicitly described as open-label. | |
|  | | 2.2 Were carers and people delivering the interventions aware of participants’ assigned intervention during the trial? | | Y | | Because the trial was open-label, treating clinicians were aware of the assigned intervention. | |
|  | | 2.3 If Y/PY/NI to 2.1 or 2.2: Were there deviations from the intended intervention that arose because of the experimental context? | | PN | | Concomitant antidiabetic medications were intended to remain unchanged, and treatment guidance and dose adjustment followed the study protocol. No important differential deviations attributable to the open-label trial context were reported. | |
|  | | 2.4 If Y/PY to 2.3: Were these deviations likely to have affected the outcome? | | NA | | Not applicable because no important deviations arising from the experimental context were identified. | |
|  | | 2.5 If Y/PY/NI to 2.4: Were these deviations from intended intervention balanced between groups? | | NA | | Not applicable. | |
|  | | 2.6 Was an appropriate analysis used to estimate the effect of assignment to intervention? | | PY | | Participants with available outcome data were analysed according to their randomized treatment groups, with no indication of treatment-group reclassification. Missing outcome data were considered separately in Domain 3. | |
|  | | 2.7 If N/PN/NI to 2.6: Was there potential for a substantial impact on the result of the failure to analyse participants in the group to which they were randomized? | | NA | | Not applicable because the analysis was considered appropriate for estimating the effect of assignment to intervention. | |
|  | | **Risk of bias judgement** | | **Low** | | Although the trial was open-label, no important trial-context-related deviations were identified. Participants were analysed according to randomized assignment, while missing outcome data were addressed separately in Domain 3. | |
| **Bias due to missing outcome data** | | 3.1 Were data for this outcome available for all, or nearly all, participants randomized? | | N | | For the dapagliflozin-versus-pioglitazone comparison, not all randomized participants were included in the final analysis (32/40 dapagliflozin and 33/36 pioglitazone). Outcome-specific completeness of HbA1c data for every randomized participant could therefore not be confirmed. | |
|  | | 3.2 If N/PN/NI to 3.1: Is there evidence that the result was not biased by missing outcome data? | | N | | No formal sensitivity analysis specifically addressing missing HbA1c data was reported. | |
|  | | 3.3 If N/PN to 3.2: Could missingness in the outcome depend on its true value? | | PN | | Reported reasons for unavailable follow-up data were related to follow-up, withdrawal, or availability of follow-up measurements rather than to the unobserved HbA1c value; dependence on the true HbA1c value was judged probably absent. | |
|  | | 3.4 If Y/PY/NI to 3.3: Is it likely that missingness in the outcome depended on its true value? | | NA | | Not applicable because missingness was judged probably not to depend on the true HbA1c value. | |
|  | | **Risk of bias judgement** | | **Low** | | Although not all randomized participants contributed to the final analysis, the reported reasons for missingness did not indicate dependence on the unobserved HbA1c value; substantial bias from missing outcome data was considered unlikely. | |
| **Bias in measurement of the outcome** | | 4.1 Was the method of measuring the outcome inappropriate? | | N | | HbA1c is a standard objective laboratory biomarker of glycemic control; the method of measurement was appropriate for the outcome. | |
|  | | 4.2 Could measurement or ascertainment of the outcome have differed between intervention groups? | | N | | HbA1c was measured using the same clinical laboratory approach and follow-up schedule across randomized groups; no differential ascertainment procedure was reported. | |
|  | | 4.3 Were outcome assessors aware of the intervention received by study participants? | | NI | | Blinding of the laboratory personnel responsible for HbA1c measurement was not specifically reported. | |
|  | | 4.4 If Y/PY/NI to 4.3: Could assessment of the outcome have been influenced by knowledge of intervention received? | | N | | HbA1c is an objective laboratory measurement; knowledge of treatment allocation would not be expected to materially influence the measured value. | |
|  | | 4.5 If Y/PY/NI to 4.4: Is it likely that assessment of the outcome was influenced by knowledge of intervention received? | | NA | | Not applicable because assessment of HbA1c was judged not to be influenced by knowledge of the intervention. | |
|  | | **Risk of bias judgement** | | **Low** | | HbA1c was measured objectively using comparable laboratory procedures across groups; unreported assessor blinding was unlikely to introduce meaningful measurement bias. | |
| **Bias in selection of the reported result** | | 5.1 Were the data that produced this result analysed in accordance with a pre-specified analysis plan that was finalized before unblinded outcome data were available for analysis? | | PY | | The trial was prospectively registered (UMIN000021291) and HbA1c was reported at the fixed 28-week end-of-treatment assessment. Although a standalone dated HbA1c statistical analysis plan was not available, the prospective trial framework, fixed assessment time point, and absence of evidence for result-driven alternative HbA1c analyses were considered sufficient to judge prespecification as probable. | |
|  | | 5.2 Is the numerical result being assessed likely to have been selected, on the basis of the results, from multiple eligible outcome measurements within the outcome domain? | | PN | | HbA1c was reported at the planned 28-week follow-up, and there was no indication that the reported value was selected from multiple eligible HbA1c measurements on the basis of the findings. | |
|  | | 5.3 Is the numerical result being assessed likely to have been selected, on the basis of the results, from multiple eligible analyses of the data? | | PN | | The article described standard treatment-group comparisons, with no indication that the HbA1c result was selected from multiple competing analyses according to statistical significance or effect magnitude. | |
|  | | **Risk of bias judgement** | | **Low** | | HbA1c was assessed at a fixed planned follow-up, and there was no indication that the reported value was selected from multiple eligible measurements or statistical analyses on the basis of the findings. Considering the prospective trial registration and the objective, standard nature of HbA1c, the risk of selective reporting was judged Low. | |
| **Overall bias** | | **Risk of bias judgement** | | **Low** | | Overall risk of bias for the HbA1c result was judged Low. Randomization, deviations from intended interventions, missing outcome data, outcome measurement, and selection of the reported result were all considered unlikely to introduce material bias. | |

## S3.3 Shi 2023

| **Unique ID** | Shi 2023 | | **Study ID** | ChiCTR2100054612 | **Assessor** | | Hanwen Zheng; Jieru Han (consensus) |
| --- | --- | --- | --- | --- | --- | --- | --- |
| **Ref/label** | J Diabetes Complications. 2023;37(10):108610. | | **Aim** | Effect of assignment to intervention |  | |  |
| **Experimental** | Dapagliflozin 10 mg/day + Background | | **Comparator** | Background | **Source** | | https://doi.org/10.1016/j.jdiacomp.2023.108610 |
| **Outcome** | Glycated hemoglobin (HbA1c) | | **Result** | Change from baseline in HbA1c at week 24 (dapagliflozin vs control). | **Weight** | | 1 |
| **Domain** | | **Signalling question / judgement** | | **Response** | | **Comments / justification** | |
| **Bias arising from the randomization process** | | 1.1 Was the allocation sequence random? | | Y | | Eligible participants were assigned 1:1 using computer-generated random numbers (article p. 2). | |
|  | | 1.2 Was the allocation sequence concealed until participants were enrolled and assigned to interventions? | | NI | | Allocation concealment was not described. The report states that a researcher assigned participants according to computer-generated random numbers and the order of registration, but no central allocation or sealed-envelope procedure was reported (article p. 2). | |
|  | | 1.3 Did baseline differences between intervention groups suggest a problem with the randomization process? | | N | | Baseline characteristics were generally comparable between randomized groups, including glycemic measures where reported. No systematic pattern of imbalance suggested a problem with the randomization process. | |
|  | | **Risk of bias judgement** | | **Some concerns** | | The random sequence was generated appropriately, but allocation concealment was not reported. | |
| **Bias due to deviations from intended interventions** | | 2.1 Were participants aware of their assigned intervention during the trial? | | Y | | The trial was explicitly described as open-label, so participants knew their assigned add-on treatment. | |
|  | | 2.2 Were carers and people delivering the interventions aware of participants' assigned intervention during the trial? | | Y | | Because treatment allocation was open-label, clinicians/researchers delivering the assigned glucose-lowering treatment were aware of group assignment. | |
|  | | 2.3 If Y/PY/NI to 2.1 or 2.2: Were there deviations from the intended intervention that arose because of the experimental context? | | PN | | Both groups received the assigned add-on therapy on top of metformin, identical baseline dietary and exercise advice, and the same scheduled follow-up visits. No unplanned differential co-intervention caused by awareness of allocation was reported. | |
|  | | 2.4 If Y/PY to 2.3: Were these deviations likely to have affected the outcome? | | NA | | Not applicable because deviations arising from the experimental context were judged probably absent. | |
|  | | 2.5 If Y/PY/NI to 2.4: Were these deviations from intended intervention balanced between groups? | | NA | | Not applicable because question 2.4 was not required. | |
|  | | 2.6 Was an appropriate analysis used to estimate the effect of assignment to intervention? | | Y | | Participants with available HbA1c data were analysed according to their randomized groups. No treatment switching or reclassification was reported; missing outcome data are considered separately in Domain 3. | |
|  | | 2.7 If N/PN/NI to 2.6: Was there potential for a substantial impact (on the result) of the failure to analyse participants in the group to which they were randomized? | | NA | | Not applicable because question 2.6 was answered Yes. | |
|  | | **Risk of bias judgement** | | **Low** | | Although the study was open-label, no important deviations from intended intervention arising from the trial context were identified, and available HbA1c data were compared according to randomized assignment. | |
| **Bias due to missing outcome data** | | 3.1 Were data for this outcome available for all, or nearly all, participants randomized? | | PN | | Week-24 analyses included 78 of 84 randomized participants (92.9%; 40/42 vs 38/42). The report did not establish that HbA1c was available for every randomized participant. | |
|  | | 3.2 If N/PN/NI to 3.1: Is there evidence that the result was not biased by missing outcome data? | | NI | | Reasons for missing follow-up were reported, but no HbA1c-specific imputation or sensitivity analysis demonstrating robustness to missing data was reported. | |
|  | | 3.3 If N/PN to 3.2: Could missingness in the outcome depend on its true value? | | PY | | Missingness could plausibly be related to subsequent glycemic outcomes because reasons included treatment discontinuation, bariatric surgery, loss to follow-up, and excessive missing values. | |
|  | | 3.4 If Y/PY/NI to 3.3: Is it likely that missingness in the outcome depended on its true value? | | PN | | The number of missing participants was small and reasons were heterogeneous; the report did not show that missingness was likely to depend on the unobserved HbA1c value. | |
|  | | **Risk of bias judgement** | | **Some concerns** | | Some randomized participants lacked follow-up data and no HbA1c-specific missing-data sensitivity analysis was reported; some concerns therefore remain. | |
| **Bias in measurement of the outcome** | | 4.1 Was the method of measuring the outcome inappropriate? | | N | | HbA1c is a standard objective laboratory biomarker of glycemic control; the method of measurement was appropriate for the outcome. | |
|  | | 4.2 Could measurement or ascertainment of the outcome have differed between intervention groups? | | N | | HbA1c was measured using the same clinical laboratory approach and follow-up schedule across randomized groups; no differential ascertainment procedure was reported. | |
|  | | 4.3 Were outcome assessors aware of the intervention received by study participants? | | NI | | Blinding of the laboratory personnel responsible for HbA1c measurement was not specifically reported. | |
|  | | 4.4 If Y/PY/NI to 4.3: Could assessment of the outcome have been influenced by knowledge of intervention received? | | N | | HbA1c is an objective laboratory measurement; knowledge of treatment allocation would not be expected to materially influence the measured value. | |
|  | | 4.5 If Y/PY/NI to 4.4: Is it likely that assessment of the outcome was influenced by knowledge of intervention received? | | NA | | Not applicable because assessment of HbA1c was judged not to be influenced by knowledge of the intervention. | |
|  | | **Risk of bias judgement** | | **Low** | | HbA1c was measured objectively using comparable laboratory procedures across groups; unreported assessor blinding was unlikely to introduce meaningful measurement bias. | |
| **Bias in selection of the reported result** | | 5.1 Were the data that produced this result analysed in accordance with a pre-specified analysis plan that was finalized before unblinded outcome data were available for analysis? | | PY | | The trial was prospectively registered (ChiCTR2100054612) and HbA1c was reported at the fixed 24-week end-of-treatment assessment. Although a standalone dated HbA1c statistical analysis plan was not available, the prospective trial framework, fixed assessment time point, and absence of evidence for result-driven alternative HbA1c analyses were considered sufficient to judge prespecification as probable. | |
|  | | 5.2 Is the numerical result being assessed likely to have been selected, on the basis of the results, from multiple eligible outcome measurements (e.g. scales, definitions, time points) within the outcome domain? | | PN | | The review used the prespecified end-of-treatment assessment at week 24; there was no indication that the reported HbA1c result was chosen from multiple eligible measurements because of the observed findings. | |
|  | | 5.3 Is the numerical result being assessed likely to have been selected, on the basis of the results, from multiple eligible analyses of the data? | | PN | | No evidence indicated that the reported HbA1c result was selected from multiple eligible analyses on the basis of statistical significance or effect size. | |
|  | | **Risk of bias judgement** | | **Low** | | HbA1c was assessed at a fixed planned follow-up, and there was no indication that the reported value was selected from multiple eligible measurements or statistical analyses on the basis of the findings. Considering the prospective trial registration and the objective, standard nature of HbA1c, the risk of selective reporting was judged Low. | |
| **Overall bias** | | **Risk of bias judgement** | | **Some concerns** | | Overall risk of bias for the HbA1c result was judged Some concerns. The remaining uncertainties related mainly to allocation concealment and incomplete follow-up data; the other domains were judged Low. | |

## S3.4 Kuchay 2020

| **Unique ID** | Kuchay 2020 | | **Study ID** | NCT03590626 | **Assessor** | | Hanwen Zheng; Jieru Han (consensus) |
| --- | --- | --- | --- | --- | --- | --- | --- |
| **Ref/label** | Diabetologia. 2020;63(11):2434–2445. | | **Aim** | Effect of assignment to intervention |  | |  |
| **Experimental** | Dulaglutide 0.75 mg weekly for 4 weeks, then 1.5 mg weekly for 20 weeks + standard diabetes care | | **Comparator** | Standard diabetes care without GLP-1 receptor agonist | **Source** | | https://doi.org/10.1007/s00125-020-05265-7 |
| **Outcome** | Glycated hemoglobin (HbA1c) | | **Result** | Change from baseline in HbA1c at week 24 (dulaglutide vs standard diabetes care). | **Weight** | | 1 |
| **Domain** | | **Signalling question / judgement** | | **Response** | | **Comments / justification** | |
| **Bias arising from the randomization process** | | 1.1 Was the allocation sequence random? | | Y | | A predefined computer-generated random-number sequence was used for 1:1 allocation. | |
|  | | 1.2 Was the allocation sequence concealed until participants were enrolled and assigned to interventions? | | Y | | Allocation was concealed using serially numbered, opaque, sealed envelopes prepared before assignment. | |
|  | | 1.3 Did baseline differences between intervention groups suggest a problem with the randomization process? | | N | | Baseline characteristics were generally comparable between randomized groups, including glycemic measures where reported. No systematic pattern of imbalance suggested a problem with the randomization process. | |
|  | | **Risk of bias judgement** | | **Low** | | The random sequence and allocation concealment were adequately described, and no important baseline imbalance relevant to HbA1c was identified. | |
| **Bias due to deviations from intended interventions** | | 2.1 Were participants aware of their assigned intervention during the trial? | | Y | | The trial was open-label, so participants knew whether dulaglutide had been added to usual care. | |
|  | | 2.2 Were carers and people delivering the interventions aware of participants' assigned intervention during the trial? | | Y | | Treating clinicians were aware of allocation because treatment was open-label and background glucose-lowering therapy was adjusted clinically. | |
|  | | 2.3 If Y/PY/NI to 2.1 or 2.2: Were there deviations from the intended intervention that arose because of the experimental context? | | PN | | No crossover or unplanned differential co-intervention was reported. Lifestyle advice and diabetes management were standardized; stopping DPP-4 inhibitors in the dulaglutide group was protocol-specified. | |
|  | | 2.4 If Y/PY to 2.3: Were these deviations likely to have affected the outcome? | | NA | | Not applicable because important deviations arising from the experimental context were judged probably absent. | |
|  | | 2.5 If Y/PY/NI to 2.4: Were these deviations from intended intervention balanced between groups? | | NA | | Not applicable because question 2.4 was not required. | |
|  | | 2.6 Was an appropriate analysis used to estimate the effect of assignment to intervention? | | Y | | The HbA1c comparison retained participants in their randomized treatment groups. Exclusion of participants without week-24 data is considered in Domain 3 rather than as reclassification by treatment received. | |
|  | | 2.7 If N/PN/NI to 2.6: Was there potential for a substantial impact (on the result) of the failure to analyse participants in the group to which they were randomized? | | NA | | Not applicable because the randomized-group comparison was preserved. | |
|  | | **Risk of bias judgement** | | **Low** | | Open-label status did not itself create a bias: no important trial-context deviations were identified, co-interventions were protocol-guided, and analyzed participants remained in their randomized groups. | |
| **Bias due to missing outcome data** | | 3.1 Were data for this outcome available for all, or nearly all, participants randomized? | | N | | Week-24 HbA1c data in the reported per-protocol table were available for 52 of 64 randomized participants: 27/32 in the dulaglutide group and 25/32 in the control group. | |
|  | | 3.2 If N/PN/NI to 3.1: Is there evidence that the result was not biased by missing outcome data? | | PY | | Attrition was similar between groups (5 vs 7). Reported reasons included gastrointestinal adverse effects, loss to follow-up, and protocol violations; there was no clear indication that missingness was driven by the unobserved HbA1c value, so material bias in the HbA1c contrast was judged unlikely. | |
|  | | 3.3 If N/PN to 3.2: Could missingness in the outcome depend on its true value? | | NA | | Not applicable because question 3.2 was answered Probably yes. | |
|  | | 3.4 If Y/PY/NI to 3.3: Is it likely that missingness in the outcome depended on its true value? | | NA | | Not applicable because question 3.3 was not required. | |
|  | | **Risk of bias judgement** | | **Low** | | Although HbA1c was reported for 52/64 randomized participants, attrition was reasonably balanced and documented, with no clear evidence that missingness depended on the unobserved HbA1c value. | |
| **Bias in measurement of the outcome** | | 4.1 Was the method of measuring the outcome inappropriate? | | N | | HbA1c is a standard objective laboratory biomarker of glycemic control; the method of measurement was appropriate for the outcome. | |
|  | | 4.2 Could measurement or ascertainment of the outcome have differed between intervention groups? | | N | | HbA1c was measured using the same clinical laboratory approach and follow-up schedule across randomized groups; no differential ascertainment procedure was reported. | |
|  | | 4.3 Were outcome assessors aware of the intervention received by study participants? | | NI | | Blinding of the laboratory personnel responsible for HbA1c measurement was not specifically reported. | |
|  | | 4.4 If Y/PY/NI to 4.3: Could assessment of the outcome have been influenced by knowledge of intervention received? | | N | | HbA1c is an objective laboratory measurement; knowledge of treatment allocation would not be expected to materially influence the measured value. | |
|  | | 4.5 If Y/PY/NI to 4.4: Is it likely that assessment of the outcome was influenced by knowledge of intervention received? | | NA | | Not applicable because assessment of HbA1c was judged not to be influenced by knowledge of the intervention. | |
|  | | **Risk of bias judgement** | | **Low** | | HbA1c was measured objectively using comparable laboratory procedures across groups; unreported assessor blinding was unlikely to introduce meaningful measurement bias. | |
| **Bias in selection of the reported result** | | 5.1 Were the data that produced this result analysed in accordance with a pre-specified analysis plan that was finalized before unblinded outcome data were available for analysis? | | PY | | The trial was prospectively registered (NCT03590626) and HbA1c was reported at the fixed 24-week end-of-treatment assessment. Although a standalone dated HbA1c statistical analysis plan was not available, the prospective trial framework, fixed assessment time point, and absence of evidence for result-driven alternative HbA1c analyses were considered sufficient to judge prespecification as probable. | |
|  | | 5.2 ... multiple eligible outcome measurements (e.g. scales, definitions, time points) within the outcome domain? | | PN | | HbA1c was reported at the fixed 24-week follow-up used for the randomized comparison, with no indication that the reported measurement was selected from multiple eligible HbA1c time points on the basis of the results. | |
|  | | 5.3 ... multiple eligible analyses of the data? | | PN | | No evidence indicated selection of the reported HbA1c result from multiple competing statistical analyses. | |
|  | | **Risk of bias judgement** | | **Low** | | HbA1c was assessed at a fixed planned follow-up, and there was no indication that the reported value was selected from multiple eligible measurements or statistical analyses on the basis of the findings. Considering the prospective trial registration and the objective, standard nature of HbA1c, the risk of selective reporting was judged Low. | |
| **Overall bias** | | **Risk of bias judgement** | | **Low** | | Overall risk of bias for the HbA1c result was judged Low. No domain was considered likely to introduce material bias into the outcome-specific estimate. | |

## S3.5 Attaran 2023

| **Unique ID** | Attaran 2023 | | **Study ID** | IRCT20190122042450N5 | **Assessor** | | Hanwen Zheng; Jieru Han (consensus) |
| --- | --- | --- | --- | --- | --- | --- | --- |
| **Ref/label** | BMC Gastroenterol. 2023;23(1):327. | | **Aim** | Effect of assignment to intervention |  | |  |
| **Experimental** | Empagliflozin 10 mg/day for 24 weeks + background diabetes care | | **Comparator** | Pioglitazone 30 mg/day for 24 weeks + background diabetes care | **Source** | | Journal article; Iranian Registry of Clinical Trials IRCT20190122042450N5 |
| **Outcome** | Glycated hemoglobin (HbA1c) | | **Result** | Change from baseline in HbA1c at week 24 (empagliflozin vs pioglitazone). | **Weight** | | 1 |
| **Domain** | | **Signalling question / judgement** | | **Response** | | **Comments / justification** | |
| **Bias arising from the randomization process** | | 1.1 Was the allocation sequence random? | | Y | | A computerized block-randomization method was used to allocate participants 1:1. | |
|  | | 1.2 Was the allocation sequence concealed until participants were enrolled and assigned to interventions? | | Y | | Study medications were sealed, sequentially numbered according to the allocation sequence, and an independent staff member generated the sequence and assigned participants. | |
|  | | 1.3 Did baseline differences between intervention groups suggest a problem with the randomization process? | | N | | Baseline characteristics were generally comparable between randomized groups, including glycemic measures where reported. No systematic pattern of imbalance suggested a problem with the randomization process. | |
|  | | **Risk of bias judgement** | | **Low** | | Computerized block randomization, sequentially numbered sealed allocation, independent assignment and broadly balanced baseline characteristics supported Low risk. | |
| **Bias due to deviations from intended interventions** | | 2.1 Were participants aware of their assigned intervention during the trial? | | NI | | The study was described as single-blind, but the report does not clearly state that participants themselves were blinded. | |
|  | | 2.2 Were carers and people delivering the interventions aware of participants' assigned intervention during the trial? | | N | | The article explicitly states that the care provider was blinded to the intervention. | |
|  | | 2.3 If Y/PY/NI to 2.1 or 2.2: Were there deviations from the intended intervention that arose because of the experimental context? | | N | | No important trial-context deviation was identified. Both groups received standard lifestyle advice; background anti-hyperglycemic therapy could be adjusted according to the same ADA guidance, while study-drug doses remained constant. | |
|  | | 2.4 If Y/PY to 2.3: Were these deviations likely to have affected the outcome? | | NA | | Not applicable because no important trial-context deviation was identified. | |
|  | | 2.5 If Y/PY/NI to 2.4: Were these deviations from intended intervention balanced between groups? | | NA | | Not applicable because question 2.4 was not required. | |
|  | | 2.6 Was an appropriate analysis used to estimate the effect of assignment to intervention? | | Y | | The authors explicitly state that data were analyzed by the intention-to-treat method and all 73 randomized participants were included according to assigned group. | |
|  | | 2.7 If N/PN/NI to 2.6: Was there potential for a substantial impact (on the result) of the failure to analyse participants in the group to which they were randomized? | | NA | | Not applicable because an intention-to-treat analysis was used. | |
|  | | **Risk of bias judgement** | | **Low** | | Care-provider blinding, absence of important differential deviations, and explicit intention-to-treat analysis supported Low risk for deviations from intended intervention. | |
| **Bias due to missing outcome data** | | 3.1 Were data for this outcome available for all, or nearly all, participants randomized? | | PY | | Seventy-three participants were randomized; three did not complete treatment because of non-adherence, but the article states that data from all 73 randomized participants were used in the intention-to-treat analysis. | |
|  | | 3.2 If N/PN/NI to 3.1: Is there evidence that the result was not biased by missing outcome data? | | NA | | Not applicable because outcome data were judged available for all or nearly all participants under the reported intention-to-treat analysis. | |
|  | | 3.3 If N/PN to 3.2: Could missingness in the outcome depend on its true value? | | NA | | Not applicable. | |
|  | | 3.4 If Y/PY/NI to 3.3: Is it likely that missingness in the outcome depended on its true value? | | NA | | Not applicable. | |
|  | | **Risk of bias judgement** | | **Low** | | Only three participants discontinued treatment, and all randomized participants were retained in the reported intention-to-treat analysis. | |
| **Bias in measurement of the outcome** | | 4.1 Was the method of measuring the outcome inappropriate? | | N | | HbA1c is a standard objective laboratory biomarker of glycemic control; the method of measurement was appropriate for the outcome. | |
|  | | 4.2 Could measurement or ascertainment of the outcome have differed between intervention groups? | | N | | HbA1c was measured using the same clinical laboratory approach and follow-up schedule across randomized groups; no differential ascertainment procedure was reported. | |
|  | | 4.3 Were outcome assessors aware of the intervention received by study participants? | | NI | | Blinding of the laboratory personnel responsible for HbA1c measurement was not specifically reported. | |
|  | | 4.4 If Y/PY/NI to 4.3: Could assessment of the outcome have been influenced by knowledge of intervention received? | | N | | HbA1c is an objective laboratory measurement; knowledge of treatment allocation would not be expected to materially influence the measured value. | |
|  | | 4.5 If Y/PY/NI to 4.4: Is it likely that assessment of the outcome was influenced by knowledge of intervention received? | | NA | | Not applicable because assessment of HbA1c was judged not to be influenced by knowledge of the intervention. | |
|  | | **Risk of bias judgement** | | **Low** | | HbA1c was measured objectively using comparable laboratory procedures across groups; unreported assessor blinding was unlikely to introduce meaningful measurement bias. | |
| **Bias in selection of the reported result** | | 5.1 Were the data that produced this result analysed in accordance with a pre-specified analysis plan that was finalized before unblinded outcome data were available for analysis? | | PY | | The trial was prospectively registered (IRCT20190122042450N5) and HbA1c was reported at the fixed 24-week end-of-treatment assessment. Although a standalone dated HbA1c statistical analysis plan was not available, the prospective trial framework, fixed assessment time point, and absence of evidence for result-driven alternative HbA1c analyses were considered sufficient to judge prespecification as probable. | |
|  | | 5.2 ... multiple eligible outcome measurements (e.g. scales, definitions, time points) within the outcome domain? | | PN | | HbA1c was assessed at the fixed 24-week follow-up used for the treatment comparison; selection from multiple eligible outcome measurements on the basis of the results was not evident. | |
|  | | 5.3 ... multiple eligible analyses of the data? | | PN | | The report does not suggest that the HbA1c result was selected from multiple competing analyses according to statistical significance or effect magnitude. | |
|  | | **Risk of bias judgement** | | **Low** | | HbA1c was assessed at a fixed planned follow-up, and there was no indication that the reported value was selected from multiple eligible measurements or statistical analyses on the basis of the findings. Considering the prospective trial registration and the objective, standard nature of HbA1c, the risk of selective reporting was judged Low. | |
| **Overall bias** | | **Risk of bias judgement** | | **Low** | | Overall risk of bias for the HbA1c result was judged Low. The randomized design, controlled intervention delivery, minimal missing data, objective HbA1c measurement, and lack of evidence for selective reporting supported this judgment. | |

## S3.6 Kuchay 2018

| **Unique ID** | Kuchay 2018 | | **Study ID** | NCT02686476 | **Assessor** | | Hanwen Zheng; Jieru Han (consensus) |
| --- | --- | --- | --- | --- | --- | --- | --- |
| **Ref/label** | Diabetes Care. 2018;41(8):1801–1808. | | **Aim** | Effect of assignment to intervention |  | |  |
| **Experimental** | Empagliflozin | | **Comparator** | Standard diabetes care | **Source** | | https://doi.org/10.2337/dc18-0165 |
| **Outcome** | Glycated hemoglobin (HbA1c) | | **Result** | Change from baseline in HbA1c at week 20 (empagliflozin vs standard diabetes care). | **Weight** | | 1 |
| **Domain** | | **Signalling question / judgement** | | **Response** | | **Comments / justification** | |
| **Bias arising from the randomization process** | | 1.1 Was the allocation sequence random? | | Y | | A research assistant randomized participants 1:1 using computer-generated numbers. | |
|  | | 1.2 Was the allocation sequence concealed until participants were enrolled and assigned to interventions? | | NI | | The paper has a section titled randomization and allocation concealment, but it does not describe a specific concealment mechanism such as central randomization or sealed opaque envelopes. | |
|  | | 1.3 Did baseline differences between intervention groups suggest a problem with the randomization process? | | N | | Baseline characteristics were generally comparable between randomized groups, including glycemic measures where reported. No systematic pattern of imbalance suggested a problem with the randomization process. | |
|  | | **Risk of bias judgement** | | **Some concerns** | | The sequence was random, but allocation concealment before assignment was not described clearly. | |
| **Bias due to deviations from intended interventions** | | 2.1 Were participants aware of their assigned intervention during the trial? | | Y | | The E-LIFT trial was explicitly open-label, so participants knew whether empagliflozin was added. | |
|  | | 2.2 Were carers and people delivering the interventions aware of participants' assigned intervention during the trial? | | Y | | Treating endocrinologists adjusted diabetes treatment according to randomized assignment and therefore were aware of allocation. | |
|  | | 2.3 If Y/PY/NI to 2.1 or 2.2: Were there deviations from the intended intervention that arose because of the experimental context? | | PN | | Background glucose-lowering treatment could be adjusted to maintain glycaemic equipoise, but this was part of the planned treatment strategy. No treatment crossover or unplanned differential co-intervention was reported. | |
|  | | 2.4 If Y/PY to 2.3: Were these deviations likely to have affected the outcome? | | NA | | Not applicable because deviations arising from the experimental context were judged probably absent. | |
|  | | 2.5 If Y/PY/NI to 2.4: Were these deviations from intended intervention balanced between groups? | | NA | | Not applicable. | |
|  | | 2.6 Was an appropriate analysis used to estimate the effect of assignment to intervention? | | PY | | Participants with available HbA1c data were analysed according to their randomized groups. Exclusion of participants without week-20 measurements is addressed under missing outcome data rather than as a per-protocol reclassification. | |
|  | | 2.7 If N/PN/NI to 2.6: Was there potential for a substantial impact (on the result) of the failure to analyse participants in the group to which they were randomized? | | NA | | Not applicable because question 2.6 was answered probably yes. | |
|  | | **Risk of bias judgement** | | **Low** | | Although the trial was open-label, no important trial-context deviations from the intended interventions were identified, and analysed participants remained in their randomized groups. | |
| **Bias due to missing outcome data** | | 3.1 Were data for this outcome available for all, or nearly all, participants randomized? | | N | | Week-20 outcome data were available for 42 of 50 randomized participants (22/25 empagliflozin and 20/25 control), so HbA1c data were not available for all or nearly all participants. | |
|  | | 3.2 If N/PN/NI to 3.1: Is there evidence that the result was not biased by missing outcome data? | | PY | | Reasons for missing follow-up were reported and were not suggestive of the unobserved HbA1c value: drug-related complications in the empagliflozin arm and loss to follow-up or work-schedule conflict in the control arm. Missingness was also reasonably balanced. | |
|  | | 3.3 If N/PN to 3.2: Could missingness in the outcome depend on its true value? | | NA | | Not applicable because question 3.2 was answered probably yes. | |
|  | | 3.4 If Y/PY/NI to 3.3: Is it likely that missingness in the outcome depended on its true value? | | NA | | Not applicable. | |
|  | | **Risk of bias judgement** | | **Low** | | Despite moderate attrition, the documented reasons for missing follow-up were unlikely to depend on the true HbA1c value, so important bias from missing HbA1c data was considered unlikely. | |
| **Bias in measurement of the outcome** | | 4.1 Was the method of measuring the outcome inappropriate? | | N | | HbA1c is a standard objective laboratory biomarker of glycemic control; the method of measurement was appropriate for the outcome. | |
|  | | 4.2 Could measurement or ascertainment of the outcome have differed between intervention groups? | | N | | HbA1c was measured using the same clinical laboratory approach and follow-up schedule across randomized groups; no differential ascertainment procedure was reported. | |
|  | | 4.3 Were outcome assessors aware of the intervention received by study participants? | | NI | | Blinding of the laboratory personnel responsible for HbA1c measurement was not specifically reported. | |
|  | | 4.4 If Y/PY/NI to 4.3: Could assessment of the outcome have been influenced by knowledge of intervention received? | | N | | HbA1c is an objective laboratory measurement; knowledge of treatment allocation would not be expected to materially influence the measured value. | |
|  | | 4.5 If Y/PY/NI to 4.4: Is it likely that assessment of the outcome was influenced by knowledge of intervention received? | | NA | | Not applicable because assessment of HbA1c was judged not to be influenced by knowledge of the intervention. | |
|  | | **Risk of bias judgement** | | **Low** | | HbA1c was measured objectively using comparable laboratory procedures across groups; unreported assessor blinding was unlikely to introduce meaningful measurement bias. | |
| **Bias in selection of the reported result** | | 5.1 Were the data that produced this result analysed in accordance with a pre-specified analysis plan that was finalized before unblinded outcome data were available for analysis? | | NI | | Although the trial was prospectively registered (NCT02686476), prospective prespecification of HbA1c as a registry outcome could not be verified. HbA1c was reported at the 20-week end-of-treatment assessment, but no dated statistical analysis plan was available to confirm that the HbA1c analysis was finalized before unblinded outcome data were available. | |
|  | | 5.2 ... multiple eligible outcome measurements (e.g. scales, definitions, time points) within the outcome domain? | | PN | | HbA1c was reported at the fixed end-of-treatment assessment, and there was no indication that the numerical result was selected from multiple eligible HbA1c measurements because of the findings. | |
|  | | 5.3 ... multiple eligible analyses of the data? | | PN | | No evidence indicated selective choice among multiple eligible analyses of HbA1c. | |
|  | | **Risk of bias judgement** | | **Some concerns** | | HbA1c was reported at the fixed 20-week end-of-treatment assessment, but prospective prespecification of the HbA1c analysis could not be fully verified; the risk of selective reporting was therefore judged Some concerns. | |
| **Overall bias** | | **Risk of bias judgement** | | **Some concerns** | | Overall risk of bias for the HbA1c result was judged Some concerns because allocation concealment was not described clearly (Domain 1) and prospective prespecification of the HbA1c analysis could not be fully verified (Domain 5). The remaining domains were judged Low. | |

## S3.7 Guo 2020

| **Unique ID** | Guo 2020 | | **Study ID** | ChiCTR2000035091 | **Assessor** | | Hanwen Zheng; Jieru Han (consensus) |
| --- | --- | --- | --- | --- | --- | --- | --- |
| **Ref/label** | Diabetes Res Clin Pract. 2020;170:108487. | | **Aim** | Effect of assignment to intervention |  | |  |
| **Experimental** | Liraglutide | | **Comparator** | Placebo | **Source** | | https://doi.org/10.1016/j.diabres.2020.108487 |
| **Outcome** | Glycated hemoglobin (HbA1c) | | **Result** | Change from baseline in HbA1c at week 26 (liraglutide vs placebo). | **Weight** | | 1 |
| **Domain** | | **Signalling question / judgement** | | **Response** | | **Comments / justification** | |
| **Bias arising from the randomization process** | | 1.1 Was the allocation sequence random? | | Y | | Patients were randomized by the trial pharmacist using computer-generated numbers with a 1:1:1 allocation ratio and block size of six. | |
|  | | 1.2 Was the allocation sequence concealed until participants were enrolled and assigned to interventions? | | PY | | Allocation was implemented by the trial pharmacist rather than the treating clinicians. Although the concealment procedure was not described in full detail, assignment was probably concealed until allocation. | |
|  | | 1.3 Did baseline differences between intervention groups suggest a problem with the randomization process? | | N | | Baseline characteristics were generally comparable between randomized groups, including glycemic measures where reported. No systematic pattern of imbalance suggested a problem with the randomization process. | |
|  | | **Risk of bias judgement** | | **Low** | | Computer-generated blocked randomization was implemented by a trial pharmacist, and there was no evidence of a baseline imbalance indicating failure of randomization. | |
| **Bias due to deviations from intended interventions** | | 2.1 Were participants aware of their assigned intervention during the trial? | | Y | | The trial was explicitly described as not blinded, so participants were aware of the assigned treatment. | |
|  | | 2.2 Were carers and people delivering the interventions aware of participants' assigned intervention during the trial? | | Y | | The active treatments required drug-specific administration and dose adjustment, so personnel delivering treatment were aware of allocation. | |
|  | | 2.3 If Y/PY/NI to 2.1 or 2.2: Were there deviations from the intended intervention that arose because of the experimental context? | | PN | | Metformin was maintained at a constant dose and all groups received the same lifestyle advice. Dose titration of study drugs was protocol-driven, and no unplanned differential co-intervention or crossover was reported. | |
|  | | 2.4 If Y/PY to 2.3: Were these deviations likely to have affected the outcome? | | NA | | Not applicable because question 2.3 was answered probably no. | |
|  | | 2.5 If Y/PY/NI to 2.4: Were these deviations from intended intervention balanced between groups? | | NA | | Not applicable. | |
|  | | 2.6 Was an appropriate analysis used to estimate the effect of assignment to intervention? | | PY | | Participants with available week-26 HbA1c data were analysed in their originally assigned groups; there was no indication of treatment switching or reclassification by treatment received. | |
|  | | 2.7 If N/PN/NI to 2.6: Was there potential for a substantial impact (on the result) of the failure to analyse participants in the group to which they were randomized? | | NA | | Not applicable because question 2.6 was answered probably yes. | |
|  | | **Risk of bias judgement** | | **Low** | | Open-label treatment did not lead to identified important deviations from the intended interventions, and the HbA1c comparison retained randomized group assignment. | |
| **Bias due to missing outcome data** | | 3.1 Were data for this outcome available for all, or nearly all, participants randomized? | | PY | | Ninety-one of 96 randomized participants completed the 26-week trial (30 insulin glargine, 31 liraglutide, 30 placebo), so HbA1c data were available for nearly all randomized participants. | |
|  | | 3.2 If N/PN/NI to 3.1: Is there evidence that result was not biased by missing outcome data? | | NA | | Not applicable because question 3.1 was answered probably yes. | |
|  | | 3.3 If N/PN to 3.2: Could missingness in the outcome depend on its true value? | | NA | | Not applicable. | |
|  | | 3.4 If Y/PY/NI to 3.3: Is it likely that missingness in the outcome depended on its true value? | | NA | | Not applicable. | |
|  | | **Risk of bias judgement** | | **Low** | | Only five of 96 randomized participants did not complete follow-up, so the small amount of missing HbA1c data was unlikely to materially bias the result. | |
| **Bias in measurement of the outcome** | | 4.1 Was the method of measuring the outcome inappropriate? | | N | | HbA1c is a standard objective laboratory biomarker of glycemic control; the method of measurement was appropriate for the outcome. | |
|  | | 4.2 Could measurement or ascertainment of the outcome have differed between intervention groups? | | N | | HbA1c was measured using the same clinical laboratory approach and follow-up schedule across randomized groups; no differential ascertainment procedure was reported. | |
|  | | 4.3 Were outcome assessors aware of the intervention received by study participants? | | NI | | Blinding of the laboratory personnel responsible for HbA1c measurement was not specifically reported. | |
|  | | 4.4 If Y/PY/NI to 4.3: Could assessment of the outcome have been influenced by knowledge of intervention received? | | N | | HbA1c is an objective laboratory measurement; knowledge of treatment allocation would not be expected to materially influence the measured value. | |
|  | | 4.5 If Y/PY/NI to 4.4: Is it likely that assessment of the outcome was influenced by knowledge of intervention received? | | NA | | Not applicable because assessment of HbA1c was judged not to be influenced by knowledge of the intervention. | |
|  | | **Risk of bias judgement** | | **Low** | | HbA1c was measured objectively using comparable laboratory procedures across groups; unreported assessor blinding was unlikely to introduce meaningful measurement bias. | |
| **Bias in selection of the reported result** | | 5.1 Were the data that produced this result analysed in accordance with a pre-specified analysis plan that was finalized before unblinded outcome data were available for analysis? | | NI | | HbA1c and the 26-week assessment were reported in the article, but the available report and registry information do not establish that a detailed HbA1c analysis plan was finalized before unblinded outcome data were available. | |
|  | | 5.2 ... multiple eligible outcome measurements (e.g. scales, definitions, time points) within the outcome domain? | | PN | | The randomized comparison used the fixed 26-week follow-up, and there was no evidence that a particular HbA1c measurement was selected because it produced a more favorable result. | |
|  | | 5.3 ... multiple eligible analyses of the data? | | PN | | No indication was found that the HbA1c result was selected from multiple competing statistical analyses on the basis of the observed findings. | |
|  | | **Risk of bias judgement** | | **Some concerns** | | HbA1c was reported at the fixed 26-week follow-up, but prospective finalization of the HbA1c analysis plan before access to unblinded outcome data could not be confirmed; the risk of selective reporting was therefore judged Some concerns. | |
| **Overall bias** | | **Risk of bias judgement** | | **Some concerns** | | Overall risk of bias for the HbA1c result was judged Some concerns because prospective prespecification of the HbA1c analysis could not be confirmed (Domain 5); the other domains were judged Low. | |

## S3.8 Horibe 2022

| **Unique ID** | Horibe 2022 | | **Study ID** | UMIN000020239 / jRCTs051180018 | **Assessor** | | Hanwen Zheng; Jieru Han (consensus) |
| --- | --- | --- | --- | --- | --- | --- | --- |
| **Ref/label** | Diabetes Res Clin Pract. 2022;186:109781. | | **Aim** | Effect of assignment to intervention |  | |  |
| **Experimental** | Dapagliflozin + standard treatment | | **Comparator** | Standard treatment; non-SGLT2 antidiabetic therapy could be adjusted according to protocol | **Source** | | https://doi.org/10.1016/j.diabres.2022.109781 |
| **Outcome** | Glycated hemoglobin (HbA1c) | | **Result** | Change from baseline in HbA1c at week 24 (dapagliflozin vs standard treatment). | **Weight** | | 1 |
| **Domain** | | **Signalling question / judgement** | | **Response** | | **Comments / justification** | |
| **Bias arising from the randomization process** | | 1.1 Was the allocation sequence random? | | Y | | Eligible participants were randomized using a web-based, password-protected system based on a computer-generated random sequence, with sex as a stratification factor. | |
|  | | 1.2 Was the allocation sequence concealed until participants were enrolled and assigned to interventions? | | Y | | Randomization was performed by the clinical research development office, independent of the investigators, after eligibility was confirmed and the patient was registered. | |
|  | | 1.3 Did baseline differences between intervention groups suggest a problem with the randomization process? | | N | | Baseline characteristics were generally comparable between randomized groups, including glycemic measures where reported. No systematic pattern of imbalance suggested a problem with the randomization process. | |
|  | | **Risk of bias judgement** | | **Low** | | The sequence generation and centralized independent allocation process were appropriate and adequately concealed. | |
| **Bias due to deviations from intended interventions** | | 2.1 Were participants aware of their assigned intervention during the trial? | | Y | | The trial was open-label, so participants were aware of dapagliflozin assignment. | |
|  | | 2.2 Were carers and people delivering the interventions aware of participants' assigned intervention during the trial? | | Y | | Treating investigators implemented dapagliflozin and protocol-specified background-treatment adjustments and were aware of allocation. | |
|  | | 2.3 If Y/PY/NI to 2.1 or 2.2: Were there deviations from the intended intervention that arose because of the experimental context? | | PN | | Sulfonylurea dose reduction in the dapagliflozin group and possible addition of non-SGLT2 agents in controls were prespecified components of the treatment strategy. No important unplanned deviations were reported. | |
|  | | 2.4 If Y/PY to 2.3: Were these deviations likely to have affected the outcome? | | NA | | Not applicable because question 2.3 was answered probably no. | |
|  | | 2.5 If Y/PY/NI to 2.4: Were these deviations from intended intervention balanced between groups? | | NA | | Not applicable. | |
|  | | 2.6 Was an appropriate analysis used to estimate the effect of assignment to intervention? | | Y | | The authors explicitly stated that statistical analyses were performed on an intention-to-treat basis, and the HbA1c analysis retained participants in their randomized groups. | |
|  | | 2.7 If N/PN/NI to 2.6: Was there potential for a substantial impact (on the result) of the failure to analyse participants in the group to which they were randomized? | | NA | | Not applicable because question 2.6 was answered yes. | |
|  | | **Risk of bias judgement** | | **Low** | | The open-label design did not lead to identified important deviations, and an intention-to-treat analysis was used for the HbA1c result. | |
| **Bias due to missing outcome data** | | 3.1 Were data for this outcome available for all, or nearly all, participants randomized? | | Y | | HbA1c was included in the intention-to-treat analysis of 50 participants (26 dapagliflozin and 24 control), representing more than 95% of the 52 randomized participants. | |
|  | | 3.2 If N/PN/NI to 3.1: Is there evidence that result was not biased by missing outcome data? | | NA | | Not applicable because question 3.1 was answered yes. | |
|  | | 3.3 If N/PN to 3.2: Could missingness in the outcome depend on its true value? | | NA | | Not applicable. | |
|  | | 3.4 If Y/PY/NI to 3.3: Is it likely that missingness in the outcome depended on its true value? | | NA | | Not applicable. | |
|  | | **Risk of bias judgement** | | **Low** | | HbA1c data were available for nearly all randomized participants; the two participants without follow-up withdrew before commencing the study protocol. | |
| **Bias in measurement of the outcome** | | 4.1 Was the method of measuring the outcome inappropriate? | | N | | HbA1c is a standard objective laboratory biomarker of glycemic control; the method of measurement was appropriate for the outcome. | |
|  | | 4.2 Could measurement or ascertainment of the outcome have differed between intervention groups? | | N | | HbA1c was measured using the same clinical laboratory approach and follow-up schedule across randomized groups; no differential ascertainment procedure was reported. | |
|  | | 4.3 Were outcome assessors aware of the intervention received by study participants? | | NI | | Blinding of the laboratory personnel responsible for HbA1c measurement was not specifically reported. | |
|  | | 4.4 If Y/PY/NI to 4.3: Could assessment of the outcome have been influenced by knowledge of intervention received? | | N | | HbA1c is an objective laboratory measurement; knowledge of treatment allocation would not be expected to materially influence the measured value. | |
|  | | 4.5 If Y/PY/NI to 4.4: Is it likely that assessment of the outcome was influenced by knowledge of intervention received? | | NA | | Not applicable because assessment of HbA1c was judged not to be influenced by knowledge of the intervention. | |
|  | | **Risk of bias judgement** | | **Low** | | HbA1c was measured objectively using comparable laboratory procedures across groups; unreported assessor blinding was unlikely to introduce meaningful measurement bias. | |
| **Bias in selection of the reported result** | | 5.1 Were the data that produced this result analysed in accordance with a pre-specified analysis plan that was finalized before unblinded outcome data were available for analysis? | | PY | | Prospective registry information (UMIN000020239/jRCTs051180018) explicitly prespecified change in HbA1c as a secondary glucose-metabolism outcome assessed from baseline to week 24. | |
|  | | 5.2 ... multiple eligible outcome measurements (e.g. scales, definitions, time points) within the outcome domain? | | PN | | The reported HbA1c result corresponded to the prespecified 24-week assessment, with no indication that it was selected from multiple eligible measurements on the basis of the findings. | |
|  | | 5.3 ... multiple eligible analyses of the data? | | PN | | The reported analysis followed the planned randomized-group comparison, with no indication of selective choice among multiple eligible analyses. | |
|  | | **Risk of bias judgement** | | **Low** | | HbA1c and its 24-week assessment were prospectively specified, and no evidence suggested selective reporting of the numerical result. | |
| **Overall bias** | | **Risk of bias judgement** | | **Low** | | Overall risk of bias for the HbA1c result was judged Low. All five RoB 2 domains were judged Low for this outcome-specific result. | |

## S3.9 Zhang 2020

| **Unique ID** | Zhang 2020 | | **Study ID** | Not reported | **Assessor** | | Hanwen Zheng; Jieru Han (consensus) |
| --- | --- | --- | --- | --- | --- | --- | --- |
| **Ref/label** | Clin Res Hepatol Gastroenterol. 2020;44(5):674–680. | | **Aim** | Effect of assignment to intervention |  | |  |
| **Experimental** | Liraglutide 0.6 mg/day (week 1), titrated to 1.2 mg/day (weeks 2–24) + metformin + diet/exercise advice | | **Comparator** | Pioglitazone 15 mg/day (week 1), titrated to 30 mg/day (weeks 2–24) + metformin + diet/exercise advice | **Source** | | https://doi.org/10.1016/j.clinre.2020.01.007 |
| **Outcome** | Glycated hemoglobin (HbA1c) | | **Result** | Change from baseline in HbA1c at week 24 (liraglutide vs pioglitazone). | **Weight** | | 1 |
| **Domain** | | **Signalling question / judgement** | | **Response** | | **Comments / justification** | |
| **Bias arising from the randomization process** | | 1.1 Was the allocation sequence random? | | Y | | Participants were randomized 1:1 using a computer-generated sequence. | |
|  | | 1.2 Was the allocation sequence concealed until participants were enrolled and assigned to interventions? | | NI | | The report did not describe central randomization, sealed envelopes, pharmacy-controlled concealment, or another method ensuring that the allocation sequence was concealed before assignment. | |
|  | | 1.3 Did baseline differences between intervention groups suggest a problem with the randomization process? | | N | | Baseline characteristics were generally comparable between randomized groups, including glycemic measures where reported. No systematic pattern of imbalance suggested a problem with the randomization process. | |
|  | | **Risk of bias judgement** | | **Some concerns** | | The allocation sequence was random, but the method used to conceal assignment before enrolment was not reported. | |
| **Bias due to deviations from intended interventions** | | 2.1 Were participants aware of their assigned intervention during the trial? | | Y | | The abstract and Methods describe the study as open-label. The same Methods sentence also uses the term double-blind, but no double-dummy or masking procedure is described; participants were therefore considered aware of treatment assignment. | |
|  | | 2.2 Were carers and people delivering the interventions aware of participants' assigned intervention during the trial? | | PY | | Because liraglutide was administered by injection and pioglitazone orally, and no masking procedure was described, personnel delivering the interventions were probably aware of allocation. | |
|  | | 2.3 If Y/PY/NI to 2.1 or 2.2: Were there deviations from the intended intervention that arose because of the experimental context? | | PN | | Both groups continued the same metformin regimen and received the same diet and exercise advice. No crossover, unplanned co-intervention, or other trial-context deviation was reported. | |
|  | | 2.4 If Y/PY to 2.3: Were these deviations likely to have affected the outcome? | | NA | | Not applicable because question 2.3 was answered probably no. | |
|  | | 2.5 If Y/PY/NI to 2.4: Were these deviations from intended intervention balanced between groups? | | NA | | Not applicable. | |
|  | | 2.6 Was an appropriate analysis used to estimate the effect of assignment to intervention? | | PY | | The results continued to report 30 participants in each randomized group at week 24, with no reported treatment switching or analysis according to treatment received. | |
|  | | 2.7 If N/PN/NI to 2.6: Was there potential for a substantial impact (on the result) of the failure to analyse participants in the group to which they were randomized? | | NA | | Not applicable because question 2.6 was answered probably yes. | |
|  | | **Risk of bias judgement** | | **Low** | | Despite the open-label design, no important deviations from intended intervention were identified, and the analysis appeared to preserve randomized treatment groups. | |
| **Bias due to missing outcome data** | | 3.1 Were data for this outcome available for all, or nearly all, participants randomized? | | PY | | All 60 randomized participants continued to be represented as 30 participants per group in the reported week-24 tables, and no withdrawals or missing HbA1c measurements were described. | |
|  | | 3.2 If N/PN/NI to 3.1: Is there evidence that result was not biased by missing outcome data? | | NA | | Not applicable because question 3.1 was answered probably yes. | |
|  | | 3.3 If N/PN to 3.2: Could missingness in the outcome depend on its true value? | | NA | | Not applicable. | |
|  | | 3.4 If Y/PY/NI to 3.3: Is it likely that missingness in the outcome depended on its true value? | | NA | | Not applicable. | |
|  | | **Risk of bias judgement** | | **Low** | | The report gives no indication of missing HbA1c outcome data and continues to report the full randomized sample at week 24. | |
| **Bias in measurement of the outcome** | | 4.1 Was the method of measuring the outcome inappropriate? | | N | | HbA1c is a standard objective laboratory biomarker of glycemic control; the method of measurement was appropriate for the outcome. | |
|  | | 4.2 Could measurement or ascertainment of the outcome have differed between intervention groups? | | N | | HbA1c was measured using the same clinical laboratory approach and follow-up schedule across randomized groups; no differential ascertainment procedure was reported. | |
|  | | 4.3 Were outcome assessors aware of the intervention received by study participants? | | NI | | Blinding of the laboratory personnel responsible for HbA1c measurement was not specifically reported. | |
|  | | 4.4 If Y/PY/NI to 4.3: Could assessment of the outcome have been influenced by knowledge of intervention received? | | N | | HbA1c is an objective laboratory measurement; knowledge of treatment allocation would not be expected to materially influence the measured value. | |
|  | | 4.5 If Y/PY/NI to 4.4: Is it likely that assessment of the outcome was influenced by knowledge of intervention received? | | NA | | Not applicable because assessment of HbA1c was judged not to be influenced by knowledge of the intervention. | |
|  | | **Risk of bias judgement** | | **Low** | | HbA1c was measured objectively using comparable laboratory procedures across groups; unreported assessor blinding was unlikely to introduce meaningful measurement bias. | |
| **Bias in selection of the reported result** | | 5.1 Were the data that produced this result analysed in accordance with a pre-specified analysis plan that was finalized before unblinded outcome data were available for analysis? | | NI | | No prospective trial registration, protocol or dated statistical analysis plan specifying the HbA1c analysis was identified in the available report. | |
|  | | 5.2 ... multiple eligible outcome measurements (e.g. scales, definitions, time points) within the outcome domain? | | PN | | HbA1c was evaluated at the fixed 24-week follow-up, and there was no indication that the reported time point or measurement definition was chosen because of the result. | |
|  | | 5.3 ... multiple eligible analyses of the data? | | PN | | No evidence indicated selective choice of the reported HbA1c value from multiple competing statistical analyses. | |
|  | | **Risk of bias judgement** | | **Some concerns** | | The absence of accessible prospective analysis specifications prevents confirmation that the HbA1c result was selected independently of the observed findings. | |
| **Overall bias** | | **Risk of bias judgement** | | **Some concerns** | | Overall risk of bias for the HbA1c result was judged Some concerns. The main uncertainties related to unreported allocation concealment and the absence of accessible prospective analysis specifications. | |

## S3.10 Shimizu 2019

| **Unique ID** | Shimizu 2019 | | **Study ID** | UMIN000022155 | **Assessor** | | Hanwen Zheng; Jieru Han (consensus) |
| --- | --- | --- | --- | --- | --- | --- | --- |
| **Ref/label** | Diabetes Obes Metab. 2019;21(2):285–292. | | **Aim** | Effect of assignment to intervention |  | |  |
| **Experimental** | Dapagliflozin 5 mg/day + standard diabetes care, 24 weeks | | **Comparator** | Standard diabetes care without SGLT2 inhibitor, 24 weeks | **Source** | | https://doi.org/10.1111/dom.13520 |
| **Outcome** | Glycated hemoglobin (HbA1c) | | **Result** | Change from baseline in HbA1c at week 24 (dapagliflozin vs standard treatment). | **Weight** | | 1 |
| **Domain** | | **Signalling question / judgement** | | **Response** | | **Comments / justification** | |
| **Bias arising from the randomization process** | | 1.1 Was the allocation sequence random? | | PY | | Participants were allocated 1:1 using the Pocock–Simon minimization method, stratified by sex, age, and BMI. This was considered probably random for the RoB 2 judgement. | |
|  | | 1.2 Was the allocation sequence concealed until participants were enrolled and assigned to interventions? | | PY | | The report states that patients were randomly allocated after eligibility/enrolment using the prespecified minimization procedure. A separate concealment mechanism was not described, so concealment was judged probably, rather than definitely, adequate. | |
|  | | 1.3 Did baseline differences between intervention groups suggest a problem with the randomization process? | | N | | Baseline characteristics were generally comparable between randomized groups, including glycemic measures where reported. No systematic pattern of imbalance suggested a problem with the randomization process. | |
|  | | **Risk of bias judgement** | | **Low** | | Randomization used a prespecified covariate-adaptive minimization procedure and no systematic baseline imbalance relevant to HbA1c was identified. Allocation concealment was judged probably adequate; therefore this domain was judged Low. | |
| **Bias due to deviations from intended interventions** | | 2.1 Were participants aware of their assigned intervention during the trial? | | Y | | The study used an open-label design, so participants were aware of the assigned treatment. | |
|  | | 2.2 Were carers and people delivering the interventions aware of participants' assigned intervention during the trial? | | Y | | Treating clinicians were aware of allocation because dapagliflozin was added only in the intervention group and standard diabetes treatment was managed clinically. | |
|  | | 2.3 If Y/PY/NI to 2.1 or 2.2: Were there deviations from the intended intervention that arose because of the experimental context? | | N | | No important trial-context deviation from the intended strategies was identified. Intensification of non-SGLT2 glucose-lowering therapy in the control group was explicitly part of the intended standard-treatment strategy for achieving glycaemic targets rather than an unintended deviation caused by awareness of allocation. | |
|  | | 2.4 If Y/PY to 2.3: Were these deviations likely to have affected the outcome? | | NA | | Not applicable because no important deviation from the intended intervention arising because of trial context was identified. | |
|  | | 2.5 If Y/PY/NI to 2.4: Were these deviations from intended intervention balanced between groups? | | NA | | Not applicable because question 2.4 was not required. | |
|  | | 2.6 Was an appropriate analysis used to estimate the effect of assignment to intervention? | | Y | | Participants with available HbA1c data were analysed according to their randomized treatment groups. Missing outcome data are considered separately in Domain 3. | |
|  | | 2.7 If N/PN/NI to 2.6: Was there potential for a substantial impact (on the result) of the failure to analyse participants in the group to which they were randomized? | | NA | | Not applicable because the analysis retained participants in their randomized groups. | |
|  | | **Risk of bias judgement** | | **Low** | | Although the trial was open-label, knowledge of treatment assignment was not shown to cause important deviations from the intended intervention strategies. Protocol-permitted treatment intensification formed part of the intended comparator strategy, and observed participants were analysed by randomized group. | |
| **Bias due to missing outcome data** | | 3.1 Were data for this outcome available for all, or nearly all, participants randomized? | | PN | | HbA1c follow-up data were available for 57 of 63 randomized participants (90.5%): 33/35 in the dapagliflozin group and 24/28 in the control group. This was judged probably not 'all or nearly all' for a strict outcome-specific assessment. | |
|  | | 3.2 If N/PN/NI to 3.1: Is there evidence that result was not biased by missing outcome data? | | PY | | The six non-completions were explained (congestive heart failure, Charcot-joint surgery, no longer meeting criteria, and loss to follow-up). Most reasons were not plausibly related to the participant's unobserved HbA1c value, and the amount of missing data was modest; therefore the HbA1c result was judged probably not materially biased by missingness. | |
|  | | 3.3 If N/PN to 3.2: Could missingness in the outcome depend on its true value? | | NA | | Not applicable because question 3.2 was answered Probably yes. | |
|  | | 3.4 If Y/PY/NI to 3.3: Is it likely that missingness in the outcome depended on its true value? | | NA | | Not applicable because question 3.3 was not required. | |
|  | | **Risk of bias judgement** | | **Low** | | Six of 63 randomized participants lacked follow-up data, but reasons were reported and were predominantly unrelated to HbA1c. Missingness was therefore judged unlikely to materially bias the HbA1c comparison. | |
| **Bias in measurement of the outcome** | | 4.1 Was the method of measuring the outcome inappropriate? | | N | | HbA1c is a standard objective laboratory biomarker of glycemic control; the method of measurement was appropriate for the outcome. | |
|  | | 4.2 Could measurement or ascertainment of the outcome have differed between intervention groups? | | N | | HbA1c was measured using the same clinical laboratory approach and follow-up schedule across randomized groups; no differential ascertainment procedure was reported. | |
|  | | 4.3 Were outcome assessors aware of the intervention received by study participants? | | NI | | Blinding of the laboratory personnel responsible for HbA1c measurement was not specifically reported. | |
|  | | 4.4 If Y/PY/NI to 4.3: Could assessment of the outcome have been influenced by knowledge of intervention received? | | N | | HbA1c is an objective laboratory measurement; knowledge of treatment allocation would not be expected to materially influence the measured value. | |
|  | | 4.5 If Y/PY/NI to 4.4: Is it likely that assessment of the outcome was influenced by knowledge of intervention received? | | NA | | Not applicable because assessment of HbA1c was judged not to be influenced by knowledge of the intervention. | |
|  | | **Risk of bias judgement** | | **Low** | | HbA1c was measured objectively using comparable laboratory procedures across groups; unreported assessor blinding was unlikely to introduce meaningful measurement bias. | |
| **Bias in selection of the reported result** | | 5.1 Were the data that produced this result analysed in accordance with a pre-specified analysis plan that was finalized before unblinded outcome data were available for analysis? | | PY | | The trial was prospectively registered (UMIN000022155) and HbA1c was reported at the fixed 24-week end-of-treatment assessment. Although a standalone dated HbA1c statistical analysis plan was not available, the prospective trial framework, fixed assessment time point, and absence of evidence for result-driven alternative HbA1c analyses were considered sufficient to judge prespecification as probable. | |
|  | | 5.2 ... multiple eligible outcome measurements (e.g. scales, definitions, time points) within the outcome domain? | | PN | | HbA1c was reported at the fixed 24-week follow-up, with no indication that the reported measurement was selected from multiple eligible HbA1c time points because of the findings. | |
|  | | 5.3 ... multiple eligible analyses of the data? | | PN | | No evidence suggested selective choice among multiple eligible statistical analyses of HbA1c. | |
|  | | **Risk of bias judgement** | | **Low** | | HbA1c was assessed at a fixed planned follow-up, and there was no indication that the reported value was selected from multiple eligible measurements or statistical analyses on the basis of the findings. Considering the prospective trial registration and the objective, standard nature of HbA1c, the risk of selective reporting was judged Low. | |
| **Overall bias** | | **Risk of bias judgement** | | **Low** | | Overall risk of bias for the HbA1c result was judged Low. Despite the open-label design, no important intervention deviations were identified, HbA1c was objectively measured, and no material concern was identified in the other domains. | |

## S3.11 Phrueksotsai 2021

| **Unique ID** | Phrueksotsai 2021 | | **Study ID** | TCTR20170511001 | **Assessor** | | Hanwen Zheng; Jieru Han (consensus) |
| --- | --- | --- | --- | --- | --- | --- | --- |
| **Ref/label** | J Gastroenterol Hepatol. 2021;36(10):2952–2959. | | **Aim** | Effect of assignment to intervention |  | |  |
| **Experimental** | Dapagliflozin 10 mg/day + stable oral antidiabetic drugs, 12 weeks | | **Comparator** | Placebo + stable oral antidiabetic drugs, 12 weeks | **Source** | | https://doi.org/10.1111/jgh.15580 |
| **Outcome** | Glycated hemoglobin (HbA1c) | | **Result** | Change from baseline in HbA1c at week 12 (dapagliflozin vs placebo). | **Weight** | | 1 |
| **Domain** | | **Signalling question / judgement** | | **Response** | | **Comments / justification** | |
| **Bias arising from the randomization process** | | 1.1 Was the allocation sequence random? | | Y | | Participants were randomized 1:1 using a computer-generated block randomization procedure. | |
|  | | 1.2 Was the allocation sequence concealed until participants were enrolled and assigned to interventions? | | PY | | A separate allocation-concealment procedure was not described in detail, but the double-blind placebo-controlled design and use of a computer-generated allocation schedule make prior knowledge of the next assignment unlikely; concealment was therefore judged probably adequate. | |
|  | | 1.3 Did baseline differences between intervention groups suggest a problem with the randomization process? | | N | | Baseline characteristics were generally comparable between randomized groups, including glycemic measures where reported. No systematic pattern of imbalance suggested a problem with the randomization process. | |
|  | | **Risk of bias judgement** | | **Low** | | The computer-generated randomized allocation, probably adequate concealment, and balanced baseline characteristics supported a Low risk judgement. | |
| **Bias due to deviations from intended interventions** | | 2.1 Were participants aware of their assigned intervention during the trial? | | N | | The study was double-blinded and placebo-controlled; participants were not expected to know whether they received dapagliflozin or placebo. | |
|  | | 2.2 Were carers and people delivering the interventions aware of participants' assigned intervention during the trial? | | N | | Investigators/treating personnel were blinded to treatment assignment in the placebo-controlled design. | |
|  | | 2.3 If Y/PY/NI to 2.1 or 2.2: Were there deviations from the intended intervention that arose because of the experimental context? | | NA | | Not applicable because participants and people delivering the intervention were judged not to be aware of treatment assignment. | |
|  | | 2.4 If Y/PY to 2.3: Were these deviations likely to have affected the outcome? | | NA | | Not applicable because question 2.3 was not required. | |
|  | | 2.5 If Y/PY/NI to 2.4: Were these deviations from intended intervention balanced between groups? | | NA | | Not applicable because question 2.4 was not required. | |
|  | | 2.6 Was an appropriate analysis used to estimate the effect of assignment to intervention? | | Y | | Participants with available HbA1c data were compared in their randomized groups; there was no indication that participants were reclassified according to treatment received. | |
|  | | 2.7 If N/PN/NI to 2.6: Was there potential for a substantial impact (on the result) of the failure to analyse participants in the group to which they were randomized? | | NA | | Not applicable because the analysis retained the randomized-group comparison. | |
|  | | **Risk of bias judgement** | | **Low** | | The double-blind placebo-controlled design minimized awareness-related deviations, and the HbA1c comparison preserved randomized treatment groups. | |
| **Bias due to missing outcome data** | | 3.1 Were data for this outcome available for all, or nearly all, participants randomized? | | PN | | Thirty-eight of 40 randomized participants (95%) completed the 12-week study (18/20 dapagliflozin; 20/20 placebo), so outcome data were available for nearly all but not literally all randomized participants. | |
|  | | 3.2 If N/PN/NI to 3.1: Is there evidence that result was not biased by missing outcome data? | | PY | | Only two participants lacked follow-up, both in the dapagliflozin group. The small amount of missing data was judged unlikely to materially alter the HbA1c comparison, with no evidence that loss to follow-up depended on the unobserved HbA1c value. | |
|  | | 3.3 If N/PN to 3.2: Could missingness in the outcome depend on its true value? | | NA | | Not applicable because question 3.2 was answered Probably yes. | |
|  | | 3.4 If Y/PY/NI to 3.3: Is it likely that missingness in the outcome depended on its true value? | | NA | | Not applicable because question 3.3 was not required. | |
|  | | **Risk of bias judgement** | | **Low** | | HbA1c outcome data were available for 95% of randomized participants and the small amount of missing data was unlikely to materially bias the result. | |
| **Bias in measurement of the outcome** | | 4.1 Was the method of measuring the outcome inappropriate? | | N | | HbA1c is a standard objective laboratory biomarker of glycemic control; the method of measurement was appropriate for the outcome. | |
|  | | 4.2 Could measurement or ascertainment of the outcome have differed between intervention groups? | | N | | HbA1c was measured using the same clinical laboratory approach and follow-up schedule across randomized groups; no differential ascertainment procedure was reported. | |
|  | | 4.3 Were outcome assessors aware of the intervention received by study participants? | | PN | | The study was double-blind and placebo-controlled, so laboratory/outcome assessors were probably unaware of treatment allocation. | |
|  | | 4.4 If Y/PY/NI to 4.3: Could assessment of the outcome have been influenced by knowledge of intervention received? | | N | | HbA1c is an objective laboratory measurement and would not be expected to be influenced by knowledge of allocation. | |
|  | | 4.5 If Y/PY/NI to 4.4: Is it likely that assessment of the outcome was influenced by knowledge of intervention received? | | NA | | Not applicable because assessment of HbA1c was judged not to be influenced by knowledge of the intervention. | |
|  | | **Risk of bias judgement** | | **Low** | | HbA1c was measured objectively using comparable laboratory procedures across groups; unreported assessor blinding was unlikely to introduce meaningful measurement bias. | |
| **Bias in selection of the reported result** | | 5.1 Were the data that produced this result analysed in accordance with a pre-specified analysis plan that was finalized before unblinded outcome data were available for analysis? | | PY | | The trial was prospectively registered (TCTR20170511001) and HbA1c was reported at the fixed 12-week end-of-treatment assessment. Although a standalone dated HbA1c statistical analysis plan was not available, the prospective trial framework, fixed assessment time point, and absence of evidence for result-driven alternative HbA1c analyses were considered sufficient to judge prespecification as probable. | |
|  | | 5.2 ... multiple eligible outcome measurements (e.g. scales, definitions, time points) within the outcome domain? | | PN | | HbA1c was reported at the fixed 12-week endpoint, and no evidence suggested that the result was selected from multiple eligible HbA1c measurements on the basis of the findings. | |
|  | | 5.3 ... multiple eligible analyses of the data? | | PN | | The report provides no indication that the HbA1c result was selected from multiple competing analyses according to statistical significance or effect magnitude. | |
|  | | **Risk of bias judgement** | | **Low** | | HbA1c was assessed at a fixed planned follow-up, and there was no indication that the reported value was selected from multiple eligible measurements or statistical analyses on the basis of the findings. Considering the prospective trial registration and the objective, standard nature of HbA1c, the risk of selective reporting was judged Low. | |
| **Overall bias** | | **Risk of bias judgement** | | **Low** | | Overall risk of bias for the HbA1c result was judged Low. The blinded randomized design, minimal missing data, objective HbA1c measurement, and absence of evidence for selective reporting supported a Low overall judgment. | |

# Supplementary Table S4. GRADE evidence profile for co-primary outcomes: SGLT2 inhibitors plus background therapy vs Background therapy alone

| **Author(s)** | | | | | | | Hanwen Zheng; Jieru Han | | | | | | |  |
| --- | --- | --- | --- | --- | --- | --- | --- | --- | --- | --- | --- | --- | --- | --- |
| **Question** | | | | | | | SGLT2 inhibitors plus background therapy compared to Background therapy alone for Asian adults with type 2 diabetes mellitus and metabolic dysfunction-associated steatotic liver disease | | | | | | |  |
| **Setting** | | | | | | | Clinical settings in Asian countries | | | | | | |  |
| **Certainty assessment** | | | | | | | | **No. of patients** | | **Effect** | | **Certainty** | **Importance** | |
| **№ of studies** | **Study design** | **Risk of bias** | **Inconsistency** | **Indirectness** | **Imprecision** | **Other considerations** | | **SGLT2 inhibitors plus background therapy** | **Background therapy alone** | **Relative (95% CI)** | **Absolute (95% CI)** | **Certainty** | **Importance** | |
| **Alanine aminotransferase (follow-up: range 12 weeks to 24 weeks; assessed with: Change from baseline in serum ALT (U/L))** | | | | | | | | | | | | | | |
| 5 | randomised trials | seriousa | not serious | not serious | not seriousb | none | | 139 | 126 | - | MD 7.56 U/L lower (11.24 lower to 3.88 lower) | ⨁⨁⨁◯ Moderatea,b | IMPORTANT | |
| **Glycated hemoglobin (follow-up: range 12 weeks to 24 weeks; assessed with: Change from baseline in HbA1c (%))** | | | | | | | | | | | | | | |
| 5 | randomised trials | seriousa | not serious | not serious | not seriousc | none | | 139 | 126 | - | MD 0.09 % lower (0.27 lower to 0.08 higher) | ⨁⨁⨁◯ Moderatea,c | IMPORTANT | |
|  |  |  |  |  |  |  | |  |  |  |  |  |  | |

*Note: Numbers of patients in this GRADE profile are outcome-specific analyzed denominators and may differ from the numbers randomized or treated.*
Formal GRADE assessment was restricted to the co-primary outcomes (ALT and HbA1c); secondary outcomes were considered exploratory and were not formally graded.
For imprecision, 95% confidence intervals were interpreted against explicit thresholds (17 U/L for ALT and 0.5 percentage points for HbA1c) together with the optimal information size (OIS). OIS calculations used a two-sided α = 0.05, 80% power, and the pooled within-group SD of change scores. The 17-U/L ALT threshold is a contextual histology-associated response threshold, not a validated between-group MID.

CI: confidence interval; MD: mean difference

**Explanations**

a. Two of the five contributing randomized results were judged to have some concerns in the final outcome-specific RoB 2 assessments. The concerns involved the randomization process, missing outcome data, and/or selection of the reported result; deviations from intended interventions and outcome measurement were judged at low risk. Certainty was therefore downgraded by one level for risk of bias.

b. Imprecision was not downgraded for ALT. The 95% CI (−11.24 to −3.88 U/L) did not cross the contextual −17-U/L histology-associated response threshold, and the accrued information (n = 265) exceeded the calculated OIS of 29 participants. This supports a modest biochemical effect below that contextual threshold, not evidence of histological benefit.

c. Imprecision was not downgraded for HbA1c. The 95% CI (−0.27 to 0.08 percentage points) remained entirely within the ±0.5-percentage-point threshold for a clinically important between-group difference, and the accrued information (n = 265) exceeded the calculated OIS of 91 participants.

# Supplementary Table S5. GRADE evidence profile for co-primary outcomes: GLP-1 receptor agonists plus background therapy vs Background therapy alone

| **Author(s)** | | | | | | | Hanwen Zheng; Jieru Han | | | | | | |  |
| --- | --- | --- | --- | --- | --- | --- | --- | --- | --- | --- | --- | --- | --- | --- |
| **Question** | | | | | | | GLP-1 receptor agonists plus background therapy compared to Background therapy alone for Asian adults with type 2 diabetes mellitus and metabolic dysfunction-associated steatotic liver disease | | | | | | |  |
| **Setting** | | | | | | | Clinical settings in Asian countries | | | | | | |  |
| **Certainty assessment** | | | | | | | | **No. of patients** | | **Effect** | | **Certainty** | **Importance** | |
| **№ of studies** | **Study design** | **Risk of bias** | **Inconsistency** | **Indirectness** | **Imprecision** | **Other considerations** | | **GLP-1 receptor agonists plus background therapy** | **Background therapy alone** | **Relative (95% CI)** | **Absolute (95% CI)** |  |  | |
| **Alanine aminotransferase (follow-up: range 20 weeks to 26 weeks; assessed with: Change from baseline in serum ALT (U/L))** | | | | | | | | | | | | | | |
| 2 | randomised trials | serious a | not serious | not serious | not serious b | none | | 58 | 55 | - | MD 6.71 U/L lower (11.53 lower to 1.89 lower) | ⨁ ⨁ ⨁ ◯ Moderate a , b | IMPORTANT | |
| **Glycated hemoglobin (follow-up: range 20 weeks to 26 weeks; assessed with: Change from baseline in HbA1c (percentage points))** | | | | | | | | | | | | | | |
| 2 | randomised trials | serious a | not serious | not serious | serious c | none | | 58 | 55 | - | MD 0.48 % lower (0.83 lower to 0.14 lower) | ⨁ ⨁ ◯ ◯ Low a , c | IMPORTANT | |
|  |  |  |  |  |  |  | |  |  |  |  |  |  | |

*Note: Numbers of patients in this GRADE profile are outcome-specific analyzed denominators and may differ from the numbers randomized or treated.*
Formal GRADE assessment was restricted to the co-primary outcomes (ALT and HbA1c); secondary outcomes were considered exploratory and were not formally graded.
For imprecision, 95% confidence intervals were interpreted against explicit thresholds (17 U/L for ALT and 0.5 percentage points for HbA1c) together with the optimal information size (OIS). OIS calculations used a two-sided α = 0.05, 80% power, and the pooled within-group SD of change scores. The 17-U/L ALT threshold is a contextual histology-associated response threshold, not a validated between-group MID.
Explanations
a. One of the two contributing randomized results (Guo 2020) was judged to have some concerns in the final outcome-specific RoB 2 assessment, related to selection of the reported result; the other contributing trial was judged at low risk. Given the contribution of the result with some concerns to this small evidence base, certainty was downgraded by one level for risk of bias.
b. Imprecision was not downgraded for ALT. The 95% CI (−11.53 to −1.89 U/L) did not cross the contextual −17-U/L histology-associated response threshold, and the accrued information (n = 113) exceeded the calculated OIS of 43 participants. This does not imply histological benefit.
c. Downgraded one level for HbA1c imprecision because the 95% CI (−0.83 to −0.14 percentage points) crossed the −0.5-percentage-point clinically important benefit threshold, spanning both a potentially important reduction and a smaller effect. The accrued information (n = 113) was also marginally below the calculated OIS of 114 participants.

# Supplementary Table S6. GRADE evidence profile for co-primary outcomes: SGLT2 inhibitors plus background therapy vs TZD plus background therapy

| **Author(s)** | | | | | | | Hanwen Zheng; Jieru Han | | | | | | |  |
| --- | --- | --- | --- | --- | --- | --- | --- | --- | --- | --- | --- | --- | --- | --- |
| **Question** | | | | | | | SGLT2 inhibitors plus background therapy compared to TZD plus background therapy for Asian adults with type 2 diabetes mellitus and metabolic dysfunction-associated steatotic liver disease | | | | | | |  |
| **Setting** | | | | | | | Clinical settings in Asian countries | | | | | | |  |
| **Certainty assessment** | | | | | | | | **No. of patients** | | **Effect** | | **Certainty** | **Importance** | |
| **№ of studies** | **Study design** | **Risk of bias** | **Inconsistency** | **Indirectness** | **Imprecision** | **Other considerations** | | **SGLT2 inhibitors plus background therapy** | **TZD plus background therapy** | **Relative (95% CI)** | **Absolute (95% CI)** |  |  | |
| **Alanine aminotransferase (follow-up: range 24 weeks to 28 weeks; assessed with: Change from baseline in serum ALT (U/L))** | | | | | | | | | | | | | | |
| 3 | randomised trials | not serious | not serious | not serious | not serious | none | | 101 | 103 | - | MD 1.26 U/L higher (2.50 lower to 5.01 higher) | ⨁ ⨁ ⨁ ⨁ High ^a^ |  | |
| **Glycated hemoglobin (follow-up: range 24 weeks to 28 weeks; assessed with: Change from baseline in HbA1c (percentage points))** | | | | | | | | | | | | | | |
| 3 | randomised trials | not serious | not serious | not serious | not serious | none | | 101 | 103 | - | MD 0.02 % higher (0.21 lower to 0.25 higher) | ⨁ ⨁ ⨁ ⨁ High ^b^ |  | |

*Note: Numbers of patients in this GRADE profile are outcome-specific analyzed denominators and may differ from the numbers randomized or treated.*

Formal GRADE assessment was restricted to the co-primary outcomes (ALT and HbA1c); secondary outcomes were considered exploratory and were not formally graded.
For imprecision, 95% confidence intervals were interpreted against explicit thresholds (17 U/L for ALT and 0.5 percentage points for HbA1c) together with the optimal information size (OIS). OIS calculations used a two-sided α = 0.05, 80% power, and the pooled within-group SD of change scores. The 17-U/L ALT threshold is a contextual histology-associated response threshold, not a validated between-group MID.
Risk-of-bias rationale: One of the three contributing trials (Ito 2017) was judged to have some concerns for both co-primary outcomes, whereas Kinoshita 2020 and Attaran 2023 were judged at low risk. These concerns were not considered sufficient to materially lower confidence in the pooled estimates; risk of bias was therefore judged not serious.
Explanations
a. Imprecision was not downgraded for ALT. The 95% CI (−2.50 to 5.01 U/L) remained well within the ±17-U/L contextual threshold for a clinically important between-treatment difference, and the accrued information (n = 204) exceeded the calculated OIS of 40 participants.
b. Imprecision was not downgraded for HbA1c. The 95% CI (−0.21 to 0.25 percentage points) remained entirely within the ±0.5-percentage-point clinically important threshold, and the accrued information (n = 204) exceeded the calculated OIS of 100 participants.

# Supplementary Table S7. GRADE evidence profile for co-primary outcomes: GLP-1 receptor agonists plus background therapy vs TZD plus background therapy

| **Author(s)** | | | | | | | Hanwen Zheng; Jieru Han | | | | | | |  |
| --- | --- | --- | --- | --- | --- | --- | --- | --- | --- | --- | --- | --- | --- | --- |
| **Question** | | | | | | | GLP-1 receptor agonists plus background therapy compared to TZD plus background therapy for Asian adults with type 2 diabetes mellitus and metabolic dysfunction-associated steatotic liver disease | | | | | | |  |
| **Setting** | | | | | | | Clinical settings in Asian countries | | | | | | |  |
| **Certainty assessment** | | | | | | | | **No. of patients** | | **Effect** | | **Certainty** | **Importance** | |
| **№ of studies** | **Study design** | **Risk of bias** | **Inconsistency** | **Indirectness** | **Imprecision** | **Other considerations** | | **GLP-1 receptor agonists plus background therapy** | **TZD plus background therapy** | **Relative (95% CI)** | **Absolute (95% CI)** |  |  | |
| **Glycated hemoglobin (follow-up: mean 24 weeks; assessed with: Change from baseline in HbA1c (percentage points))** | | | | | | | | | | | | | | |
| 1 | randomised trials | serious a | not serious | not serious | serious b | none | | 30 | 30 | - | MD 0.40 % lower (1.02 lower to 0.22 higher) | ⨁ ⨁ ◯ ◯ Low ^a , b^ | IMPORTANT | |
| **Alanine aminotransferase (follow-up: mean 24 weeks; assessed with: Change from baseline in serum ALT (U/L))** | | | | | | | | | | | | | | |
| 1 | randomised trials | serious a | not serious | not serious | not serious c | none | | 30 | 30 | - | MD 9.9 U/L lower (14.8 lower to 5 lower) | ⨁ ⨁ ⨁ ◯ Moderate ^a , c^ | IMPORTANT | |
|  |  |  |  |  |  |  | |  |  |  |  |  |  | |

*Note: Numbers of patients in this GRADE profile are outcome-specific analyzed denominators and may differ from the numbers randomized or treated.*
Formal GRADE assessment was restricted to the co-primary outcomes (ALT and HbA1c); secondary outcomes were considered exploratory and were not formally graded.
For imprecision, 95% confidence intervals were interpreted against explicit thresholds (17 U/L for ALT and 0.5 percentage points for HbA1c) together with the optimal information size (OIS). OIS calculations used a two-sided α = 0.05, 80% power, and the pooled within-group SD of change scores. The 17-U/L ALT threshold is a contextual histology-associated response threshold, not a validated between-group MID.
Explanations
a. The single contributing randomized trial (Zhang 2020) was judged to have some concerns for both co-primary outcomes, related to the randomization process and selection of the reported result; certainty was downgraded by one level for risk of bias.
b. Downgraded one level for HbA1c imprecision because the 95% CI (−1.02 to 0.22 percentage points) crossed the −0.5-percentage-point clinically important benefit threshold and also included little or no important difference. The accrued information (n = 60) was below the calculated OIS of 190 participants.
c. Imprecision was not downgraded for ALT. The 95% CI (−14.80 to −5.00 U/L) did not cross the contextual −17-U/L histology-associated response threshold, and the accrued information (n = 60) exceeded the calculated OIS of 11 participants. This supports a biochemical effect below that contextual threshold, not evidence of histological benefit.

# Supplementary Table S8. Detailed study characteristics, treatment regimens, participant populations, follow-up, and outcomes of included randomized controlled trials

| **Study, year, country** | **Design, treatment/background therapy, and follow-up** | **Key eligibility and NAFLD/MASLD ascertainment** | **Participant populations and outcomes contributed to meta-analysis** |
| --- | --- | --- | --- |
| Ito et al. (2017) [20] Japan | Multicentre (3 sites), open-label, active-controlled RCT;  Treatment: Ipragliflozin 50 mg/day + background therapy; comparator: pioglitazone 15–30 mg/day + background therapy; follow-up: 24 weeks. | Adults aged 20-75 years with T2D and NAFLD suggested by liver tests or CT/ultrasonography; HbA1c 7.0-11.0%; BMI <=45 kg/m²; other liver disease and excess alcohol excluded. | Population — randomized: 32/34; treated/safety: NR; analyzed: 32/34 (ITT denominators).  ALT, AST, GGT, HbA1c, FPG, lipids, BMI/body weight, adiponectin, SBP. |
| Kinoshita et al. (2020) [21] Japan | Multicentre (7 sites), prospective, open-label, 3-arm active-controlled RCT;  Treatment: Dapagliflozin 5 mg/day + background therapy; comparator: pioglitazone 7.5–15 mg/day + background therapy; follow-up: 28 weeks. | Adults with T2D, HbA1c >=6.5%, BMI >=22 kg/m², sex-specific ALT elevation, stable diabetes medication for >=1.5 months, and CT L/S ratio <1.0; other liver disease and excess alcohol excluded. | Population — randomized: 40/36 (eligible arms); treated/safety: NR; analyzed: 32/33.  ALT, AST, GGT, HbA1c, FPG, lipids, BMI/body weight, adiponectin,fibrosis indices. |
| Shi et al. (2023) [22] China | Single-centre, prospective, open-label RCT;  Treatment: Dapagliflozin 10 mg/day + metformin-based background therapy; comparator: matched metformin-based background therapy without dapagliflozin; follow-up: 24 weeks. | Adults aged 18-75 years with T2D inadequately controlled on metformin >=1.5 g/day for >=8 weeks, HbA1c 7.0-10.0%, BMI 24-45 kg/m², and MRI-PDFF >=5.5%; other liver disease and excess alcohol excluded. | Population — randomized: 42/42; treated/safety: NR; analyzed: 40/38.  MRI-PDFF liver fat, FIB-4, ALT, AST, GGT, HbA1c/FPG, lipids, BMI, IL-6. |
| Kuchay et al. (2020; D-LIFT) [23] India | Single-centre, open-label, parallel-group RCT with concealed allocation;  Treatment: Dulaglutide 0.75 mg weekly for 4 weeks, then 1.5 mg weekly for 20 weeks + standard diabetes care; comparator: standard diabetes care without a GLP-1 receptor agonist; follow-up: 24 weeks. | Adults with T2D, HbA1c >7.0%, and MRI-PDFF liver fat >=6.0%; background treatment could include metformin, DPP-4 inhibitors, sulfonylurea, and/or insulin; competing liver disease and liver-fat-modifying drugs excluded. | Population — randomized: 32/32; treated/safety: NR; analyzed: 27/25.  MRI-PDFF liver fat, liver stiffness, ALT, AST, GGT, HbA1c/FPG, lipids, BMI/body weight. |
| Attaran et al. (2023) [24] Iran | Randomized, single-blind, parallel-group clinical trial;  Treatment: Empagliflozin 10 mg/day + background therapy; comparator: pioglitazone 30 mg/day + background therapy; follow-up: 24 weeks. | Adults aged 20-80 years with T2D, HbA1c 7.0-10.5%, CAP >=302 dB/m, no established ASCVD, and stable standard antidiabetic therapy for >=6 months; cirrhosis, other liver disease, excess alcohol, and eGFR <45 excluded. | Population — randomized: 37/36; treated/safety: NR; analyzed: 37/36 (ITT).  liver stiffness, FIB-4/NFS, ALT/AST, HbA1c, lipids, BMI, SBP and other metabolic measures. |
| Kuchay et al. (2018; E-LIFT) [25] India | Single-centre, open-label RCT;  Treatment: Empagliflozin 10 mg/day + background therapy; comparator: background diabetes therapy; follow-up: 20 weeks. | Adults with T2D and NAFLD, with MRI-PDFF liver fat >=6.0%, requiring intensification of glucose-lowering treatment; other causes of liver disease and excess alcohol excluded. | Population — randomized: 25/25; treated/safety: NR; analyzed: 22/20.  MRI-PDFF liver fat, ALT, AST, GGT, HbA1c/FPG, lipids, BMI/body weight. |
| Guo et al. (2020) [26] China | Randomized, placebo-controlled, 3-arm trial;  Eligible pairwise comparison: liraglutide 1.8 mg/day + metformin-based background therapy versus placebo + matched metformin-based background therapy; follow-up: 26 weeks. | Obese adults with T2D and NAFLD inadequately controlled on metformin; competing liver disease and relevant confounding treatments excluded. | Population — randomized: 32/32 for the eligible liraglutide/placebo arms; treated/safety: NR; analyzed: 31/30.  Intrahepatic lipid, ALT/AST/GGT, HbA1c/FPG, lipids, BMI/body weight. |
| Horibe et al. (2022) [27] Japan | Single-centre, randomized, open-label, parallel-group trial;  Treatment: Dapagliflozin 5 mg/day + standard treatment; comparator: standard treatment without an SGLT2 inhibitor; follow-up: 24 weeks. | Adults aged 20-75 years with T2D, HbA1c 7.0-10.0%, BMI >=23 kg/m², and treatment with oral glucose-lowering agents. | Population — randomized: 52 total (arm-level randomized count NR in the supplied study information); treated/safety: NR; analyzed: 26/24.  Intrahepatic triglyceride content, liver enzymes and prespecified glycaemic, lipid, blood-pressure and anthropometric outcomes. |
| Zhang et al. (2020) [28] China | Single-centre, open-label, prospective, randomized, parallel-group trial;  Treatment: Liraglutide 0.6 mg/day (week 1), then 1.2 mg/day (weeks 2–24) + metformin + diet/exercise advice; comparator: pioglitazone 15 mg/day (week 1), then 30 mg/day (weeks 2–24) + metformin + diet/exercise advice; follow-up: 24 weeks. | Adults with T2D and NAFLD; hepatic fat was quantified by proton MRS. Other causes of chronic liver disease and relevant confounding therapies were excluded. | Population — randomized: 30/30; treated/safety: NR; analyzed: 30/30.  Hepatic fat, ALT/AST/GGT, HbA1c/FPG, lipids, BMI/body weight. |
| Shimizu et al. (2019) [29] Japan | Randomized, open-label, active-controlled trial;  Treatment: Dapagliflozin 5 mg/day + standard diabetes care; comparator: standard diabetes care without an SGLT2 inhibitor; follow-up: 24 weeks. | Adults with T2D and NAFLD; hepatic steatosis and stiffness were assessed by transient elastography; other major liver diseases and excess alcohol were excluded. | Population — randomized: 35/28; treated/safety: NR; analyzed: 33/24.  LSM, ALT, AST/GGT, HbA1c/FPG, lipids, BMI/body weight. |
| Phrueksotsai et al. (2021) [30] Thailand | Single-centre, double-blind, placebo-controlled RCT;  Treatment: Dapagliflozin 10 mg/day + stable oral antidiabetic drugs; comparator: placebo + stable oral antidiabetic drugs; follow-up: 12 weeks. | Adults with T2D and NAFLD, HbA1c 7.0-10.0%, receiving standard diabetes treatment; major alternative liver diseases and excess alcohol were excluded. | Population — randomized: 20/20; treated/safety: NR; analyzed: 18/20.  Hepatic fat, ALT/AST/GGT and prespecified glycaemic, lipid. |

*Population definitions: randomized = participants allocated to the eligible comparison arms; treated/safety = participants who initiated assigned treatment or were included in a defined safety population when explicitly reported; analyzed = participants contributing to the efficacy outcome analysis used in the meta-analysis. Treated/safety counts were recorded as NR when the original study did not separately report them and were not inferred. Across eligible comparison arms, 692 participants were randomized and 642 contributed to the co-primary efficacy analyses. For multi-arm trials, counts refer only to arms contributing to the prespecified pairwise comparison.*

Abbreviations: ALT, alanine aminotransferase; ASCVD, atherosclerotic cardiovascular disease; AST, aspartate aminotransferase; BMI, body mass index; CAP, controlled attenuation parameter; CT, computed tomography; DPP-4, dipeptidyl peptidase-4; FIB-4, fibrosis-4 index; FPG, fasting plasma glucose; GGT, gamma-glutamyl transferase; HbA1c, glycated hemoglobin; L/S ratio, liver-to-spleen attenuation ratio; LSM, liver stiffness measurement; MRI, magnetic resonance imaging; MRI-PDFF, magnetic resonance imaging-proton density fat fraction; MRS, magnetic resonance spectroscopy; NAFLD, nonalcoholic fatty liver disease; NFS, NAFLD fibrosis score; RCT, randomized controlled trial; SBP, systolic blood pressure; SGLT2, sodium-glucose cotransporter 2; T2D, type 2 diabetes.

# Supplementary Table S8A. Arm-level treatment, background therapy, and protocol-specified stability/adjustment

| **Study** | **Experimental arm** | **Comparator arm** | **Background therapy (dose when reported)** | **Protocol-specified stability/adjustment during follow-up** |
| --- | --- | --- | --- | --- |
| Ito et al. (2017) [20] | Ipragliflozin 50 mg/day + background therapy | Pioglitazone 15–30 mg/day + same background therapy | Pre-existing antidiabetes regimen; individual background agents/doses NR. | No additions or routine adjustments to background antidiabetes therapy were permitted. Insulin or sulfonylurea could be reduced/discontinued only to avoid hypoglycemia; no sulfonylurea reductions occurred and insulin-dose changes did not differ significantly between groups. Diet/exercise counselling was the same in both arms. |
| Kinoshita et al. (2020) [21] | Dapagliflozin 5 mg/day + background therapy | Pioglitazone 7.5–15 mg/day + background therapy | Diabetes medication stable for ≥1.5 months before randomization; individual background agents/doses NR. | Concomitant antidiabetic medications were intended to remain unchanged; any dose adjustment followed the study protocol. No important differential deviation was reported. |
| Shi et al. (2023) [22] | Dapagliflozin 10 mg/day + metformin | Metformin without dapagliflozin | Metformin ≥1.5 g/day for ≥8 weeks before randomization. | Both groups continued the same metformin-based background management, with identical diet/exercise advice and follow-up schedule. No unplanned differential co-intervention was reported. |
| Kuchay et al. (2020; D-LIFT) [23] | Dulaglutide 0.75 mg weekly for 4 weeks, then 1.5 mg weekly for 20 weeks + standard diabetes care | Standard diabetes care without a GLP-1 receptor agonist | Background therapy could include metformin, DPP-4 inhibitors, sulfonylurea, and/or insulin; individual background-drug doses NR. | Lifestyle advice and diabetes management were protocol-guided. Background treatment could be adjusted clinically; discontinuation of DPP-4 inhibitors in the dulaglutide arm was protocol-specified. No crossover or unplanned differential co-intervention was reported. |
| Attaran et al. (2023) [24] | Empagliflozin 10 mg/day + background diabetes care | Pioglitazone 30 mg/day + background diabetes care | Stable standard antidiabetic therapy for ≥6 months before randomization; individual background agents/doses NR. | Background antihyperglycemic therapy could be adjusted according to the same ADA guidance in both arms; randomized study-drug doses remained constant. Both groups received standard lifestyle advice. |
| Kuchay et al. (2018; E-LIFT) [25] | Empagliflozin 10 mg/day + standard diabetes care | Standard diabetes care without an SGLT2 inhibitor | Standard diabetes care; specific background agents/doses NR in the available report. | Background glucose-lowering treatment could be adjusted to maintain glycaemic equipoise as part of the planned strategy. No treatment crossover or unplanned differential co-intervention was reported. |
| Guo et al. (2020) [26] | Liraglutide 1.8 mg/day + metformin | Placebo + metformin | Metformin; exact background metformin dose NR in the available report. | Metformin was maintained at a constant dose in all groups, and lifestyle advice was the same. Study-drug titration was protocol-driven; no unplanned differential co-intervention or crossover was reported. |
| Horibe et al. (2022) [27] | Dapagliflozin 5 mg/day + standard oral glucose-lowering treatment | Continuation of previous oral glucose-lowering treatment / standard treatment | Oral glucose-lowering agents; individual background agents/doses NR. | Sulfonylurea dose reduction in the dapagliflozin arm and possible addition of non-SGLT2 agents in the control arm were prespecified components of the treatment strategy. No important unplanned deviation was reported. |
| Zhang et al. (2020) [28] | Liraglutide 0.6 mg/day (week 1), then 1.2 mg/day (weeks 2–24) + metformin | Pioglitazone 15 mg/day (week 1), then 30 mg/day (weeks 2–24) + metformin | Same metformin regimen in both arms; metformin dose NR. | Metformin was continued in both groups, with the same diet/exercise advice. No crossover, unplanned co-intervention, or other trial-context deviation was reported. |
| Shimizu et al. (2019) [29] | Dapagliflozin 5 mg/day + standard diabetes care | Standard diabetes care without an SGLT2 inhibitor | Standard non-SGLT2 glucose-lowering therapy; individual background agents/doses NR. | Intensification of non-SGLT2 glucose-lowering therapy in the control arm was explicitly part of the prespecified standard-treatment strategy for achieving glycaemic targets, rather than an unplanned co-intervention. |
| Phrueksotsai et al. (2021) [30] | Dapagliflozin 10 mg/day + stable OADs | Placebo + stable OADs | Stable oral antidiabetic drugs; specific agents/doses NR. | Background OADs were described as stable. The trial was double-blind and placebo-controlled, and no differential background-therapy change was reported. |

# Supplementary Table S9. Adverse events and treatment discontinuation in the included studies

|  | **Adverse events reported** | **Serious/severe adverse events** | **Discontinuation due to adverse events** | **Other discontinuations and reporting limitations** |
| --- | --- | --- | --- | --- |
| Ito et al., 2017 | Ipragliflozin: 3 urinary tract infections and 1 vaginal candidiasis; all resolved with treatment and study therapy was continued. Pioglitazone: 2 cases of edema. | No serious adverse events were reported in either group. | Pioglitazone: 2; ipragliflozin: 0. | Ipragliflozin: 1 voluntary withdrawal and 1 loss to follow-up; pioglitazone: 1 loss to follow-up. No severe hypoglycemia, dehydration, ketoacidosis, heart failure, or serious infection requiring withdrawal was reported. |
| Kinoshita et al., 2020 | Specific adverse events were listed in Supplementary Table 2, which was unavailable; event types and arm-level counts could not be extracted. | No severe adverse events were reported in any group. | NR. | A total of 101 participants completed the trial and 98 were included in the final analysis; arm-level safety data were not available. |
| Shi et al., 2023 | The authors reported “no significant adverse events” without providing event types or arm-level counts. | NR; the statement “no significant adverse events” was not interpreted as evidence that no serious adverse events occurred. | NR. | Dapagliflozin: 1 bariatric surgery and 1 loss to follow-up. Control: 1 case with extensive missing data, 1 treatment discontinuation, and 2 losses to follow-up; the reason for treatment discontinuation was unclear. |
| Kuchay et al., 2020, D-LIFT | Dulaglutide: 5 cases of upper gastrointestinal discomfort, including 3 leading to treatment discontinuation and 2 resolving with treatment; 1 additional case of transient diarrhea. Control: 2 cases of upper gastrointestinal symptoms, both resolved. | No serious drug-related adverse events were reported. | Dulaglutide: 3/32; control: 0/32. | All-cause discontinuation was 5/32 with dulaglutide (3 gastrointestinal events and 2 losses to follow-up) and 7/32 in the control group (5 losses to follow-up and 2 protocol deviations). |
| Kuchay et al., 2018, E-LIFT | Empagliflozin: 1 balanoposthitis, 1 nonspecific fatigue, and 1 arthralgia; symptoms improved after discontinuation. No study-related adverse events were reported in the control group. | Serious adverse events were not reported as a separate category. | Empagliflozin: 3/25; control: 0/25. | Twenty-two participants in the empagliflozin group and 20 in the control group completed treatment. Five control participants withdrew for reasons unrelated to adverse events; the original report contained a minor inconsistency regarding a work-schedule-related withdrawal. |
| Attaran et al., 2023 | Nine non-severe adverse events were reported, including cystitis, gross hematuria, minor hypoglycemia, and lower-extremity edema; arm-level distribution was unavailable. | Two severe adverse events—cholangitis and transient ischemic attack—required hospitalization and resolved completely; treatment-related causality was unclear. | NR. | Three participants did not complete the study because of non-adherence (1 in the pioglitazone group and 2 in the empagliflozin group). Detailed arm-level adverse-event data were available only in an unavailable supplement. |
| Guo et al., 2020 | Insulin glargine: mild hypoglycemia 3/30 and nausea or vomiting 2/30. Liraglutide: nausea or vomiting 8/31, diarrhea 1/31, and non-severe hypoglycemia 1/31. Placebo: nausea or vomiting 1/30 and non-severe hypoglycemia 1/30. | Deaths: 0; severe adverse events: 0; severe hypoglycemia: 0. | No participant discontinued treatment because of an adverse event. | Ninety-one of 96 randomized participants completed the trial. The remaining withdrawals were attributed to relocation, loss to follow-up, or financial reasons rather than adverse events. |
| Horibe et al., 2022 | Hypoglycemia, dehydration, urinary tract infection, and exanthema occurred in both groups; arm-level counts were reported only in unavailable Supplemental Table S1. | NR. | Dapagliflozin: 1 participant discontinued because of exanthema and hypoglycemia; whether follow-up was completed was unclear. | Two control participants withdrew before the protocol began because of a personal reason and an incidental pancreatic tumor. One additional control participant stopped standard therapy because of pemphigoid but remained under follow-up. |
| Zhang et al., 2020 | Liraglutide: gastrointestinal reactions 9/30, hypoglycemia 1/30, heart failure 0/30, and any adverse reaction 10/30. Pioglitazone: gastrointestinal reactions 2/30, hypoglycemia 2/30, heart failure 1/30, and any adverse reaction 5/30. Gastrointestinal reactions were more frequent with liraglutide. | No severe hypoglycemia occurred. Overall serious adverse events were not systematically reported, and the single heart-failure event in the pioglitazone group was not explicitly classified as serious or treatment-related. | NR. | Withdrawal, treatment discontinuation, and loss to follow-up were not described; safety analyses used 30 participants per group, so treatment completion could not be independently verified. |
| Shimizu et al. | Specific adverse-event types and counts were not reported. | Serious adverse events were not formally reported. One control participant did not complete the study because of congestive heart failure; causality was unclear. | Could not be determined. No adverse-event-related discontinuation was clearly reported in the dapagliflozin group; congestive heart failure led to non-completion in one control participant. | Dapagliflozin: 33/35 completed (1 no longer met eligibility criteria and 1 loss to follow-up). Control: 24/28 completed (1 congestive heart failure, 1 surgery for Charcot joint, 1 no longer eligible, and 1 loss to follow-up). |
| Phrueksotsai et al., 2021 | The authors reported “no significant adverse events” without providing event types or arm-level counts. | Serious adverse events were not reported separately. | No adverse-event-related treatment discontinuation was reported. | Two participants in the dapagliflozin group declined further follow-up after week 4 and were lost to follow-up; there was no evidence that these withdrawals were related to adverse events. |

*Denominator note: Safety-event denominators are reproduced as reported by the original trials and should not be assumed to equal the randomized or efficacy-analysis population. A treated/safety population was recorded as NR when the original report did not clearly define it; no treated denominator was inferred.*

Abbreviations: AE, adverse event; NR, not reported. Statements such as “no significant adverse events” were retained as reported by the original authors and were not interpreted as indicating that no adverse events occurred. A clinical condition resulting in study non-completion was not classified as treatment-related unless the original study explicitly reported such a relationship.

# Supplementary Figure S1

*Funnel plots are provided for exploratory visualization only because each meta-analysis included fewer than 10 studies; they should not be used to infer the absence of publication bias.*

|  |  |
| --- | --- |

**Supplementary Figure S1.** Publication bias and sensitivity analyses for the effect of SGLT2 inhibitors versus background therapy on alanine aminotransferase (ALT). (A) Funnel plot with pseudo 95% confidence limits. (B) Leave-one-out sensitivity analysis assessing the robustness of the pooled effect estimate.

# Supplementary Figure S2

|  |  |
| --- | --- |

**Supplementary Figure S2.** Publication bias and sensitivity analyses for the effect of SGLT2 inhibitors versus background therapy on aspartate aminotransferase (AST). (A) Funnel plot with pseudo 95% confidence limits. (B) Leave-one-out sensitivity analysis assessing the robustness of the pooled effect estimate.

# Supplementary Figure S3

|  |  |
| --- | --- |

**Supplementary Figure S3.** Publication bias and sensitivity analyses for the effect of SGLT2 inhibitors versus background therapy on glycated hemoglobin (HbA1c). (A) Funnel plot with pseudo 95% confidence limits. (B) Leave-one-out sensitivity analysis assessing the robustness of the pooled effect estimate.

# Supplementary Figure S4

|  |  |
| --- | --- |

**Supplementary Figure S4.** Publication bias and sensitivity analyses for the effect of SGLT2 inhibitors versus background therapy on fasting plasma glucose (FPG). (A) Funnel plot with pseudo 95% confidence limits. (B) Leave-one-out sensitivity analysis assessing the robustness of the pooled effect estimate.

# Supplementary Figure S5

|  |  |
| --- | --- |

**Supplementary Figure S5.** Publication bias and sensitivity analyses for the effect of SGLT2 inhibitors versus background therapy on triglycerides (TG). (A) Funnel plot with pseudo 95% confidence limits. (B) Leave-one-out sensitivity analysis assessing the robustness of the pooled effect estimate.

# Supplementary Figure S6

|  |  |
| --- | --- |

**Supplementary Figure S6.** Publication bias and sensitivity analyses for the effect of SGLT2 inhibitors versus background therapy on high-density lipoprotein cholesterol (HDL-C). (A) Funnel plot with pseudo 95% confidence limits. (B) Leave-one-out sensitivity analysis assessing the robustness of the pooled effect estimate.

# Supplementary Figure S7

|  |  |
| --- | --- |

**Supplementary Figure S7.** Publication bias and sensitivity analyses for the effect of SGLT2 inhibitors versus background therapy on low-density lipoprotein cholesterol (LDL-C). (A) Funnel plot with pseudo 95% confidence limits. (B) Leave-one-out sensitivity analysis assessing the robustness of the pooled effect estimate.

# Supplementary Figure S8

**Supplementary Figure S8.** HbA1c-specific risk-of-bias graph. Percentages represent the review authors’ RoB 2 judgments across the 11 randomized results contributing to HbA1c. Green indicates low risk, yellow indicates some concerns, and red indicates high risk; the target estimand was the effect of assignment to intervention (intention-to-treat).

# Supplementary Figure S9

**Supplementary Figure S9.** HbA1c-specific study-level risk-of-bias summary. Symbols show the review authors’ judgments for each RoB 2 domain and the overall judgment for each included study. Green plus signs indicate low risk, yellow question marks indicate some concerns, and red minus signs indicate high risk.
